# Harvest of waterfowl and Sandhill Crane in rural Alaska: Geographic and seasonal patterns

**DOI:** 10.1371/journal.pone.0307135

**Published:** 2024-07-25

**Authors:** Liliana C. Naves, Jason L. Schamber

**Affiliations:** 1 Division of Subsistence, Alaska Department of Fish and Game, Anchorage, Alaska, United States of America; 2 Division of Wildlife Conservation, Alaska Department of Fish and Game, Anchorage, Alaska, United States of America; MARE – Marine and Environmental Sciences Centre, PORTUGAL

## Abstract

We estimated the annual harvest of waterfowl and Sandhill Crane *Grus canadensis* and their eggs by Alaska’s rural residents and described seasonal and geographic patterns. Subsistence in Alaska refers to patterns of resource use typical of rural, remote regions where Indigenous people are a high proportion of the population. Rural communities in Alaska rely on the legally-allowed spring-summer harvest of migratory birds for food and socio-cultural wellbeing, in addition to harvests in the fall-winter general hunting season. We based harvest estimates on a large dataset (637 community-years) composed from multiple sources. The estimated annual average harvest of waterfowl and Sandhill Crane by rural residents was 270,641 birds/year (68% in spring-summer, 32% in fall-winter) and 36,692 eggs/year in the 2004–2015 reference period. Harvest estimates for ducks, swans, and Sandhill Crane were lower than in the 1980s–1990s. Harvest amounts, seasonality, and species composition distinguished regional patterns for the Pacific-Aleutian mainland and islands, Bering Sea mainland, St. Lawrence-Diomede islands, North Slope, and Interior Alaska-Upper Copper River. Rural residents accounted for 79% of the total waterfowl harvest in Alaska and high proportions of the total Pacific Flyway harvest for several species of sea ducks, geese, swans, and Sandhill Crane. Alaska’s Indigenous people are important partners in harvest management and conservation of migratory birds. Harvest data are needed to inform efficient and appropriate decisions to achieve management goals. This study can facilitate collaboration for harvest management and conservation across Alaska and the flyways by helping diverse users to understand their contributions to the total harvest.

## Introduction

As migratory birds are shared by diverse user groups, management of their harvests involves coordination at the local, regional, and flyway levels by engaging federal, state, and tribal governments as well as non-governmental organizations in the United States (U.S.) and abroad [1, 2]. For administrative and coordination purposes, Alaska belongs to the Pacific Flyway, together with other 11 western states. Alaska provides healthy and productive habitats for millions of breeding and migrating waterfowl and Sandhill Crane *Grus canadensis* from migratory flyways in the Americas and beyond [3]. The productive waters and coastal habitats of the Bering Sea and North Pacific Ocean surrounding Alaska also support wintering waterfowl, especially sea ducks and Emperor Goose *Anser canagicus* [4, 5].

The objective of this study was to estimate the harvest of waterfowl and Sandhill Crane and their eggs for all rural regions in Alaska including breakdown by seasons. We combined multi-year data to portray the annual average harvest for the 2004–2015 period in ways that are easily accessible and usable by managers, researchers, resource users, and other stakeholders. We discussed geographic and seasonal harvest patterns in the context of current management and conservation priorities. Data on the amounts, seasonality, and species composition of subsistence harvests help users to understand their contributions to the total harvest and support participation of Indigenous people in management and conservation for migratory birds [2, 6, 7]. Harvest data are also important to understand the socio-ecological relevance of Alaska’s biological resources and to inform harvest management at the region, state, and flyway levels. This study provides a foundation to inform and enable collaboration in harvest management.

Annual bird harvest monitoring in parts of rural Alaska started in the 1980s, during the Yukon-Kuskokwim Delta Goose Management Plan [8–12]. The annual harvest survey of the Alaska Migratory Bird Co-Management Council (AMBCC) started in 2004 and expanded harvest monitoring to other regions [2, 13]. Bird harvest data also have been collected as part of broader surveys in selected communities and years [e.g., 14–16]. Harvest estimates have been available mostly at the community and region levels. Diverse geographic scales for reporting and annual variation in the estimates make it difficult to understand the Alaska-wide harvest. Previous studies estimated Alaska-wide waterfowl harvest for the 1980s–1990s, but the available dataset did not enable estimates of bird and egg harvest detailed at the species level and by season [17–19]. These previous studies highlighted that waterfowl and Sandhill Crane represented about 90% of the annual bird harvest in rural Alaska, including large proportions of the total harvest in the Pacific Flyway for Emperor Goose (endemic to the Bering Sea), Brant *Branta bernicla*, and several species of sea ducks.

### Socio-economic and regulatory context

For millennia, Alaska Native, Indigenous people—Inupiaq; St. Lawrence Island Yupik; Central Yup’ik; Athabascan; Alutiiq-Sugpiaq and Unangax̂-Aleut; and Eyak, Tlingit, Haida, and Tsimshian—have harvested wild foods, including many species of birds, throughout the year following their seasonal availability [17]. In Alaska, subsistence means a way of life centered on customary, non-commercial uses of wild resources for food, shelter, fuel, clothing, tools, crafts, transportation, sharing, and bartering (AS 16.05.940.34, ANILCA-Title VIII section 803). This definition derives from the values, traditions, and local economies of Indigenous people and other rural residents who share similar resource use patterns. Subsistence encompasses a mixed harvest-cash economy based on a domestic mode of production, seasonal cycles of harvests, networks of distribution and exchange of wild foods, traditional land use patterns, beliefs, and worldviews [20]. These patterns of resource use are typical of remote Alaska communities with a high proportion of Indigenous residents (5 AAC 99.025 (12) (I)) [18, 21–23]. The harvest sector of this economy is more reliable and stable than the cash sector. Communities in rural Alaska tend to have depressed employment rates and a high prevalence of seasonal-only and low-income jobs, which contrast with the high cost of living.

Wild foods are rich in nutrients and support a healthier diet than processed foods [24]. Wild foods provided 189% of the daily protein needs and 26% of the daily caloric needs for rural Alaska residents in the early 2000s [25]. Subsistence harvests in rural Alaska amount to 36.9 million edible pounds per year including fish (53%), land and marine mammals (23% and 14%, respectively), plants (4%), shellfish (3%), and birds and eggs (3%) [25]. Although birds and eggs are a small part of the total harvest in weight, they are harvested primarily in spring, when other resources are scarce. Bird and egg harvests also add diversity to the diet and are socially and culturally important [17].

Historically, the arrival of migratory birds in spring alleviated hunger and starvation in Northern Indigenous communities. Wild foods stored in the previous harvest season were depleted in spring, after a long Arctic winter. Enforcement of the spring-summer harvest closure established by the 1918 Migratory Bird Treaty Act (MBTA) caused hardships for Indigenous communities in Alaska as they depended on wild foods for survival [26]. In 1997, following decades of efforts by Indigenous leaders, the bird treaties with Canada and Mexico were amended to legally allow the Alaska spring-summer subsistence harvest of migratory birds [2, 27, 28]. The Alaska Migratory Bird Co-Management Council (AMBCC) was created in 2000 to provide a meaningful role for Indigenous people in harvest management and conservation of migratory birds [29]. The AMBCC has three voting partners: the U.S. Fish and Wildlife Service (USFWS), the Alaska Department of Fish and Game (ADF&G), and the Native Caucus with Indigenous representatives from regions across Alaska. In 2018, the USFWS and the ADF&G presented a joint apology to Alaska Indigenous people for unintended harm resulting from insensitive past harvest regulations for the harvest of migratory birds [30]. Efforts to build trust continue, but relationships between Indigenous subsistence users and fish and wildlife agencies in Alaska still are tenuous.

Harvest regulations for waterfowl and other migratory birds in Alaska include a spring-summer subsistence season (2 April–31 August; 50 CFR 92) in addition to a fall-winter general hunting season (1 September–26 January, 107-day season dates vary by hunt zone; 50 CFR 20) [31, 32]. Bird harvest regulations in Alaska do not refer to user ethnicity. Eligibility to participate in the spring-summer subsistence harvest of migratory birds is based on regions or communities of residence that have spring-summer harvest traditions and excludes unqualified urban areas. For urban Alaska residents from Euro-American cultural traditions, contemporary bird hunting usually evolved from recreational pursuits [33]. Alaska’s urban communities are supported by a capital economy, where families earn income in wage-market employment and food is primarily imported from southern agricultural sources.

Some aspects remain unsettled about the rules for where and when migratory birds can be legally harvested in Alaska. First, regulations for migratory bird harvest largely differ between the two regulatory seasons and are substantially less restrictive in the spring-summer season. Second, there are no legal provisions to accommodate subsistence harvest of migratory birds in rural regions in the fall-winter season. Restrictive regulations in the fall-winter season contrast with traditional harvest practices of Indigenous communities. Harvest data for all regulatory seasons are important to inform decisions that can help harmonize values and goals of diverse stakeholders.

## Materials and methods

### Study area

Alaska’s vast geography (665,400 mi^2^) includes marine, coastal, wetland, boreal forest, and other ecosystems in Arctic and sub-Arctic western North America. A few urban centers concentrate more than half of the state’s human population (about 730,000 people). Approximately 250 small communities are remote and accessible only by aircraft, boat, or in winter by snowmobile. About half of the population in the remote communities are Indigenous [21].

**Fig 1:**
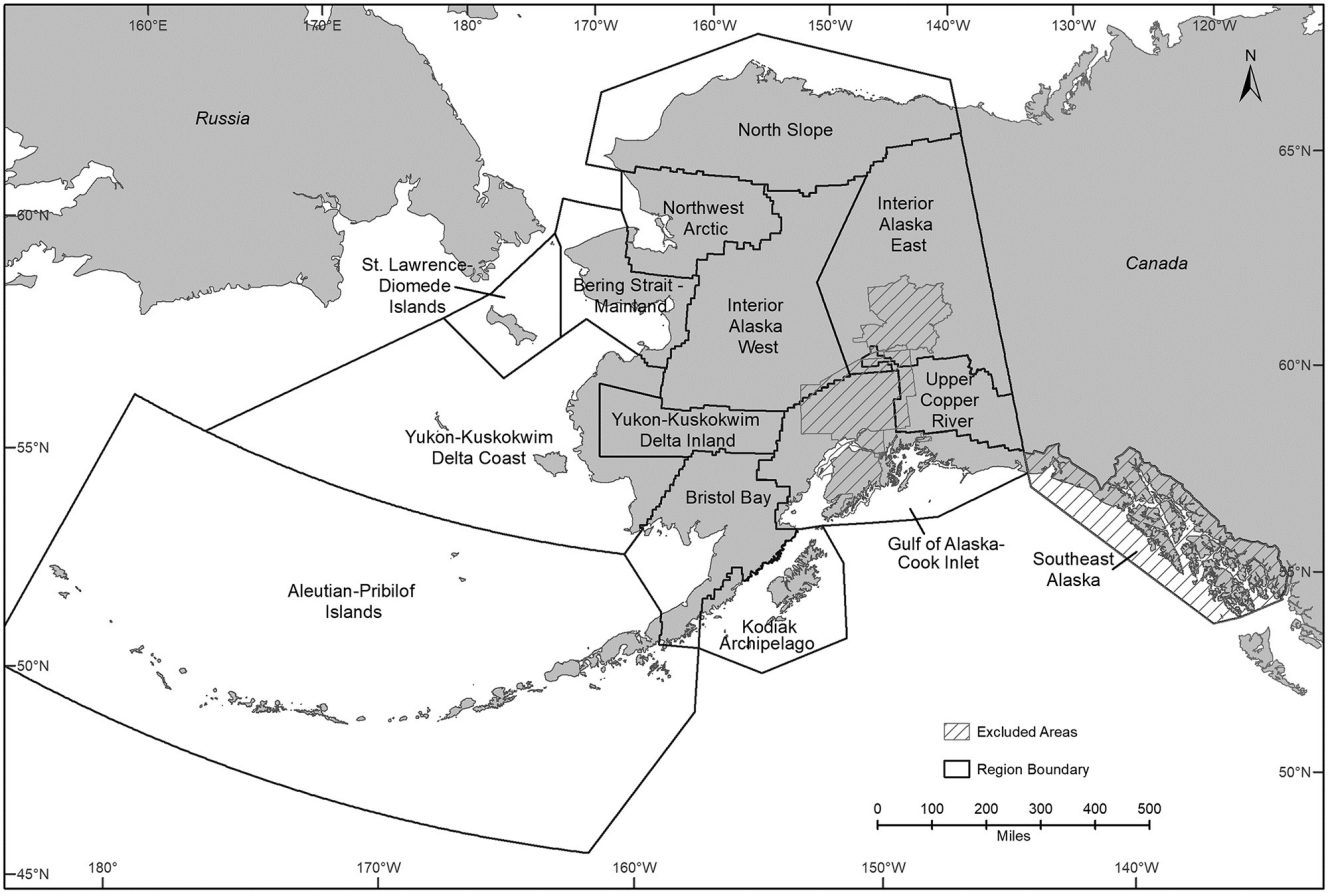
Alaska’s rural regions as defined in this study. Adapted from management regions for the spring-summer subsistence harvest of migratory birds [31].

In this study we used the term “rural” to refer to the communities and regions eligible to participate in the Alaska spring-summer subsistence harvest of migratory birds, and we used the term “urban” to refer to the communities and regions ineligible to participate in this harvest (Fig 1) (50 CFR 92.5) [31]. This geographic framework adopted for harvest management reflects relevant socio-ecological and regulatory domains. The eligible area includes 202 communities with a total population of about 87,000 people during the 1990s–2010s (S1 Table in S1 File) [21]. We divided the Yukon-Kuskokwim Delta (Y-K Delta) management region into “Coast” and “Inland” and the Bering Strait-Norton Sound region into “Mainland” and “St. Lawrence-Diomede Islands” because of distinct patterns in waterfowl species composition and abundance. We combined the Gulf of Alaska and Cook Inlet management regions because of similar waterfowl species composition and abundance (Fig 1). We did not report waterfowl harvests for Southeast Alaska because in this region the resources opened for harvest in the spring-summer subsistence season include only eggs of the Glaucous-winged Gull *Larus glaucescens*.

This study represented harvests based on the region of residence of hunters. We assumed that harvests by rural residents largely happened within the boundaries of their region of residence. Surveys in rural communities documented harvests by year-round resident households, including harvests that may have happened outside the boundaries of their community of residence. For Indigenous people in Alaska, land and resources are organized into defined geographic areas to which access and use are traditionally allocated among kinship groups. As the Indigenous populations (originally semi-nomadic) re-organized into sedentary communities, land use areas evolved into community-use areas [20]. Kinship-based land use patterns now overlap with and are somewhat affected by modern socio-economic and political boundaries including those defined by harvest management systems. In contrast, land use patterns of Alaska urban residents (mostly south-central Alaska, Fairbanks, and Juneau) often involve harvest areas largely separated from their community and region of residence. Urban residents can harvest in rural regions in the fall-winter general hunting season and often do so, especially at locations on the Alaska Peninsula, Aleutian-Pribilof Islands, and Kodiak Archipelago (e.g., Adak, Saint Paul, Cold Bay, Unalaska, Larsen Bay, and Kodiak City).

### Data sources and treatment

We compiled previously available harvest data for waterfowl and Sandhill Crane for rural regions to estimate an annual average harvest in the 2004–2015 reference period, which is consistent with changes in harvest regulations starting in 2003 that legally allowed the spring-summer subsistence harvest of migratory birds. The basic data unit was a community-year, i.e., a harvest survey conducted in a specific community and year. The dataset (637 community-years) included data from the AMBCC harvest survey (416 community-years) [34], Community Subsistence Information System (CSIS) from the ADF&G Division of Subsistence (164 community-years) [16], and other sources (57 community-years) [14, 15, 35–39].

The ADF&G does not have a formal institutional review board for social sciences research. Nevertheless, the ADF&G Division of Subsistence has conducted social sciences research applied to resource management in collaboration with local communities throughout Alaska for about 45 years, contributing more than 600 research products in this field. Senior staff reviewed and approved research methods for this study following technical and ethical standards [40, 41]. Additional approval of research methods was not required, especially as this study did not involve new data collection.

Data collection in the multiple used sources followed ethical principles including informed consent, voluntary participation, anonymity, confidentiality, and community review of draft findings [40, 41]. In all sources, data were collected in household surveys collaboratively conducted with Indigenous organizations and local research assistants. Participation in the surveys was voluntary for communities and households. Community consent was usually formalized as written tribal council resolutions. Consent was also obtained from all individual households. Participation of households selected for sampling was typically above 80%.

We broadly shared draft results of this study with federal, state, and Native AMBCC partners asking for their review. A four-page summary of results was also produced to facilitate review. We updated the draft manuscript considering all review input.

Two-thirds of the dataset (492 community-years) pertained to the 2004–2015 reference period (S1 Table, S2 Fig in S1 File). We also included some older (110 community-years in 1982–2003) and more recent data (35 community-years in 2016–2020) to supplement information for less-often surveyed regions. Of the 202 communities in the sampling frame, only nine communities (4%) in four regions were not represented in the dataset.

We also compiled published harvest data for the fall-winter general hunting season from the Harvest Information Program (HIP), which is managed by the USFWS in collaboration with state wildlife agencies [42, 43]. All hunters (residents of Alaska rural and urban regions, other U.S. states, and other countries) intending to harvest waterfowl in the spring-summer or fall-winter hunting seasons in Alaska are required to purchase a state waterfowl conservation tag, also known as the state duck stamp. State stamp holders may be selected for the HIP harvest survey. Finally, we also compiled unpublished data on issued Alaska state duck stamps, which are managed by the ADF&G Division of Administrative Services.

We excluded some available harvest data because of incompatibility among sources and other analytical constraints: (a) AMBCC data affected by missing data issues; (b) most community-years without information on season of harvest; and (c) 1989–1992 surveys in the Gulf of Alaska-Cook Inlet and Kodiak Archipelago (supplemental, older data), as usual harvest patterns were likely disrupted immediately following the 1989 Exxon Valdez oil spill [44]. With the intent of focusing on the data that most accurately represented community harvests, we also excluded community-years for which the sampling proportion was less than 45% of the total households or the sample size was smaller than 45 households (including AMBCC data for 2016 and later when methodological changes reduced sampling rates at the community level [45]). Surveys in small communities usually had sampling rates above 45% (and nearing a census), thus this approach did not cause their under-representation in the dataset. We acknowledge this was a high data-quality threshold, but it was enabled by a relatively large quantity of data available. We also excluded data prior to 1997 from regions for which more recent data were available (S1 Table in S1 File); this threshold intended to represent current harvest patterns and reduce overlap with the 1980–1996 period represented in previously available harvest estimates [19].

We limited the Emperor Goose data to 1987–2016 (609 community-years; years outside the 2004–2015 reference period consisted of limited supplemental data for regions less often surveyed), when harvest of this species was closed due to its low abundance [46–48]. We did not estimate Emperor Goose harvest for the period after the harvest was re-opened in 2017 because data were unavailable for some regions and some data were incompatible with the analytical approach used in this study.

The household harvest surveys used in this study relied on species identification by respondents and did not employ biological sampling (e.g., a parts collection survey [49]). Species identification issues may result from challenges in distinguishing look-alike species and differences between genetics-based taxonomy and local ethno-taxonomies (e.g., female eiders and scoters). Household harvest surveys in Alaska use multi-species categories because of diverse study foci, species identification issues, and a need for conciseness in surveys involving dozens to hundreds of animal and plant species. We standardized data from the original sources into the following multi-species categories: wigeons, teals, scaups, goldeneyes, mergansers, Canada/Cackling geese, and swans. We reserved the use of plural in species names to indicate these multi-species categories. Favoring conciseness, the surveys often did not explicitly ask about harvest of uncommon species such as Redhead *Aythya americana* and Ruddy Duck *Oxyura jamaicensis*, which hunters may only occasionally encounter. A few records of Redhead harvest were included in the category “ducks (other, unidentified).”

We divided the year into spring (April–June: pre-breeding migration, arrival at breeding grounds, egg laying, and incubation), summer (July–August: chick rearing), and fall-winter (September–March: post-breeding migration and wintering) broadly reflecting seasonal phenology and availability of migratory birds in Alaska. We presented only annual egg harvest estimates, as eggs are available for about a month in spring-summer in any given location.

### Data analysis

We followed analytical methods developed in previous studies [50–52]. We calculated harvest estimates by extrapolating data from surveyed households and communities to represent all households and communities in the rural regions. First, we calculated community-level harvest for the AMBCC data based on household raw data (S3 Appendix-equation 1 in S1 File). Then, we combined these harvest estimates with the other data sources that reported at the community level. Although we used a large dataset, data were insufficient to estimate Alaska-wide harvest for individual years while properly accounting for geographic patterns. For communities surveyed more than once, we averaged annual harvest and variance at community level before calculating region-wide estimates. Thus, estimates represented an annual average harvest in the 2004–2015 period (i.e., harvest was not summed over years). At the region level, we extrapolated community-level estimates to account for the few communities not represented in the dataset (S3 Appendix-equation 2 in S1 File). But for most regions, all communities were represented in the dataset. We then summed region estimates into Alaska-wide estimates. Harvest estimates did not account for wounding loss (birds struck but not retrieved).

We calculated community-level variance for the AMBCC survey from household raw data (S3 Appendix-equations 3.a and 3.b in S1 File). For other sources, we retro-calculated community-level variance based on reported confidence intervals assuming that all surveys used simple random sampling (equation 3.c in S1 File). We calculated region-level variance using formulas for two-stage sampling: communities were primary sampling units and households were secondary sampling units (S3 Appendix-equations 4.a–4.c in S1 File; [50]). We summed region-level variance into Alaska-wide variance. We calculated confidence intervals as percentages of harvest estimates (S3 Appendix-equations 5.a and 5.b in S1 File). We analyzed data using the IBM Statistical Package for the Social Sciences (SPSS) version 27.

## Results

### Harvest amounts and species composition

**Fig 2:**
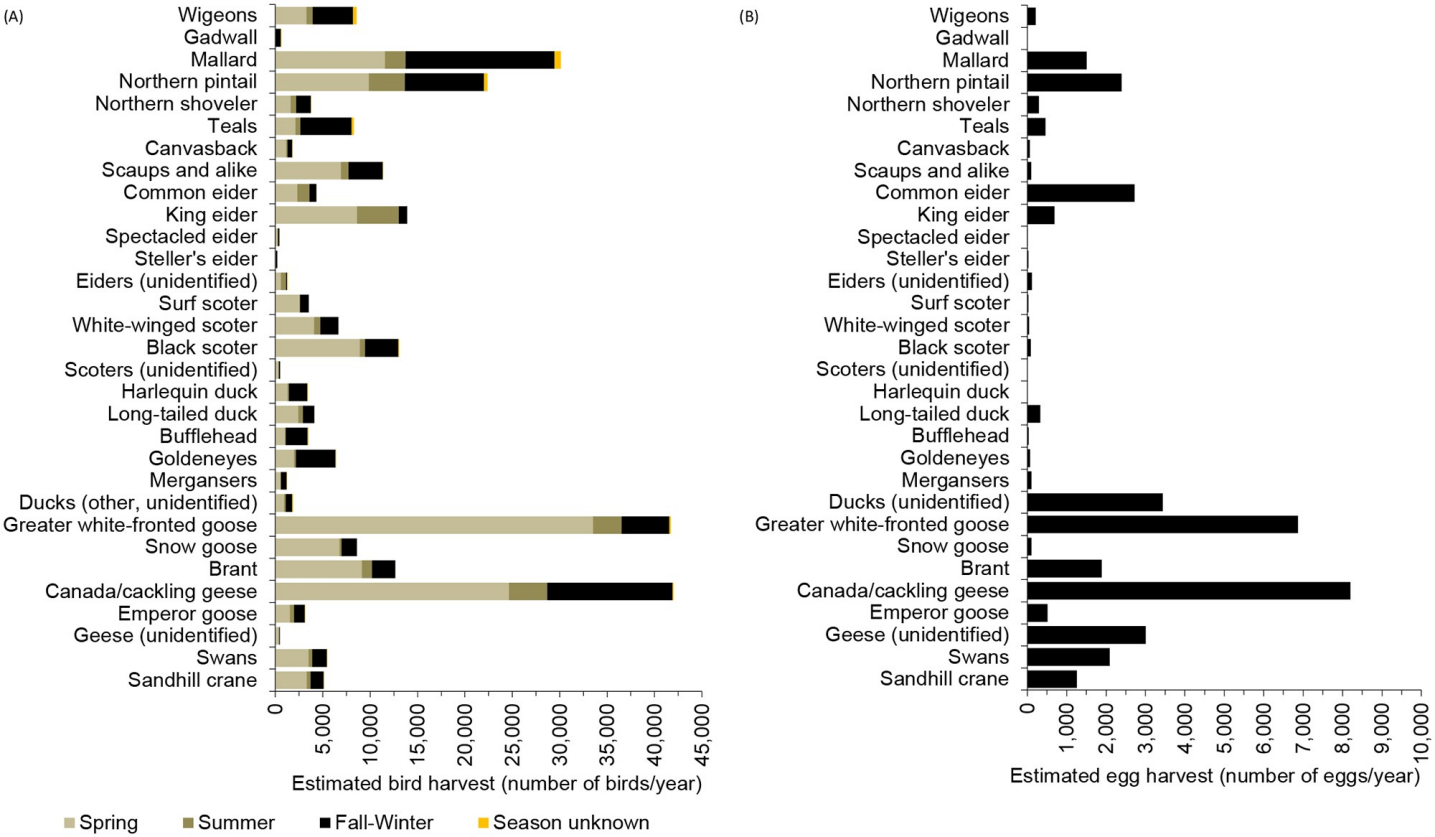
Species composition and amounts of waterfowl and Sandhill Crane (A) and their eggs (B) harvested by rural hunters in Alaska, annual average 2004–2015.

The estimated annual average harvest of waterfowl and Sandhill Crane by rural hunters in Alaska was 270,641 birds/year in the 2004–2015 period (Tables 1–3, Fig 2A, S4 Table in S1 File). The seasonal distribution of the harvest was 58% in spring, 10% in summer, and 32% in fall-winter (for harvests with documented seasons; Table 3, S5 Fig in S1 File). The species/categories that accounted for at least 5% of the waterfowl and Sandhill Crane harvest included: Canada/Cackling geese (16%), White-fronted Goose *Anser albifrons* (15%), Mallard *Anas platyrhynchos* (11%), scoters (9%), Northern Pintail *Anas acuta* (8%), King Eider *Somateria spectabilis* (5%), Black Scoter *Melanitta americana* (5%), and Brant (5%) (Table 2, Fig 2A).

**Table 1:** Overview of annual average harvest and abundance index for waterfowl and Sandhill Crane that regularly breed or occur in Alaska, 2004–2015.

Species that regularly breed or occur in Alaska	Alaska abundance index	Harvest (birds/year)						
		Alaska rural^a^				Alaska HIP survey^b^	Alaska total^a^^,^^b^	Lower 48 Pacific Flyway^b^
		Spring-summer	Fall-winter	Unknown season	Annual	Fall-winter	Annual	Fall-winter
Wigeons	-	3,914	4,300	349	8,563	10,536	19,099	397,606
American Wigeon *Mareca americana*	803,699	-	-		-	10,492	-	396,104
Eurasian Wigeon *M*. *penelope*	-	-	-		-	44	-	1,502
Gadwall *M*. *strepera*	2,009	5	586	48	639	996	1,635	186,437
Mallard *Anas platyrhynchos*	540,343	13,715	15,738	659	30,112	20,833	50,945	958,689
Northern Pintail *A*. *acuta*	1,033,099	13,662	8,340	385	22,387	7,391	29,778	263,975
Northern Shoveler *Spatula clypeata*	462,228	2,181	1,578	28	3,787	2,771	6,558	272,446
Teals	-	2,623	5,435	198	8,256	7,530	15,786	568,224
Green-winged Teal *Anas crecca*	686,727	-	-	-	-	7,465	-	515,442
Blue-winged Teal *Spatula discors*	1,642	-	-	-	-	65	-	52,782
Canvasback *Aythya valisineria*	62,093	1,274	525	2	1,801	153	1,954	24,547
Redhead *A*. *americana*†	1,718	-	-		-	52	52	21,216
Scaups	855,557	7,711	3,635	25	11,371	2,090	13,461	105,977
*Greater Scaup *Aythya marila*	-	-	-	-	-	735	-	16,505
Lesser Scaup *A*. *affinis*	-	-	-	-	-	595	-	36,410
Ring-necked Duck *A*. *collaris*	48,674	-	-	-	-	760	-	53,062
Common Eider *Somateria mollissima*	8,300	3,592	739	5	4,336	-	-	-
*King Eider *S*. *spectabilis*	20,269	13,008	898	0	13,906	-	-	-
Common and King eiders	-	16,600	1,637	5	18,242	67	18,309	0
*‡Spectacled Eider *S*. *fischeri*	20,971	296	119	0	415	0	415	0
*‡Steller’s Eider *Polysticta stelleri*	86,282	69	133	0	202	0	202	0
Alaska-breeding population	149	-	-	-	-	-	-	-
Pacific population	86,282	-	-	-	-	-	-	-
Eiders (unidentified)	-	1,116	126	91	1,333	0	1,333	0
Scoters	321,070	17,145	6,490	141	23,776	3,505	27,281	4,648
Surf Scoter *M*. *perspicillata*	-	2,616	895	11	3,522	2,032	5,554	3,310
White-winged Scoter *M*. *deglandi*	-	4,724	1,912	33	6,669	878	7,547	1,255
*Black Scoter *Melanitta americana*	-	9,457	3,542	72	13,071	595	13,666	83
Scoters (unidentified)	-	348	141	25	514	0	514	0
Harlequin Duck *Histrionicus histrionicus*	-	1,412	1,943	105	3,460	1,577	5,037	83
Long-tailed Duck *Clangula hyemalis*	124,884	2,880	1,211	22	4,113	230	4,343	295
Bufflehead *Bucephala albeola*	49,991	1,083	2,326	66	3,475	1,338	4,813	37,107
Goldeneyes	50,705	2,149	4,199	43	6,391	2,887	9,278	29,715
Common Goldeneye *Bucephala clangula*	-	-	-	-	-	1,111	-	26,942
Barrow’s Goldeneye *B*. *islandica*	-	-	-	-	-	1,776	-	2,773
Mergansers	29,036	573	640	12	1,225	1,940	3,165	10,443
Common Merganser *Mergus merganser*	-	-	-	-	-	1,013	-	4,560
Red-breasted Merganser *M*. *serrator*	-	-	-	-	-	921	-	524
Hooded Merganser *Lophoytes cucullatus*	-	-	-	-	-	6	-	5,359
Ruddy Duck *Oxyura jamaicensis*	224	-	-	-	-	7	7	9,235
Ducks (other, unidentified)	-	1,080	707	87	1,874	0	1,874	0
Greater White-fronted Goose *Anser albifrons*	941,899	36,509	5,065	150	41,724	818	42,542	66,064
Mid-continent population	269,139	-	-	-	-	-	-	-
Pacific population	628,950	-	-	-	-	-	-	-
*Tule *A*. *a*. *elgasi*	14,445	-	-	-	-	-	-	-
Lesser Snow Goose *Anser caerulescens*	213,809	7,000	1,600	0	8,600	4	8,604	66,074
Western Arctic population	31,328	-	-	-	-	-	-	-
Wrangell Island population	166,750	-	-	-	-	-	-	-
Ross’s Goose *Anser rossi*	-	-	-	-	-	28	28	13,208
*Brant *Branta bernicla*	150,200	10,187	2,446	6	12,639	1,342	13,981	1,733
Canada/Cackling geese	-	28,665	13,252	91	42,008	6,370	48,378	293,351
Minima Cackling Goose *Branta hutchinsii minima*	273,458	-	-	-	-	-	-	-
Aleutian Cackling Goose *B*. *h*. *leucopareia*	125,442	-	-	-	-	-	-	-
*Taverner’s Cackling Goose *B*. *h*. *taverneri*	45,770	-	-	-	-	-	-	-
*Dusky Canada Goose *B*. *canadensis occidentalis*	12,231	-	-	-	-	-	-	-
*Lesser Canada Goose *B*. *c*. *parvipes*	5,927	-	-	-	-	-	-	-
Vancouver Canada Goose *B*. *c*. *fulva*	-	-	-	-	-	-	-	-
*‡Emperor Goose *Anser canagicus*	25,408	1,988	1,186	5	3,179	0	3,179	0
Geese (unidentified)	-	406	58	0	464	0	464	0
Swans		3,908	1,535	25	5,468	64	5,532	1,150
Tundra Swan *Cygnus columbianus*	137,751	-	-	-	-	-	-	1,136
Western population	125,325	-	-	-	-	-	-	-
Eastern population	15,384	-	-	-	-	-	-	-
‡Trumpeter Swan *C*. *buccinator*	19,633	-	-	-	-	-	-	14
Lesser Sandhill Crane *Antigone canadensis*	31,393	3,700	1,406	31	5,137	1,122	6,249	716
Pacific coast population	16,140	-	-	-	-	744	-	0
Mid-continent population	15,253	-	-	-	-	368	-	716
Total	-	181,851	86,216	2,574	270,641	73,641	344,282	3,332,939

Harvest:

^(a)^ this study,

^(b)^ Harvest Information Program (HIP) [42, 43].

Alaska abundance index [43, 53–59]: Abundance indices are heterogenous among species depending on the geographic area, demographic contingents (e.g., breeding, non-breeding, hatch-year birds), and populations represented in different estimates as well as whether estimates are corrected for incomplete detection. Additional information for population data is available in S1 Dataset.

*: Species of conservation concern [60].

^‡^: Species not opened for harvest in the 2004–2015 spring-summer subsistence season at least in some management units [31].

-: Data unavailable.

**Table 2:** Annual average harvest estimates for waterfowl and Sandhill Crane and their eggs in rural Alaska by period, 1980–2015.

Species or categories	Harvest estimates (birds/year, eggs/year)									
	1980–1989^a^		1980–1995^b^		1980–1996^c^		1993–2012^d^		2004–2015^e^	
	Birds	Eggs	Birds	Eggs	Birds	Eggs	Birds	Eggs	Birds	Eggs
Ducks	210,448	-	209,454	9,294	197,575	-	-	-	151,422	12,751
Wigeons	-	-	-	-	16,415	-	-	-	8,563	211
Gadwall	-	-	-	-	576	-	-	-	639	0
Mallard	-	-	-	-	44,866	-	-	-	30,112	1,507
Northern Pintail	-	-	-	-	41,016	-	-	-	22,387	2,397
Northern Shoveler	-	-	-	-	2,881	-	-	-	3,787	298
Teals	-	-	-	-	12,563	-	-	-	8,256	461
Canvasback	-	-	-	-	1,316	-	-	-	1,801	62
Scaups	-	-	-	-	8,407	-	-	-	11,371	104
Common Eider	-	-	-	-	6,919	-	4,460	3,496	4,336	2,720
King Eider	-	-	-	-	16,469	-	16,203	925	13,906	691
Spectacled Eider	-	-	-	-	1,127	-	222	25	415	14
Steller’s Eider	-	-	-	-	438	-	230	50	202	29
Eiders (unidentified)	-	-	-	-	-	-	-	-	1,333	113
Surf Scoter	-	-	-	-	967	-	2,765	15	3,522	23
White-winged Scoter	-	-	-	-	3,506	-	7,538	47	6,669	52
Black Scoter	-	-	-	-	8,451	-	11,617	78	13,071	85
Scoters (unidentified)	-	-	-	-	4,689	-	-	-	514	0
Harlequin Duck	-	-	-	-	2,217	-	2,080	0	3,460	0
Long-tailed Duck	-	-	-	-	10,341	-	4,020	1,027	4,113	326
Bufflehead	-	-	-	-	3,916	-	3,782	62	3,475	35
Goldeneyes	-	-	-	-	6,973	-	7,252	17	6,391	73
Mergansers	-	-	-	-	1,977	-	1,556	52	1,225	106
Ducks (other, unidentified)	-	-	-	-	1,545	-	-	-	1,874	3,444
Geese	79,655	-	111,228	5,244	120,015	-	-	-	108,614	20,589
White-fronted Goose	-	-	-	-	30,950	-	-	-	41,724	6,870
Snow Goose	-	-	-	-	9,018	-	-	-	8,600	106
Brant	-	-	-	-	14,945	-	-	-	12,639	1,889
Canada/Cackling geese	-	-	-	-	61,094	-	-	-	42,008	8,200
Emperor Goose	-	-	-	-	4,008	-	-	-	3,179	521
Geese (unidentified)	-	-	-	-	-	-	-	-	464	3,003
Swans	6,894	-	10,604	1,016	9,953	-	-	-	5,468	2,093
Sandhill crane	5,283	-	8,874	787	7,200	-	-	-	5,137	1,259
Total waterfowl and Sandhill Crane	302,280	-	340,160	16,341	334,743	-	-	-	270,641	36,692
Total seabirds	-	-	19,382	115,344	36,418	-	-	-	24,315^f^	150,781^f^
Total shorebirds	-	-	741	2,741	1,411	-	-	-	2,783^g^	4,678^g^
Migratory birds (other, unidentified)	4,962	-	-	9,295	-	-	-	-	-	-
Total migratory birds	307,242	83,603	360,283	143,721	372,572	-	-	-	297,739	192,151

Sources:

^(a)^ n = 151 community-years [17];

^(b)^ n = 244 community-years [18];

^(c)^ n = 298 community-years [19];

^(d)^ n = 418 community-years [61];

^(e)^ n = 637 community-years [this study];

^(f)^ n = 545 community-years [51]; and

^(g)^ n = 775 community-years [52].

-: Data unavailable.

**Table 3:** Estimated annual average harvest of waterfowl and Sandhill Crane (birds/year) in Alaska rural regions by season, 2004–2015.

Species or categories	North Slope	Northwest Arctic	Bering Strait Mainland	St. Lawrence-Diomede Is.	Y-K Delta Coast	Y-K Delta Inland	Interior Alaska East	Interior Alaska West	Upper Copper River	Bristol Bay	Aleutian-Pribilof Is.	Kodiak Archipelago	Gulf of Alaska-Cook Inlet	Alaska rural total
Wigeons	1	1,257	265	1	1,328	1,854	1,487	457	19	705	31	502	656	8,563
Spring	1	632	172	1	254	868	834	294	0	190	2	2	0	3,250
Summer	0	62	10	0	190	246	109	4	0	25	0	2	16	664
Fall-winter	0	559	83	0	884	740	530	159	19	159	29	498	640	4,300
Unknown	0	4	0	0	0	0	14	0	0	331	0	0	0	349
Gadwall	0	0	0	0	4	0	0	0	0	55	36	532	12	639
Spring	0	0	0	0	0	0	0	0	0	0	2	3	0	5
Summer	0	0	0	0	0	0	0	0	0	0	0	0	0	0
Fall-winter	0	0	0	0	4	0	0	0	0	7	34	529	12	586
Unknown	0	0	0	0	0	0	0	0	0	48	0	0	0	48
Mallard	24	2,341	788	5	3,994	4,700	6,036	976	218	4,184	901	3,866	2,079	30,112
Spring	10	1,655	409	1	1,130	2,369	2,751	664	119	2,069	30	247	92	11,546
Summer	14	175	67	0	554	438	507	35	23	151	23	10	172	2,169
Fall-winter	0	507	312	4	2,307	1,893	2,731	277	76	1,436	847	3,533	1,815	15,738
Unknown	0	4	0	0	3	0	47	0	0	528	1	76	0	659
Northern Pintail	43	2,514	3,818	130	6,163	3,184	2,259	571	40	2,610	296	383	376	22,387
Spring	28	1,621	1,808	107	1,308	1,728	1,241	467	25	1,488	6	37	13	9,877
Summer	11	341	1,069	17	1,836	224	127	3	4	126	15	0	12	3,785
Fall-winter	4	546	941	6	3,018	1,232	866	101	11	643	275	346	351	8,340
Unknown	0	6	0	0	1	0	25	0	0	353	0	0	0	385
Northern Shoveler	0	398	350	1	960	1,022	606	80	15	282	7	1	65	3,787
Spring	0	195	240	1	154	437	340	68	11	155	0	0	2	1,603
Summer	0	71	24	0	328	121	27	0	0	7	0	0	0	578
Fall-winter	0	128	86	0	478	464	239	12	4	96	7	1	63	1,578
Unknown	0	4	0	0	0	0	0	0	0	24	0	0	0	28
Teals	11	408	363	3	922	1,212	667	236	19	1,331	892	1,317	875	8,256
Spring	11	200	215	3	146	434	303	107	9	410	68	199	9	2,114
Summer	0	28	21	0	121	179	26	3	2	117	2	0	10	509
Fall-winter	0	179	127	0	655	599	338	126	8	607	822	1,118	856	5,435
Unknown	0	1	0	0	0	0	0	0	0	197	0	0	0	198
Canvasback	0	139	23	1	59	719	618	29	14	92	23	58	26	1,801
Spring	0	85	20	1	18	539	364	22	10	42	6	0	0	1,107
Summer	0	14	1	0	13	62	74	0	3	0	0	0	0	167
Fall-winter	0	40	2	0	28	118	180	7	1	48	17	58	26	525
Unknown	0	0	0	0	0	0	0	0	0	2	0	0	0	2
Scaups	9	499	68	0	707	7,054	694	47	4	101	49	2,051	88	11,371
Spring	3	333	51	0	170	5,321	337	40	2	72	8	553	6	6,896
Summer	6	51	1	0	82	628	43	0	0	4	0	0	0	815
Fall-winter	0	100	16	0	455	1,105	314	7	2	15	41	1,498	82	3,635
Unknown	0	15	0	0	0	0	0	0	0	10	0	0	0	25
Common Eider	2,068	59	335	1,438	277	26	0	1	0	64	68	0	0	4,336
Spring	1,199	41	219	593	207	23	0	1	0	35	0	0	0	2,318
Summer	827	6	61	320	12	0	0	0	0	9	39	0	0	1,274
Fall-winter	37	12	55	525	58	3	0	0	0	20	29	0	0	739
Unknown	5	0	0	0	0	0	0	0	0	0	0	0	0	5
King Eider	6,907	29	160	522	5,019	250	0	0	0	657	349	13	0	13,906
Spring	3,110	25	156	221	4,201	236	0	0	0	621	17	1	0	8,588
Summer	3,713	3	1	130	573	0	0	0	0	0	0	0	0	4,420
Fall-winter	84	1	3	171	245	14	0	0	0	36	332	12	0	898
Unknown	0	0	0	0	0	0	0	0	0	0	0	0	0	0
Spectacled Eider	145	0	19	147	28	6	0	1	12	56	1	0	0	415
Spring	122	0	3	36	17	6	0	1	0	24	0	0	0	209
Summer	23	0	8	47	2	0	0	0	7	0	0	0	0	87
Fall-winter	0	0	8	64	9	0	0	0	5	32	1	0	0	119
Unknown	0	0	0	0	0	0	0	0	0	0	0	0	0	0
Steller’s Eider	26	38	53	15	39	1	0	0	0	12	18	0	0	202
Spring	20	0	0	6	9	1	0	0	0	11	0	0	0	47
Summer	6	0	3	5	8	0	0	0	0	0	0	0	0	22
Fall-winter	0	38	50	4	22	0	0	0	0	1	18	0	0	133
Unknown	0	0	0	0	0	0	0	0	0	0	0	0	0	0
Eiders (unidentified)	1,138	71	4	0	9	5	0	0	0	99	0	7	0	1,333
Spring	497	65	3	0	9	3	0	0	0	8	0	0	0	585
Summer	525	5	1	0	0	0	0	0	0	0	0	0	0	531
Fall-winter	116	1	0	0	0	2	0	0	0	0	0	7	0	126
Unknown	0	0	0	0	0	0	0	0	0	91	0	0	0	91
Surf Scoter	0	230	11	4	105	1,627	402	32	0	147	44	845	75	3,522
Spring	0	175	9	2	72	1,529	237	26	0	59	2	388	11	2,510
Summer	0	41	1	1	4	7	41	0	0	11	0	0	0	106
Fall-winter	0	12	1	1	29	91	115	6	0	77	42	457	64	895
Unknown	0	2	0	0	0	0	9	0	0	0	0	0	0	11
White-winged Scoter	2	134	24	12	213	2,013	3,264	25	21	76	75	761	49	6,669
Spring	1	85	24	1	167	1,666	1,821	17	7	44	1	220	10	4,064
Summer	1	6	0	3	6	34	601	0	7	0	0	0	2	660
Fall-winter	0	43	0	8	40	313	809	8	7	32	74	541	37	1,912
Unknown	0	0	0	0	0	0	33	0	0	0	0	0	0	33
Black Scoter	4	830	169	12	618	6,974	789	145	26	403	58	2,851	192	13,071
Spring	3	623	137	1	401	5,801	487	133	9	281	3	964	67	8,910
Summer	0	72	0	6	32	342	56	2	10	22	2	0	3	547
Fall-winter	1	65	32	5	185	831	246	10	7	100	53	1,887	120	3,542
Unknown	0	70	0	0	0	0	0	0	0	0	0	0	2	72
Scoters (unidentified)	0	2	0	0	0	0	384	19	0	24	8	76	1	514
Spring	0	0	0	0	0	0	320	7	0	21	0	0	0	348
Summer	0	0	0	0	0	0	0	0	0	0	0	0	0	0
Fall-winter	0	2	0	0	0	0	42	12	0	0	8	76	1	141
Unknown	0	0	0	0	0	0	22	0	0	3	0	0	0	25
Harlequin Duck	0	8	7	199	50	123	57	5	0	190	307	2,480	34	3,460
Spring	0	6	1	54	6	90	9	4	0	126	22	971	3	1,292
Summer	0	0	4	71	0	1	12	0	0	0	4	26	2	120
Fall-winter	0	2	2	74	44	32	36	1	0	64	253	1,407	28	1,943
Unknown	0	0	0	0	0	0	0	0	0	0	28	76	1	105
Long-tailed Duck	99	677	104	155	559	1,084	580	107	0	39	53	595	61	4,113
Spring	36	562	50	87	124	841	369	104	0	24	0	209	14	2,420
Summer	30	74	2	49	31	161	113	0	0	0	0	0	0	460
Fall-winter	17	35	52	19	404	82	98	3	0	15	53	386	47	1,211
Unknown	16	6	0	0	0	0	0	0	0	0	0	0	0	22
Bufflehead	0	8	36	0	32	351	863	58	6	58	98	1,832	133	3,475
Spring	0	4	35	0	13	178	454	55	3	20	0	258	6	1,026
Summer	0	4	0	0	0	33	17	0	0	0	1	0	2	57
Fall-winter	0	0	1	0	19	140	381	3	3	34	97	1,523	125	2,326
Unknown	0	0	0	0	0	0	11	0	0	4	0	51	0	66
Goldeneyes	1	73	32	5	129	1,283	983	60	26	260	201	2,873	465	6,391
Spring	0	58	22	2	51	858	472	54	8	169	0	205	69	1,968
Summer	1	8	0	0	21	71	53	0	1	12	1	5	8	181
Fall-winter	0	7	10	3	57	354	450	6	17	71	199	2,638	387	4,199
Unknown	0	0	0	0	0	0	8	0	0	8	1	25	1	43
Mergansers	3	5	3	20	232	99	113	2	3	320	101	158	166	1,225
Spring	3	0	3	2	107	72	82	2	3	181	10	10	48	523
Summer	0	4	0	5	6	1	21	0	0	9	4	0	0	50
Fall-winter	0	1	0	13	119	26	10	0	0	119	86	148	118	640
Unknown	0	0	0	0	0	0	0	0	0	11	1	0	0	12
Ducks (other, unidentified)	127	494	61	2	239	125	163	97	36	301	55	104	70	1,874
Spring	72	349	13	0	84	76	69	49	14	149	11	0	3	889
Summer	36	7	9	0	47	6	7	0	7	62	5	0	5	191
Fall-winter	19	137	39	2	108	43	87	48	15	80	39	28	62	707
Unknown	0	1	0	0	0	0	0	0	0	10	0	76	0	87
Ducks, total	10,608	10,214	6,693	2,672	21,686	33,712	19,965	2,948	459	12,066	3,671	21,305	5,423	151,422
Spring	5,116	6,714	3,590	1,119	8,648	23,076	10,490	2,115	220	6,199	188	4,267	353	72,095
Summer	5,193	972	1,283	654	3,866	2,554	1,834	47	64	555	96	43	232	17,393
Fall-winter	278	2,415	1,820	899	9,168	8,082	7,472	786	175	3,692	3,356	16,691	4,834	59,668
Unknown	21	113	0	0	4	0	169	0	0	1,620	31	304	4	2,266
White-fronted Goose	11,556	2,203	805	18	12,413	9,072	3,608	825	1	1,199	0	0	24	41,724
Spring	11,056	1,751	669	12	8,103	7,073	3,120	776	0	943	0	0	0	33,503
Summer	447	67	28	0	1,733	395	299	4	0	20	0	0	13	3,006
Fall-winter	49	385	108	6	2,577	1,604	181	45	1	98	0	0	11	5,065
Unknown	4	0	0	0	0	0	8	0	0	138	0	0	0	150
Snow Goose	521	434	3,849	986	1,664	702	396	27	9	2	1	0	9	8,600
Spring	493	430	3,564	123	1,202	594	336	21	6	0	0	0	0	6,769
Summer	23	0	69	69	5	16	46	3	0	0	0	0	0	231
Fall-winter	5	4	216	794	457	92	14	3	3	2	1	0	9	1,600
Unknown	0	0	0	0	0	0	0	0	0	0	0	0	0	0
Brant	1,407	829	2,452	373	5,582	320	66	30	2	558	1,004	15	1	12,639
Spring	1,045	677	1,984	141	4,267	269	62	28	1	442	208	0	0	9,124
Summer	313	120	116	60	389	29	0	0	0	27	9	0	0	1,063
Fall-winter	49	32	352	172	926	22	4	2	1	83	787	15	1	2,446
Unknown	0	0	0	0	0	0	0	0	0	6	0	0	0	6
Canada/Cackling geese	572	4,121	4,374	74	15,073	10,094	2,365	1,273	26	2,821	657	325	233	42,008
Spring	512	3,220	2,508	33	6,262	7,459	1,679	1,104	20	1,699	23	90	12	24,621
Summer	47	235	225	19	2,536	471	263	10	0	183	42	0	13	4,044
Fall-winter	13	661	1,641	22	6,275	2,164	418	159	6	858	592	235	208	13,252
Unknown	0	5	0	0	0	0	5	0	0	81	0	0	0	91
Emperor Goose	1	55	134	794	1,285	178	0	2	0	169	536	25	0	3,179
Spring	1	55	112	203	907	101	0	0	0	70	76	0	0	1,525
Summer	0	0	9	168	235	40	0	0	0	1	10	0	0	463
Fall-winter	0	0	13	423	143	37	0	2	0	98	445	25	0	1,186
Unknown	0	0	0	0	0	0	0	0	0	0	5	0	0	5
Geese (unidentified)	182	97	0	0	0	24	5	39	1	89	14	2	11	464
Spring	182	95	0	0	0	18	2	25	0	79	0	0	2	403
Summer	0	1	0	0	0	0	2	0	0	0	0	0	0	3
Fall-winter	0	1	0	0	0	6	1	14	1	10	14	2	9	58
Unknown	0	0	0	0	0	0	0	0	0	0	0	0	0	0
Geese, total	14,239	7,739	11,614	2,245	36,017	20,390	6,440	2,196	39	4,838	2,212	367	278	108,614
Spring	13,289	6,228	8,837	512	20,741	15,514	5,199	1,954	27	3,233	307	90	14	75,945
Summer	830	423	447	316	4,898	951	610	17	0	231	61	0	26	8,810
Fall-winter	116	1,083	2,330	1,417	10,378	3,925	618	225	12	1,149	1,839	277	238	23,607
Unknown	4	5	0	0	0	0	13	0	0	225	5	0	0	252
Swans	32	99	422	71	1,867	2,650	72	15	8	231	0	0	1	5,468
Spring	30	47	260	33	976	1,928	42	14	5	154	0	0	0	3,489
Summer	1	5	47	21	219	123	0	0	0	3	0	0	0	419
Fall-winter	1	46	114	17	672	599	28	1	3	53	0	0	1	1,535
Unknown	0	1	1	0	0	0	2	0	0	21	0	0	0	25
Sandhill Crane	13	77	1,146	108	2,329	1,011	136	39	0	229	4	0	45	5,137
Spring	9	56	562	42	1,674	735	66	31	0	120	0	0	0	3,295
Summer	4	3	40	51	157	75	5	5	0	31	3	0	31	405
Fall-winter	0	18	544	15	498	201	60	3	0	52	1	0	14	1,406
Unknown	0	0	0	0	0	0	5	0	0	26	0	0	0	31

**Fig 3:**
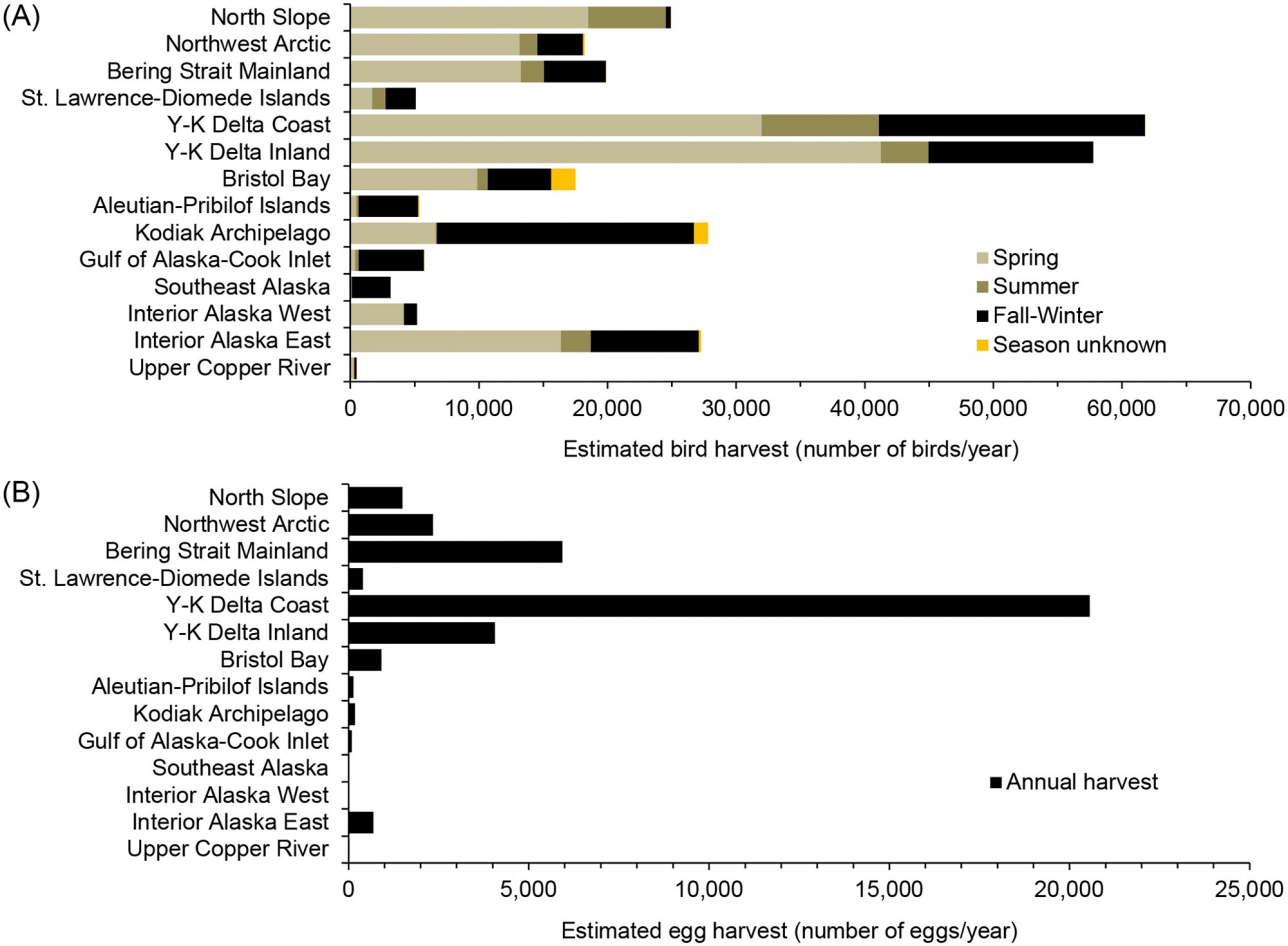
Regional harvest amounts of waterfowl and Sandhill Crane (A) and their eggs (B) by rural hunters in Alaska, annual average 2004–2015.

Comparing amounts of total waterfowl harvest among regions, relatively high harvest (about 60,000 birds/year) occurred in the Y-K Delta Coast (23% of the total waterfowl harvest) and Y-K Delta Inland (21%) (Fig 3A). Intermediary harvest (about 15,000–30,000 birds/year or 6%–10%) occurred in Interior Alaska East, Kodiak Archipelago, Bristol Bay, Bering Strait Mainland, North Slope, and Northwest Arctic. The remaining regions had a relatively low harvest (up to about 6,000 birds/year or 2%; Gulf of Alaska-Cook Inlet, Aleutian-Pribilof Islands, St. Lawrence-Diomede Islands, Interior Alaska West, and Upper Copper River).

The estimated harvest of waterfowl eggs in rural regions was 36,692 eggs/year (Table 4, S6 Table in S1 File). Species/categories that accounted for at least 5% of the waterfowl egg harvest included Canada/Cackling geese (22% of the total egg harvest), White-fronted Goose (19%), ducks (unidentified) (9%), geese (unidentified) (8%), Common Eider *Somateria mollissima* (7%), Northern Pintail (7%), swans (6%), and Brant (5%) (Table 2, Fig 2B). The regions accounting for most of the waterfowl egg harvest were Y-K Delta Coast (56%), Bering Strait Mainland (16%), and Y-K Delta Inland (11%) (Fig 3B).

**Table 4:** Estimated annual average harvest of waterfowl and Sandhill Crane eggs (eggs/year) in Alaska rural regions, 2004–2015.

Species or categories	North Slope	Northwest Arctic	Bering Strait Mainland	St. Lawrence-Diomede Is.	Y-K Delta Coast	Y-K Delta Inland	Interior Alaska East	Interior Alaska West	Upper Copper River	Bristol Bay	Aleutian-Pribilof Is.	Kodiak Archipelago	Gulf of Alaska-Cook Inlet	Alaska rural total
Wigeons	0	12	13	0	77	78	23	0	0	8	0	0	0	211
Gadwall	0	0	0	0	0	0	0	0	0	0	0	0	0	0
Mallard	3	97	209	1	449	441	104	0	3	188	0	8	4	1,507
Northern Pintail	7	226	525	5	1,062	388	66	4	5	109	0	0	0	2,397
Northern Shoveler	0	8	7	0	190	30	61	0	2	0	0	0	0	298
Teals	0	0	66	0	233	125	26	0	2	9	0	0	0	461
Canvasback	0	0	5	0	11	5	41	0	0	0	0	0	0	62
Scaups	0	12	31	0	22	35	0	0	0	4	0	0	0	104
Common Eider	108	12	2,311	205	50	0	0	0	0	0	34	0	0	2,720
King Eider	68	41	467	77	27	0	0	0	0	3	8	0	0	691
Spectacled Eider	0	0	12	2	0	0	0	0	0	0	0	0	0	14
Steller’s Eider	0	0	17	4	8	0	0	0	0	0	0	0	0	29
Eiders (unidentified)	48	2	57	0	0	0	0	0	0	0	2	4	0	113
Surf scoter	0	0	0	0	15	0	1	0	0	0	0	7	0	23
White-winged Scoter	8	0	4	0	12	0	18	0	0	0	0	10	0	52
Black Scoter	5	0	11	0	14	5	18	0	0	0	0	32	0	85
Scoters (unidentified)	0	0	0	0	0	0	0	0	0	0	0	0	0	0
Harlequin Duck	0	0	0	0	0	0	0	0	0	0	0	0	0	0
Long-tailed Duck	8	0	228	41	38	9	2	0	0	0	0	0	0	326
Bufflehead	0	0	6	0	4	0	0	0	1	0	0	24	0	35
Goldeneyes	0	0	5	4	12	0	0	0	0	0	0	52	0	73
Mergansers	1	0	52	15	0	5	0	0	0	7	11	15	0	106
Ducks (other, unidentified)	190	321	217	0	1,355	749	62	0	0	421	69	25	35	3,444
Ducks, total	446	731	4,243	354	3,579	1,870	422	4	13	749	124	177	39	12,751
White-fronted Goose	263	20	24	7	6,222	318	8	0	0	8	0	0	0	6,870
Snow Goose	10	0	36	0	34	26	0	0	0	0	0	0	0	106
Brant	30	52	220	9	1,568	10	0	0	0	0	0	0	0	1,889
Canada/Cackling geese	35	812	592	10	5,918	668	67	0	0	73	0	0	25	8,200
Emperor Goose	0	0	68	3	437	12	0	0	0	1	0	0	0	521
Geese (unidentified)	601	416	168	0	1,029	565	123	0	0	55	0	15	31	3,003
Geese, total	939	1,300	1,108	29	15,208	1,599	198	0	0	137	0	15	56	20,589
Swans	81	111	349	7	1,014	487	15	0	0	29	0	0	0	2,093
Sandhill Crane	11	83	234	6	758	107	57	0	0	3	0	0	0	1,259

### Geographic and seasonal harvest patterns

**Fig 4:**
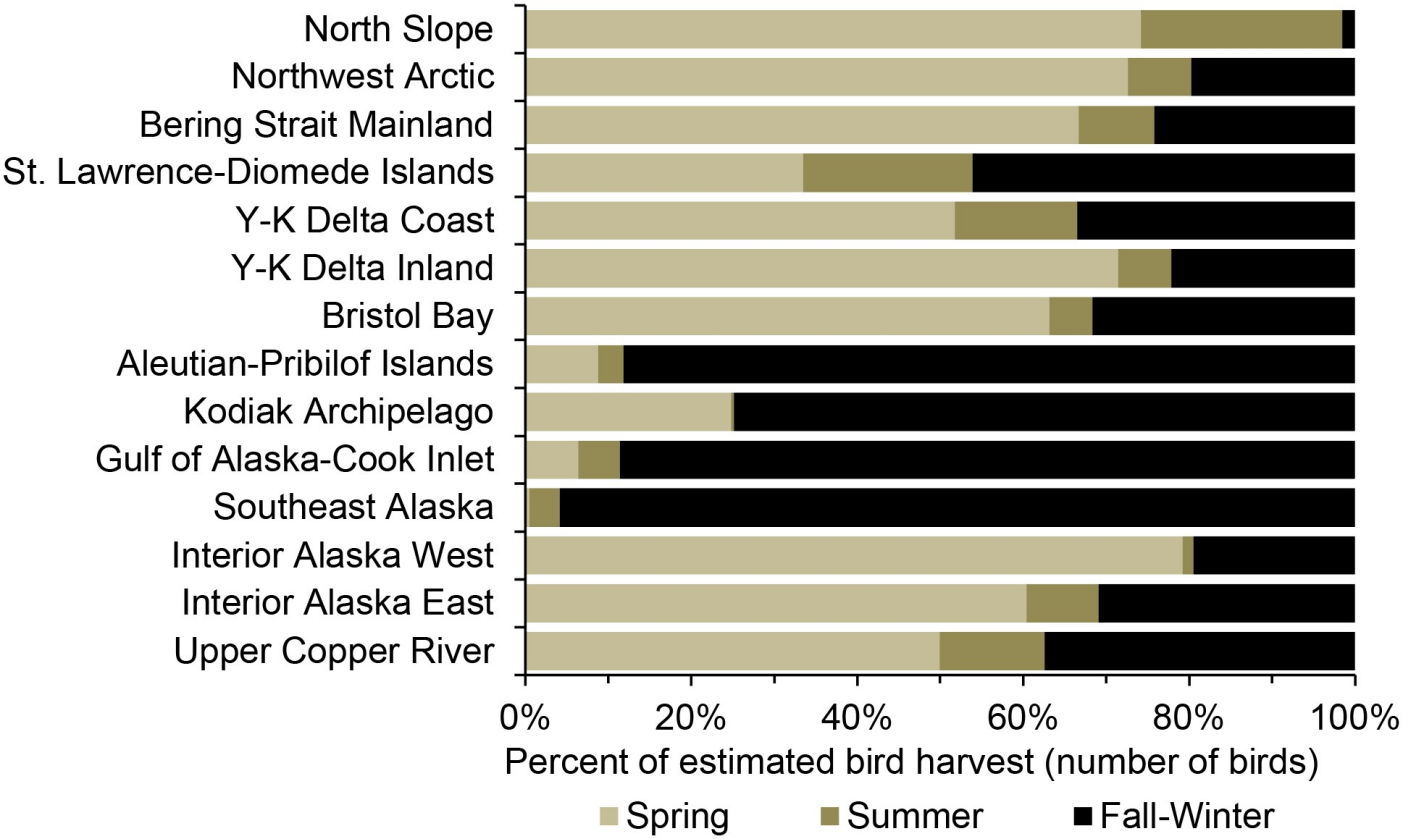
Regional seasonality of the harvest of waterfowl and Sandhill Crane by rural hunters in Alaska, annual average 2004–2015.

Pacific-Aleutian Mainland and Islands—Waterfowl harvest by hunters residing in the mostly maritime Gulf of Alaska-Cook Inlet, Kodiak Archipelago, and Aleutian-Pribilof Islands regions had a predominant fall-winter component (at least 79% of the annual total harvest) and high diversity of dabbling and sea ducks (Fig 4, S7 Fig in S1 File). Elements typical of individual regions in this group included a relatively high harvest of scaups on the Kodiak Archipelago and geese on the Aleutian-Pribilof Islands (Brant, Canada/Cackling geese, and Emperor Goose). Total waterfowl harvest amounts were low to intermediary in these regions.

Bering Sea Mainland—Waterfowl harvests in the Bristol Bay, Y-K Delta Coast and Inland, Bering Strait Mainland, and Northwest Arctic regions had a strong spring component, a high species diversity (including coastal species such as eiders, Brant, Emperor Goose), and relatively high harvests of swans and Sandhill Crane (Fig 4, S8A–S8E Fig in S1 File). Substantial fall harvests also occurred in these regions for some species (e.g., Mallard, White-fronted Goose, Canada/Cackling geese). Total waterfowl harvests in these regions were intermediate to high reflecting their extensive wetland ecosystems.

St. Lawrence-Diomede Islands—Waterfowl harvests by hunters residing on the pelagic islands of the Bering Sea were relatively low and presented a distinct seasonal pattern and species composition (Fig 4, S8F Fig in S1 File). This harvest had relatively strong summer (20%) and fall-winter (46%) components and a low species diversity, primarily represented by eiders (mostly reported as Common Eider), Harlequin Duck *Histrionicus histrionicus*, Long-tailed Duck *Clangula hyemalis*, Brant, Snow Goose *Anser caerulescens*, and Emperor Goose.

North Slope—Waterfowl harvest by hunters residing in this high Arctic region were intermediary, mostly lacked a fall-winter component, and had a low species diversity being primarily composed of eiders (mostly reported as King Eider) and geese (especially White-fronted Goose) (Fig 4, S9 Fig in S1 File).

Interior Alaska-Upper Copper River—Waterfowl harvest in the landlocked Interior Alaska (West and East) and Upper Copper River had a strong spring component (similar to other Alaska regions), but lacked coastal species such as eiders, Brant, and Emperor Goose (Fig 4, S10 Fig in S1 File). Total waterfowl harvest amounts were low to intermediary in these regions.

### Harvests in Alaska by rural residents and other users

**Fig 5:**
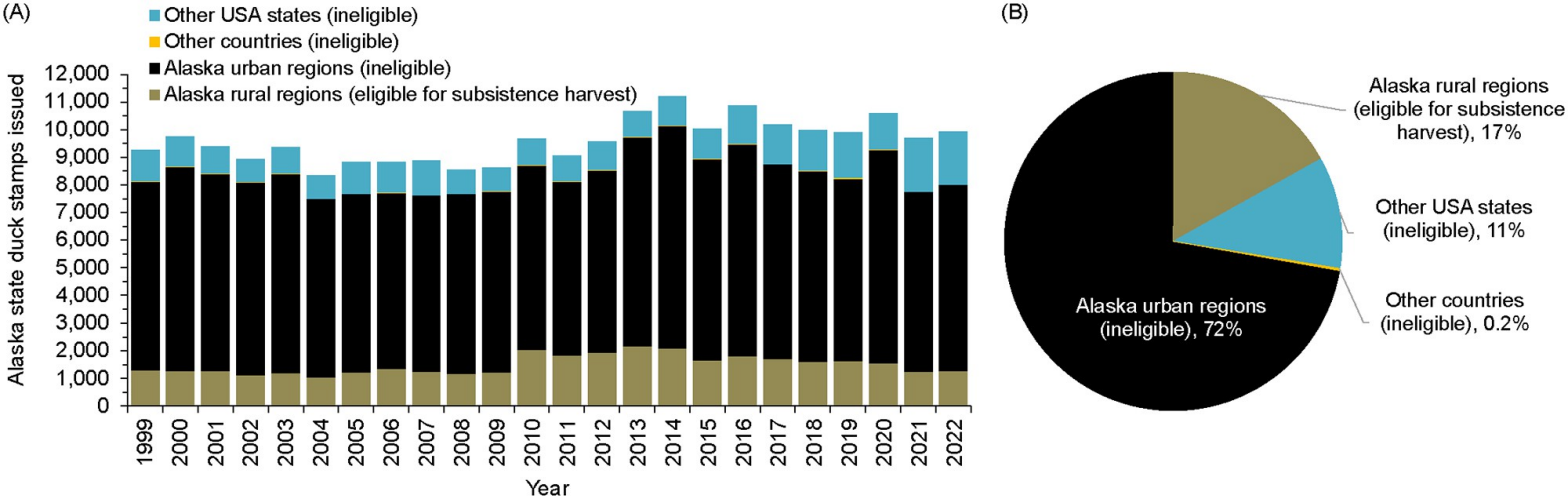
Alaska state duck stamps issued by region of residence of stamp holders. (A) 1999–2022 Annual values; (B) annual average for the 2004–2015 reference period in this study. Rural regions were defined in this study as those eligible to participate in the Alaska spring-summer subsistence harvest of migratory birds. Source: Alaska Department of Fish and Game-Division of Administrative Services unpublished data.

Residents of Alaska rural regions accounted for 17% of the annual average number of Alaska duck stamps issued (2004–2015; Fig 5, S11 Table in S1 File). Fall-winter harvest by rural residents (86,216 birds/year; this study) was higher than harvests estimated from the HIP survey (73,641 birds/year) [42, 43], which included residents of Alaska urban and rural regions, other U.S. states, and other countries who purchased an Alaska duck stamp (Table 1). For lack of better options, we assumed the HIP survey represented harvest primarily by users other than Alaska rural residents (hereinafter “other users”). An unknown level of overlap exists in fall-winter harvests represented in the HIP survey and in this study. However, results from these two datasets suggest substantial differences in the populations sampled by household surveys in rural regions and by the HIP survey. First, the composition of species in fall-winter harvests estimated from the HIP survey included higher proportions of dabbling ducks than fall-winter harvest estimates from this study for rural regions (S12 Fig in S1 File). Second, participation of rural residents in the HIP program by region often was incommensurate with levels of fall-winter harvest (S13 and S14 Figs in S1 File). For example, the Y-K Delta Coast and Inland regions accounted for 12% of annual average number of duck stamps issued to Alaska rural residents, but 39% of the fall-winter rural harvest. In contrast, the Kodiak Archipelago, Bristol Bay, Gulf of Alaska-Cook Inlet, and Bering Sea Mainland regions accounted for 62% of the annual average number of ducks stamps issued to rural residents, but 37% of the fall-winter rural harvest.

Spring-summer and fall-winter harvests by rural hunters represented at least 79% of the total harvest of waterfowl and Sandhill Crane in Alaska, including fall-winter harvest by other users (Table 1). As a proportion of rural residents participate in the HIP survey, 21% is likely an overestimate of the contribution of other users to the total harvest in Alaska. In addition, the total number of birds harvested in Alaska is likely smaller than the sum of estimates obtained from household surveys in rural communities and the HIP survey.

**Fig 6:**
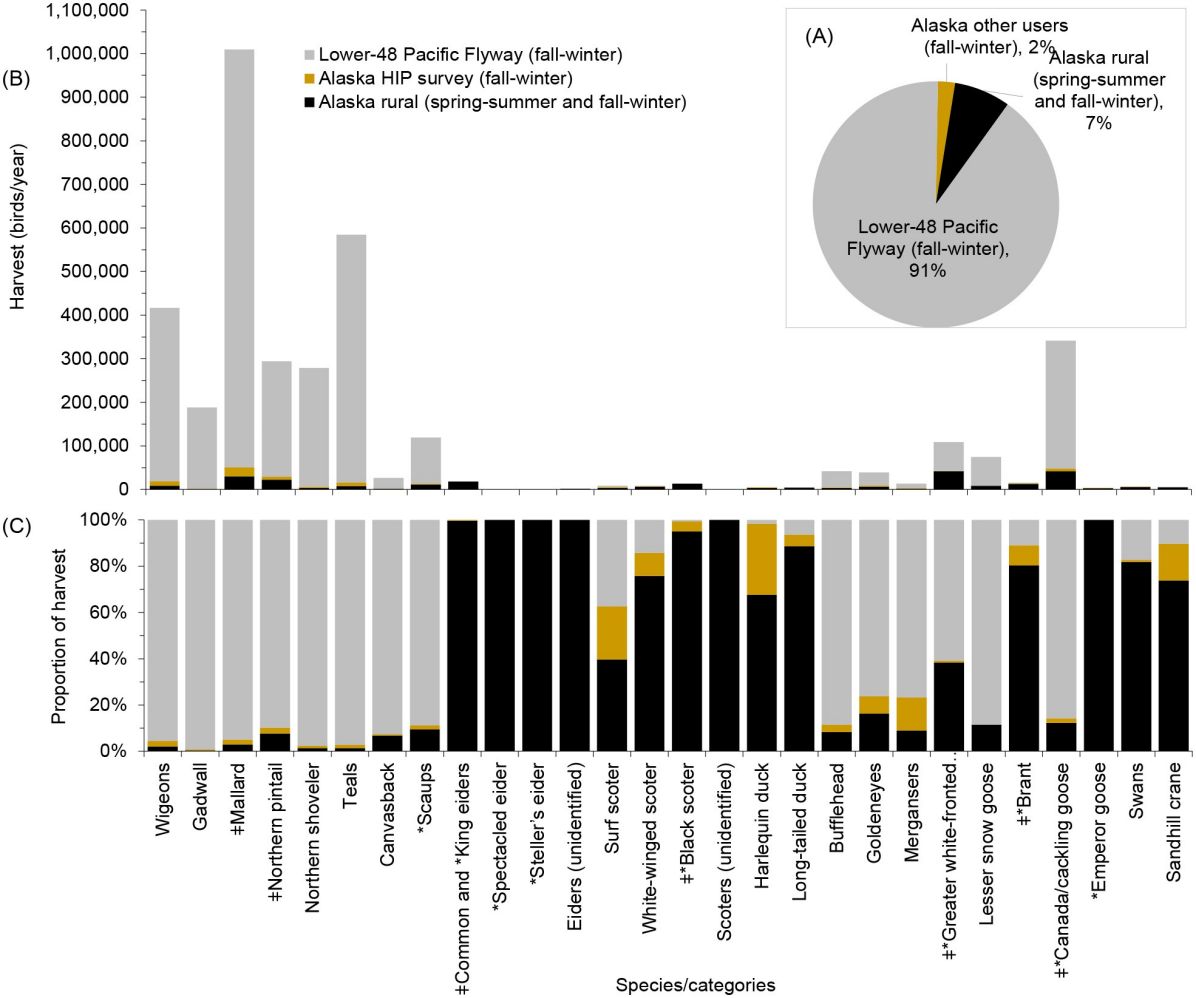
Annual waterfowl and Sandhill Crane harvest in the Pacific Flyway by user groups, 2004–2015 annual average. (A) Proportion of the total harvest, species combined. (B) Amount of harvest (birds/year) by species. (C) Proportion of harvest by species. *: Species or populations of conservation concern. ǂ: Species/categories that represented at least 5% of the harvest in rural regions in Alaska. Sources: harvest data for rural regions in Alaska [this study]; fall-winter harvest data for other users [42, 43].

Harvests by rural hunters in Alaska represented 7% of the total waterfowl harvest in the Pacific Flyway (species combined); this percentage was largely defined by a high contribution of dabbling ducks to the harvest in the Lower-48 part of the Pacific Flyway (Table 1, Fig 6A). Rural hunters in Alaska accounted for most of the total Pacific Flyway harvests for the four eider species, two scoter species, Harlequin Duck, Long-tailed Duck, Brant, Emperor Goose, swans, and Sandhill Crane (Fig 6B and 6C).

## Discussion

### Management implications

Portraying rural harvests in relation to other harvests in Alaska and the Pacific Flyway can facilitate collaboration for harvest management and bird conservation across Alaska’s regions and flyways by helping users to understand their contributions to the total harvest. This study can also help to inform regulatory decisions to achieve collaboratively defined management goals, support sustainable harvest opportunities, and stabilize or reverse bird population declines. For example, collaborative actions to temporarily reduce some spring harvest may support stable Emperor Goose numbers [62].

The seasonality of harvests and species composition in rural Alaska are significant from a harvest management perspective. The legally-allowed spring-summer harvest of migratory birds is unique to Alaska and Canada. This exception intended to provide for food and socio-cultural needs of northern Indigenous people. Sixty-eight percent of harvests in rural Alaska occurred during spring-summer (pre-breeding and nesting) thus including a higher proportion of breeding-age birds than harvests during fall-winter. Adult birds have higher annual survival and reproductive value for populations than immature birds [63–66]. Nevertheless, healthy bird populations have withstood some levels of spring-summer harvest, which has occurred in Alaska for millennia without known widespread declines of bird populations across species. Indigenous users often have different values related to spring-summer bird harvesting than stakeholders with science training or a sport hunting perspective. For example, Indigenous users are more comfortable with effects of spring-summer harvesting on bird populations than other stakeholders [62]. In addition, Indigenous users often do not identify with principles and tools commonly used in science-based harvest management that evolved within Euro-American contexts [67]. While recognizing different values, collaboration in harvest management must remain attentive and responsive to indications of weakening bird populations.

Waterfowl harvests in rural Alaska included species of conservation concern and populations that are closely regulated or closed to harvest during the fall-winter season (Table 1, Fig 6C). It is often challenging to develop effective management strategies and to evaluate the impact of harvests on bird populations because of uncertainty and biases in assessments of both harvest and populations [68]. Most data on the abundance of waterfowl in Alaska refer to incomplete assessments (indices) of the number of birds breeding or occurring in Alaska (Table 1, S1 Dataset). Harvests in Alaska include birds breeding elsewhere. Most Common and King eiders harvested in Alaska are likely Canada-breeding birds not included in population surveys conducted in Alaska. Spectacled Eiders counted on breeding grounds in Alaska are a small part of their total number that occur in Alaska, including Russia-breeding birds that winter in the Bering Sea south of Alaska’s St. Lawrence Island. Most (>95%) Steller’s Eiders that occur in Alaska in spring, fall, and winter are part of the Pacific population that breeds in Russia. Most waterfowl abundance data for Alaska do not allow to estimate harvest rates (Table 1, S1 Dataset).

Efforts continue to improve bird population and harvest data. But it is challenging and expensive to quantify bird populations and harvests over large geographic areas that adequately encompass relevant population units and socio-ecological processes [69–71]. These challenges highlight the need for harvest management approaches that have more flexible information needs, that are more inclusive of diverse ways of knowing (specifically local and Indigenous knowledge), and that are more conducive to build trust and collaboration among researchers, managers, and diverse resource users. It may be beneficial to reassess expectations about the kinds of data and levels of precision that could be reasonably achieved in the foreseeable future. Trust and collaboration are important to learn how to work with different kinds of information available while accounting for their limitations. This study highlights the need for continuous collaboration, outreach, and education about species of conservation concern and those closed to harvest.

We estimated Emperor Goose harvest during the period when its harvest was closed (3,179 birds/year, Table 3) [46–48]. The 1987–2016 harvest closure was unable to completely suppress subsistence harvest, but it likely reduced harvests. It is also possible that some harvesters were unwilling to report illegally taken birds, so harvest estimates for the closure period may be biased low. Re-opening harvest may have resulted in increased take as well as harvest reporting rates. Harvest estimates available for some regions in 2017–2020 after the hunt was re-opened amounted to an average 6,251 birds/year (S15 Table, S16 Fig in S1 File) [67, 72]. While acknowledging that harvest estimates for Emperor Goose often have wide confidence intervals, these estimates have shown consistent patterns for regions and seasons. The Y-K Delta and Bering Strait-Norton Sound regions accounted for 87% of the annual Emperor Goose harvest during 2017–2020, thus collaboration with residents of these regions plays a key role in harvest management.

### Geographic and seasonal harvest patterns

Patterns of waterfowl and Sandhill Crane harvest by rural Alaska residents quantified in this study aligned with and complemented the earlier qualitative description [17]. Waterfowl hunting was highest in spring and largely decreased during summer in most rural regions (Fig 5). In summer, subsistence and commercial fishing (especially for salmon) are main activities in many rural regions [25]. Also, traditional customs and current harvest regulations curtail bird harvest during nesting and chick rearing so birds can breed and multiply.

Geographic and seasonal patterns of harvests in this study reflected the availability of bird species during their annual migration cycle and the distribution of habitats favorable to waterfowl. In the Bering Sea mainland regions (Bristol Bay, Y-K Delta Coast and Inland, Bering Strait Mainland, and Northwest Arctic), a strong spring harvest component, but also fall harvests, reflected high bird abundance on primary breeding grounds as well as southward migration from northern regions. Waterfowl harvests in the southern coastal regions (Aleutian-Pribilof Islands, Kodiak Archipelago, and Gulf of Alaska-Cook Inlet) were characterized by a strong fall-winter component as several species of waterfowl migrate through (dabbling ducks and geese) and overwinter (e.g., scaups, goldeneyes, Brant, Emperor Goose, and scoters) in these regions. In the North Slope, birds out-migrate after a brief breeding season and are mostly unavailable for harvest in fall-winter. Bird harvesting in this region happened primarily in spring, but also in late summer during the post-breeding migration. In the St. Lawrence-Diomede Islands region, waterfowl harvests were about evenly distributed between spring-summer and fall-winter reflecting various migration patterns between eastern and western Beringia for breeding and molting in spring, summer, and fall.

In several regions, spring was the largest component of the annual waterfowl harvest—Y-K Delta, Interior Alaska, Upper Copper River, Northwest Arctic, and Bering Strait Mainland. Diverse factors likely play a role in defining this pattern. These regions are important bird breeding grounds [3]. For people in rural Alaska, arriving migratory birds are often the first source of fresh food after a long winter. Spring is also a preferred season to harvest some waterfowl species (e.g., Emperor Goose) because they are fat and are considered tastier at this time of the year, or because they are not molting body feathers (embedded pin feathers interfere with consumption of the skin) [73]. Also, harvest activities during fall are often focused on other resources such as moose, caribou, berries, and fish.

### Harvest amounts over the decades

**Fig 7:**
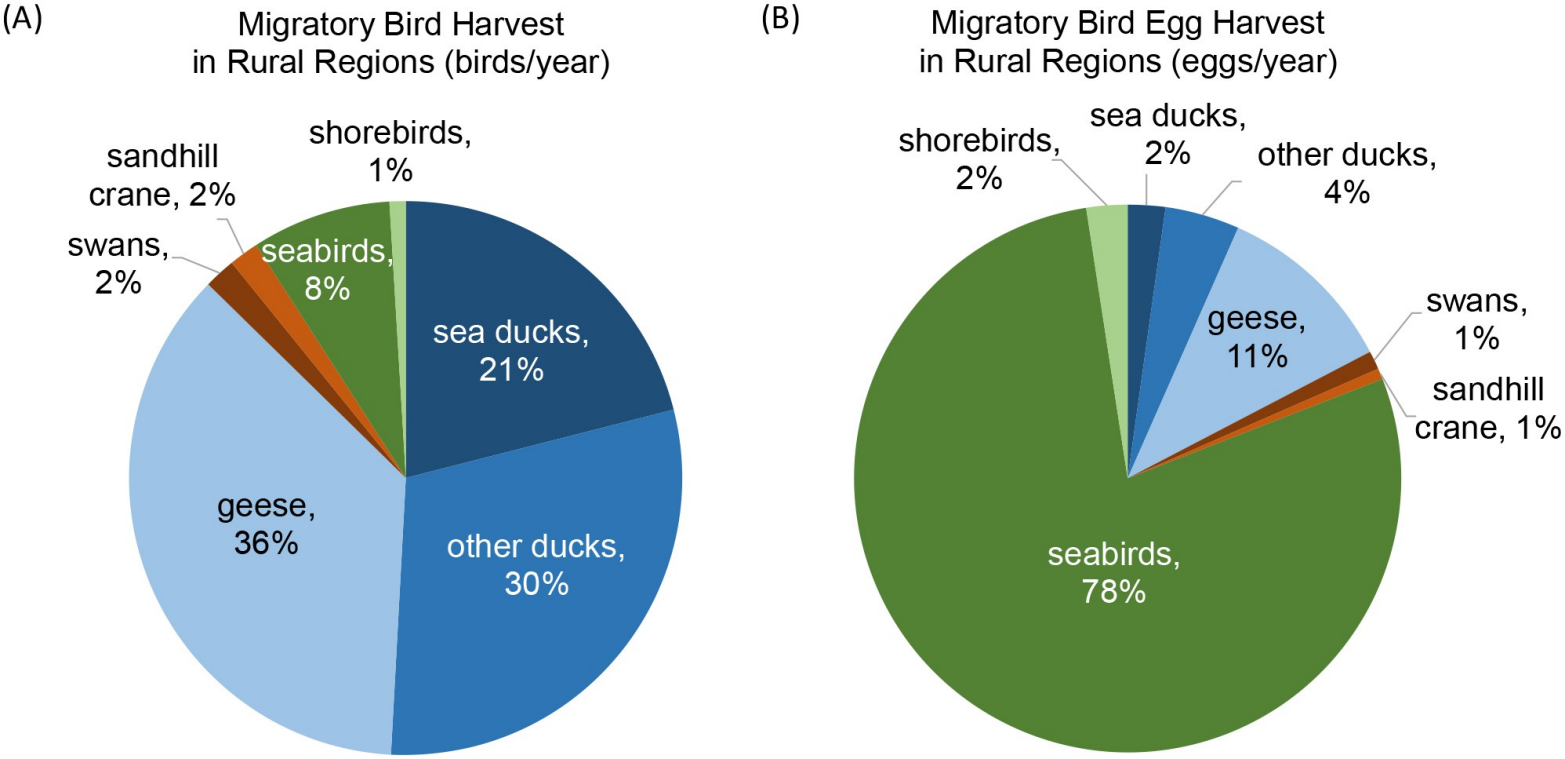
Harvest of migratory birds (A) and their eggs (B) in rural regions in Alaska, annual average 2004–2015. Sources: waterfowl and Sandhill Crane [this study], seabirds [51], and shorebirds [52].

In recent decades, waterfowl and Sandhill Crane (this study) represented 91% of the annual average number of migratory birds harvested in rural Alaska including seabirds (8%) and shorebirds (1%) (Table 2, Fig 7A) [51, 52]. Waterfowl eggs represented 19% of the total number of eggs of migratory birds harvested in rural Alaska including eggs of seabirds (78%) and shorebirds (2%) (Fig 7B). A higher contribution of eggs of seabirds and geese to the total egg harvest is related to colonial breeding in some of these species, as the concentration of resources in time and space usually increases harvest efficiency.

It is unclear if some differences in harvest estimates over the decades were due to changes in harvest amounts or differences in datasets (Table 2). Harvest estimates for 1980–1989 [17] were based on a substantially smaller dataset than subsequent studies (e.g., the Aleutian-Pribilof and St. Lawrence-Diomede islands were not represented and harvest data by seasons and species were largely unavailable). Thus, we focused comparisons between results from this study (2004–2015) with harvest estimates for 1980–1996 [19], which included estimates at the species/category level.

Harvest estimates for geese in this study were comparable to those previously available for 1980–1996 [19]. Harvest estimates for ducks were 23% lower in this study, but it was difficult to parse differences at the species level. For example, quantifying the harvest of eiders and scoters at the species level was challenging because of species identification issues (especially females) and frequent use of multi-species categories in harvest surveys. Some duck species/categories that showed the most reduced harvests (30%–50%) included wigeons, Northern Pintail, and Long-tailed Duck, which are species harvested in larger numbers as compared to other ducks. Harvest estimates in this study also were 45% lower for swans and 29% lower for Sandhill Crane than in the previous estimates. Similarly to waterfowl and Sandhill Crane, evidence of reduced seabird harvest in rural regions of Alaska in recent decades also supports some reduction in the overall bird harvest [51].

Estimates of the harvest of sea ducks and their eggs in rural Alaska were available for the 1993–2012 period [61]. We updated harvest estimates for sea ducks to represent a more recent period and included additional data for the Aleutian-Pribilof Islands and Kodiak Archipelago regions. The updated harvest estimates for sea ducks were similar to (within 1%) those previously available but estimates for sea duck eggs harvest were 36% lower (Table 2). This difference was largely due to lower harvest estimates for eggs of Common Eider and Long-tailed Duck, which were primarily harvested in the Bering Strait Mainland region (Table 4). It is unclear if this difference was due to socio-ecological factors or challenges in harvest assessment. We refer to Rothe et al. (2015) [61] for discussion of harvest patterns and management topics pertaining to sea ducks.

The amendments to migratory bird treaties ratified in 1997, which legally authorized the spring-summer harvest in Alaska, stipulated that subsistence harvests should remain at traditional levels relative to bird population sizes [2]. This stipulation resulted from concerns during the amendment process that the legal authorization of the spring-summer harvest and potential human population growth in rural Alaska could result in greatly increased harvests [74]. This study representing the 2004–2015 reference period support that bird harvests did not increase in rural Alaska after regulations allowing the spring-summer subsistence harvest of migratory birds were first implemented in 2003 (Table 2) (see also [75]). Considering increases in waterfowl populations (especially geese) in recent decades, it is possible that harvests in rural Alaska decreased proportionally to at least some bird populations [76].

### Harvest assessment

Differences in the amounts and species composition of fall-winter harvests for rural regions (this study) as compared with estimates from the HIP survey suggest that HIP underestimated fall-winter harvests in Alaska. Indigenous hunters in rural Alaska tend to show low participation in agency-led harvest management tools such as hunting licenses, stamps, permits, mandatory harvest reporting, and reporting of banded birds taken [77, 78]. For example, mandatory reporting for the Emperor Goose and Tundra Swan *Cygnus columbianus* fall-winter harvest permits has been ineffective to quantify rural harvests [67]. Alaska Native leaders have opposed the requirements for state and federal duck stamps and obtained an exemption from the federal duck stamp requirement [2]. Overcoming these challenges will take long-term collaboration with hunters. This study highlights the need for an assessment of the representation of diverse user groups in the HIP harvest estimates for Alaska and of options for making HIP data more applicable for harvest management, perhaps as providing separate harvest estimates for residents of Alaska rural regions and other users.

Subsistence harvests can show large annual variation in their amounts and composition related to socio-economic and ecological factors [79]. Overall, spring accounted for 58% of the total waterfowl harvest in rural Alaska. Annual variation in spring breakup pace and conditions can determine bird hunting opportunity. Travel becomes difficult and hazardous as ice and snow deteriorate [17]. Annual variation in harvest amounts and composition makes it challenging to depict the full range of harvests and to detect temporal changes. This study benefited from a large dataset accumulated over decades with broad geographic coverage and multiple years of data for individual communities. Notably, such large datasets are often unavailable for harvest assessment and are unlikely to become available due to limited resources [61, 71, 80]. Understanding socio-ecological factors that affect intrinsic characteristics of the data as well as harvest patterns is important to improve collaboration among stakeholders regarding harvest assessment and to optimize uses of the available data, even if the data are imperfect [69, 70]. Future harvest assessment efforts could also benefit from emerging analytical approaches to accommodate uncertainty and variability in harvest data [81].

Harvest data historically have been and will continue to be key to ensure a meaningful role for Alaska Native people in migratory bird harvest management and conservation, to ensure that harvests remain sustainable, and to address allocation issues [2, 27]. The data used in this study representing the 2004–2015 period were already retrospective because of limited availability of and incompatibility with more recent data. The AMBCC annual harvest survey—the main data source used in this study—was not conducted after 2019 due to insufficient funds and disagreement over survey goals. Because of large variation in annual harvest estimates, an updated bird harvest assessment for rural Alaska would require multiple years of data representing the diverse regions. This study highlights the importance of harvest monitoring to inform management and conservation.

## Supporting information

S1 File Tables, figures, and appendix. (PDF)

S2 File Stovall (2000) bird harvest survey, Kodiak 1999. (PDF)

S3 File Webb (2000) bird harvest survey, Koyukuk-Nowitna 1998–1999. (PDF)

S1 Dataset Waterfowl population data, Alaska 2004–2015. (XLSX)
