# Supplementary material for: Harvest of waterfowl and Sandhill Crane in rural Alaska: Geographic and seasonal patterns
Source: PLoS One. 2024 Jul 25;19(7):e0307135. doi: 10.1371/journal.pone.0307135 (PMC11271962; doi:10.1371/journal.pone.0307135)
Supplement: S1 File — (PDF) [file pone.0307135.s001.pdf]

Supporting Information
Harvest of waterfowl and Sandhill Crane in rural Alaska:
Geographic and seasonal patterns

Naves LC, Schamber JL (2024) PLOS ONE

Contents

S1 Table. Dataset (community-years) used to estimate harvest of waterfowl and Sandhill Crane in rural Alaska representing the 2004–2015 reference period. 2

S2 Figure. Temporal distribution of the data used to estimate harvest of waterfowl and Sandhill Crane by rural hunters in Alaska representing the 2004–2015 reference period. 4

S3 Appendix. Formulas used to estimate harvest and confidence interval. 5

S4 Table. Confidence intervals (as percentage of the estimate) for harvest estimates for waterfowl and Sandhill Crane, 2004–2015. 7

S5 Figure. Seasonality of the harvest of waterfowl and Sandhill Crane by rural hunters in Alaska, annual average 2004–2015. 14

S6 Table. Confidence intervals (as percentage of the estimate) for harvest estimates for eggs of waterfowl and Sandhill Crane, 2004–2015. 15

S7 Figure. Waterfowl and Sandhill Crane harvest by residents of the Pacific-Aleutian mainland and islands regions, 2004–2015 annual average. 17

S8 Figure. Waterfowl and Sandhill Crane harvest by residents of the Bering Sea mainland and the St. Lawrence-Diomede islands regions, 2004–2015 annual average. 18

S9 Figure. Waterfowl and Sandhill Crane harvest by residents of the North Slope region, 2004–2015 annual average. 19

S10 Figure. Waterfowl and Sandhill Crane harvest by residents of the Interior Alaska and Upper Copper River regions, 2004–2015 annual average. 19

S11 Table. Alaska state duck stamps issued by region of residence, 1999–2022. 20

S12 Figure. Waterfowl and Sandhill Crane harvest in Alaska by rural residents (this study) and estimates from the Harvest Information Program (HIP) survey including other user groups (Olson 2022, Seamans 2022), 2004–2015 annual average. 21

S13 Figure. Proportion of Alaska state duck stamps issued by region of residence and fall-winter rural harvest, 2005–2015 annual average. 21

S14 Figure. Alaska state duck stamps issued to residents of rural regions, 1999–2022. 22

S15 Table. Emperor Goose harvest by region of residence, 1987–2016 and 2017–2020. 23

S16 Figure. Emperor Goose harvest in Alaska, 2017–2020. 24

References 24

S1 Table. Dataset (community-years) used to estimate harvest of waterfowl and Sandhill Crane in rural Alaska representing the 2004–2015 reference period.

Year	North Slope	Northwest Arctic*	Bering Strait Mainland	St. Lawrence-Diomede Is.	Y-K Delta Coast	Y-K Delta Inland	Interior Alaska East*	Interior Alaska West	Upper Copper River*	Bristol Bay*	Aleutian-Pribilof Is.*	Kodiak Archipelago*	Gulf of Alaska-Cook Inlet	Total
1982		1												1
1983		1												1
1987							1			6	1			8
1988										1	1			2
1989										2				2
1992										1				1
1993		3										4		7
1994		2									4			6
1996		5								1	4			10
1997	1	4										3	5	13
1998	1					1		7						9
1999								7				5		12
2000	4						15							19
2001	1													1
2002	4		9	2				2						17
2003	1													1
2004			9	2	8	8	6	12	6	13			4	68
2005	7		7	3	9	11		9		16	3		1	66
2006		4	10	2	8	14	11	8		1		3	1	62
2007	5		9	2	7	9	5	2	5	13	1			58
2008	4				5	8		2		8	3			30

Year	North Slope	Northwest Arctic*	Bering Strait Mainland	St. Lawrence-Diomede Is.	Y-K Delta Coast	Y-K Delta Inland	Interior Alaska East*	Interior Alaska West	Upper Copper River*	Bristol Bay*	Aleutian-Pribilof Islands*	Kodiak Archipelago*	Gulf of Alaska-Cook Inlet	Total
2009	3			3	8	10			1					25
2010	1		5	2	7	10	8	12	2	1		5	2	55
2011	1	1		2	7	5	5	9		5				35
2012	2	5	1	2		1	2		3					16
2013		1	1	1	15	10		1	2				1	32
2014	4	1	1				9	2		3			5	25
2015					8	10	2							20
2016						2	1	2		2	3			10
2017	1		1			1	3							6
2018						1	1			3		1		6
2019	1					1	1			1				4
2020											4	5		9
Total	41	28	53	21	82	102	70	75	19	77	24	26	19	637
Number of communities§	8	11	13	3	21	26	26	21	8	31	12	12	6	202
Number of households‡	2,002	1,908	2,376	368	2,353	4,019	1,826	1,184	594	2,503	1,762	4,630	1,193	27,911
Human population‡	6,766	7,109	7,633	1,467	9,942	14,128	4,455	3,083	1,512	7,283	4,428	13,259	2,987	87,020

* Regions for which 1982–1996 data were used to supplement the dataset.
§ Communities eligible for the subsistence harvest of migratory birds in Alaska.
‡ Source: 2010 Census (U.S. Census Bureau 2021). Human population excludes people living in group quarters (e.g,, seasonal workers of seafood processing facilities), most of which are non-permanent residents and thus ineligible for the subsistence harvest of migratory birds in Alaska.

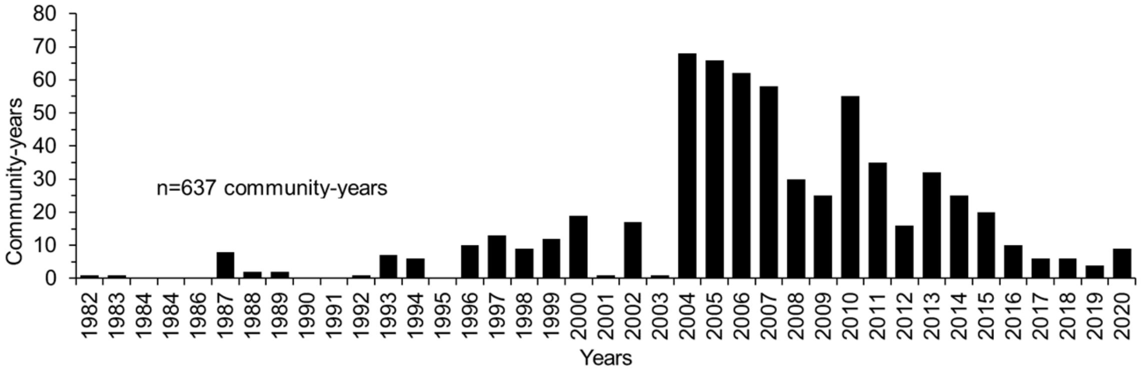

S2 Figure. Temporal distribution of the data used to estimate harvest of waterfowl and Sandhill Crane by rural hunters in Alaska representing the 2004–2015 reference period.

S3 Appendix. Formulas used to estimate harvest and confidence interval.
Note: based on Cochran (1977); see also Naves (2018) and Naves et al. (2019).

Community estimated harvest, Alaska Migratory Bird Co-Management Council data

(Equation 1) $$\hat{Y}_i = \sum_{i=1}^{i}\left[\sum_{k=1}^{i}\frac{M_{ijk}}{m_{ijk}}\left(\sum_{j=1}^{k} y_{ijk}\right)\right]$$

Region estimated harvest

(Equation 2) $$\hat{Y}_{reg} = \frac{N}{n}\sum^{n}\hat{Y}_i$$

Community variance, Alaska Migratory Bird Co-Management Council data

(Equation 3.a) $$s_i^2 = \sum_{k=1}^{i}\left[\frac{1}{m_{ijk}-1}\sum_{j=1}^{k}\left(y_{ijk}-\bar{y}_{ijk}\right)^2\right]$$

(Equation 3.b) $$\bar{y}_{ijk} = \frac{\sum_{j=1}^{k} y_{ijk}}{m_{ijk}}$$

Community variance, other data sources

(Equation 3.c) $$s_i^2 = \left[\left(\frac{CI_{PUBi}\times\hat{Y}_i}{t_{\propto/2}}\right)\times\left(\frac{\sqrt{m_{ij}}}{M_{ij}\times\sqrt{1-\frac{m_{ij}}{M_{ij}}}}\right)\right]^2$$

Region variance

(Equation 4.a) $$v(\hat{Y}_{reg}) = \frac{N^2(1-f_1)}{n}s_u^2 + \frac{N}{n}\sum_{i=1}^{n}\frac{M_i^2(1-f_2)s_i^2}{m_i}$$

(Equation 4.b) $$s_u^2 = \frac{1}{n-1}\sum_{i=1}^{n}\left(\hat{Y}_i - \hat{\bar{Y}}_{reg}\right)^2$$

(Equation 4.c) $$\hat{\bar{Y}}_{reg} = \frac{\sum_{i=1}^{n}\hat{Y}_i}{n}$$

Confidence interval at region and Alaska-wide levels

(Equation 5.a) $$CI(\hat{Y}) = 2\times CV$$

(Equation 5.b) $$CV(\hat{Y}) = \frac{\sqrt{v(\hat{Y})}}{\hat{Y}}$$

i = communities (primary sampling units)

j = households (secondary sampling units)

k = harvest level strata (Alaska Migratory Bird Co-Management Council data)

reg = region

AK = Alaska-wide

$\hat{Y}$ = estimated harvest

y = harvest reported by individual households

$\hat{\bar{Y}}_{reg}$ = average community harvest in a region

$\bar{y}_{ijk}$ = mean household harvest in community i and harvest level strata k

m = sampled households

M = total households

n = sampled communities in region

N = total communities in region

$v(\hat{Y})$ = variance of harvest estimate

$t_{1/\alpha}$ = Student's t distribution value with tail area probability α

f_1 = sampling fraction in regions (n/N)

f_2 = sampling fraction in communities (m_i/M_i)

s_i^2 = variance among households in a community

s_u^2 = variance among communities in a region

CI_{PUBi} = confidence interval published for community estimated harvest (data sources other than Alaska Migratory Bird Co-Management Council)

$CI(\hat{Y})$ = confidence interval as a percentage of the harvest estimate

$CV(\hat{Y})$ = coefficient of variation

S4 Table. Confidence intervals (as percentage of the estimate) for harvest estimates for waterfowl and Sandhill Crane, 2004–2015.

Species or categories	North Slope	North-west Arctic	Bering Strait Mainland	St. Lawrence-Diomede Is.	Y-K Delta Coast	Y-K Delta Inland	Interior Alaska East	Interior Alaska West	Upper Copper River	Bristol Bay	Aleutian-Pribilof Is.	Kodiak Archipelago	Gulf of Alaska-Cook Inlet	Rural Alaska total
Wigeons	0%	2%	14%	49%	17%	5%	27%	4%	9%	26%	4%	84%	1%	8%
Spring	0%	2%	19%	55%	21%	4%	32%	6%	-	33%	21%	74%	-	9%
Summer	-	1%	47%	50%	33%	17%	23%	139%	-	49%	-	165%	2%	12%
Fall-winter	-	3%	16%	-	19%	11%	27%	6%	9%	34%	4%	85%	1%	11%
Unknown	-	2%	-	-	-	-	55%	-	-	48%	-	-	-	45%
Gadwall	-	-	-	-	44%	-	-	-	-	45%	4%	70%	2%	59%
Spring	-	-	-	-	-	-	-	-	-	-	1%	105%	-	58%
Summer	-	-	-	-	-	-	-	-	-	-	-	-	-	-
Fall-winter	-	-	-	-	44%	-	-	-	-	51%	4%	71%	2%	64%
Unknown	-	-	-	-	-	-	-	-	-	51%	-	-	-	51%
Mallard	8%	1%	7%	7%	9%	3%	26%	2%	12%	13%	2%	31%	5%	7%
Spring	16%	2%	7%	41%	11%	3%	24%	3%	18%	15%	10%	58%	60%	7%
Summer	8%	1%	18%	33%	18%	13%	20%	12%	52%	25%	51%	76%	2%	7%
Fall-winter	-	3%	12%	5%	12%	4%	32%	4%	20%	16%	2%	33%	3%	10%
Unknown	-	2%	-	-	30%	-	28%	-	-	44%	2%	100%	-	37%
Northern Pintail	12%	5%	6%	10%	14%	3%	24%	3%	26%	14%	6%	51%	2%	5%
Spring	13%	2%	7%	10%	14%	4%	24%	3%	26%	17%	1%	99%	47%	5%
Summer	24%	25%	9%	28%	22%	15%	31%	39%	2%	34%	61%	-	2%	11%
Fall-winter	0%	4%	14%	70%	15%	5%	31%	2%	49%	21%	10%	57%	2%	7%
Unknown	-	2%	-	-	44%	-	60%	-	-	43%	-	-	-	40%
Northern Shoveler	-	4%	11%	4%	16%	5%	26%	11%	23%	23%	10%	120%	6%	6%
Spring	-	6%	12%	4%	18%	6%	23%	13%	31%	29%	-	-	155%	6%
Summer	-	5%	40%	-	27%	16%	52%	-	-	51%	-	-	-	16%

Species or categories	North Slope	North-west Arctic	Bering Strait Mainland	St. Lawrence-Diomede Is.	Y-K Delta Coast	Y-K Delta Inland	Interior Alaska East	Interior Alaska West	Upper Copper River	Bristol Bay	Aleutian-Pribilof Is.	Kodiak Archipelago	Gulf of Alaska-Cook Inlet	Rural Alaska total
Fall-winter	-	6%	23%	-	18%	9%	34%	17%	12%	28%	10%	120%	1%	8%
Unknown	-	3%	-	-	-	-	-	-	-	30%	-	-	-	26%
Teals	4%	3%	14%	69%	15%	5%	26%	3%	32%	21%	4%	57%	3%	10%
Spring	4%	3%	17%	69%	18%	7%	24%	7%	49%	29%	6%	91%	203%	11%
Summer	-	1%	22%	-	29%	18%	28%	52%	112%	39%	43%	-	2%	13%
Fall-winter	-	7%	21%	-	20%	7%	34%	5%	14%	21%	4%	52%	2%	11%
Unknown	-	2%	-	-	-	-	-	-	-	38%	-	-	-	37%
Canvasback	-	6%	22%	53%	24%	9%	25%	19%	32%	55%	8%	94%	2%	10%
Spring	-	1%	24%	53%	31%	12%	24%	25%	46%	69%	1%	-	-	10%
Summer	-	19%	84%	-	50%	62%	40%	-	2%	-	-	-	-	29%
Fall-winter	-	19%	68%	-	37%	15%	38%	16%	1%	52%	10%	94%	2%	18%
Unknown	-	-	-	-	-	-	-	-	-	51%	-	-	-	51%
Scaups	29%	2%	34%	-	22%	3%	33%	19%	53%	31%	47%	51%	5%	10%
Spring	53%	3%	36%	-	24%	3%	32%	23%	75%	36%	1%	98%	22%	8%
Summer	35%	1%	4%	-	46%	13%	39%	-	-	59%	43%	-	-	11%
Fall-winter	-	5%	88%	-	24%	9%	41%	13%	42%	48%	56%	38%	6%	17%
Unknown	-	3%	-	-	-	-	-	-	-	41%	-	-	-	16%
Common Eider	5%	8%	15%	6%	19%	9%	-	2%	-	41%	2%	100%	-	4%
Spring	9%	11%	18%	7%	23%	6%	-	2%	-	46%	-	-	-	6%
Summer	4%	1%	37%	7%	46%	-	-	-	-	85%	1%	-	-	4%
Fall-winter	2%	1%	32%	9%	34%	79%	-	-	-	52%	2%	100%	-	7%
Unknown	5%	-	-	-	-	-	-	-	-	-	-	-	-	5%
King Eider	4%	2%	30%	14%	21%	14%	-	-	-	41%	1%	47%	-	8%
Spring	4%	3%	30%	14%	22%	16%	-	-	-	41%	27%	104%	-	11%
Summer	5%	1%	5%	10%	48%	-	-	-	-	-	-	-	-	8%

Species or categories	North Slope	North-west Arctic	Bering Strait Mainland	St. Lawrence-Diomede Is.	Y-K Delta Coast	Y-K Delta Inland	Interior Alaska East	Interior Alaska West	Upper Copper River	Bristol Bay	Aleutian-Pribilof Is.	Kodiak Archipelago	Gulf of Alaska-Cook Inlet	Rural Alaska total
Fall-winter	1%	2%	88%	24%	42%	35%	-	-	-	57%	0%	52%	-	13%
Unknown	-	-	-	-	-	-	-	-	-	-	-	-	-	-
Spectacled Eider	8%	-	68%	18%	38%	4%	-	2%	2%	70%	21%	-	-	12%
Spring	3%	-	26%	21%	49%	4%	-	2%	-	89%	-	-	-	12%
Summer	12%	-	71%	14%	104%	-	-	-	2%	-	-	-	-	11%
Fall-winter	-	-	86%	39%	74%	-	-	-	2%	87%	21%	-	-	33%
Unknown	-	-	-	-	-	-	-	-	-	-	-	-	-	-
Steller's Eider	7%	2%	24%	28%	32%	86%	-	-	-	59%	3%	-	-	10%
Spring	8%	-	-	62%	39%	97%	-	-	-	65%	-	-	-	19%
Summer	18%	-	62%	23%	46%	-	-	-	-	-	-	-	-	19%
Fall-winter	-	2%	25%	37%	55%	-	-	-	-	57%	3%	-	-	13%
Unknown	-	-	-	-	-	-	-	-	-	-	-	-	-	-
Eiders (unidentified)	0%	0%	2%	-	44%	3%	-	-	-	35%	-	100%	-	3%
Spring	0%	0%	2%	-	44%	3%	-	-	-	44%	-	-	-	1%
Summer	0%	0%	4%	-	-	-	-	-	-	-	-	-	-	0%
Fall-winter	2%	0%	4%	-	-	4%	-	-	-	-	-	100%	-	6%
Unknown	-	-	-	-	-	-	-	-	-	38%	-	-	-	38%
Surf Scoter	-	1%	61%	51%	22%	5%	29%	31%	-	32%	3%	53%	32%	14%
Spring	-	2%	73%	85%	24%	6%	37%	38%	-	41%	47%	93%	70%	15%
Summer	-	1%	68%	5%	94%	62%	45%	-	-	70%	-	-	-	20%
Fall-winter	-	4%	69%	45%	49%	21%	52%	41%	-	48%	2%	41%	34%	23%
Unknown	-	3%	-	-	-	-	47%	-	-	-	-	-	-	38%
White-winged Scoter	21%	2%	35%	30%	29%	6%	22%	50%	369%	40%	17%	41%	34%	12%

Species or categories	North Slope	North-west Arctic	Bering Strait Mainland	St. Lawrence-Diomede Is.	Y-K Delta Coast	Y-K Delta Inland	Interior Alaska East	Interior Alaska West	Upper Copper River	Bristol Bay	Aleutian-Pribilof Is.	Kodiak Archipelago	Gulf of Alaska-Cook Inlet	Rural Alaska total
Spring	2%	2%	35%	101%	33%	7%	22%	74%	369%	57%	2%	81%	100%	12%
Summer	39%	1%	-	43%	60%	49%	33%	-	369%	-	-	-	31%	30%
Fall-winter	-	3%	-	47%	48%	12%	28%	30%	-	40%	17%	34%	31%	15%
Unknown	-	-	-	-	-	-	56%	-	-	-	-	-	-	56%
Black Scoter	59%	2%	11%	8%	20%	3%	22%	1%	92%	28%	13%	58%	9%	13%
Spring	74%	3%	13%	168%	22%	3%	22%	1%	1%	31%	35%	94%	20%	11%
Summer	-	1%	-	5%	37%	24%	44%	2%	2%	93%	43%	-	2%	16%
Fall-winter	25%	3%	10%	9%	26%	8%	34%	14%	369%	40%	14%	48%	10%	26%
Unknown	-	1%	-	-	-	-	-	-	-	-	-	-	2%	1%
Scoters (unidentified)	-	2%	-	-	-	-	63%	2%	-	36%	2%	100%	2%	49%
Spring	-	-	-	-	-	-	67%	1%	-	41%	-	-	-	62%
Summer	-	-	-	-	-	-	-	-	-	-	-	-	-	-
Fall-winter	-	2%	-	-	-	-	65%	2%	-	-	2%	100%	2%	57%
Unknown	-	-	-	-	-	-	44%	-	-	51%	-	-	-	40%
Harlequin Duck	-	14%	150%	8%	51%	9%	64%	28%	-	26%	8%	65%	24%	46%
Spring	-	18%	498%	17%	57%	13%	88%	32%	-	32%	19%	93%	112%	70%
Summer	-	-	194%	15%	-	72%	83%	-	-	-	35%	87%	90%	23%
Fall-winter	-	2%	119%	20%	61%	11%	70%	51%	-	29%	9%	51%	17%	37%
Unknown	-	-	-	-	-	-	-	-	-	-	1%	100%	2%	72%
Long-tailed Duck	13%	2%	15%	33%	35%	8%	24%	16%	-	35%	1%	34%	17%	8%
Spring	35%	3%	17%	46%	29%	9%	23%	17%	-	41%	-	86%	50%	9%
Summer	16%	1%	5%	22%	46%	27%	45%	-	-	-	-	-	-	15%
Fall-winter	3%	2%	26%	22%	42%	14%	51%	14%	-	62%	1%	34%	8%	18%
Unknown	2%	3%	-	-	-	-	-	-	-	-	-	-	-	2%

Species or categories	North Slope	North-west Arctic	Bering Strait Mainland	St. Lawrence-Diomede Is.	Y-K Delta Coast	Y-K Delta Inland	Interior Alaska East	Interior Alaska West	Upper Copper River	Bristol Bay	Aleutian-Pribilof Is.	Kodiak Archipelago	Gulf of Alaska-Cook Inlet	Rural Alaska total
Bufflehead	-	2%	25%	-	43%	11%	43%	4%	87%	40%	5%	39%	9%	23%
Spring	-	2%	26%	-	55%	11%	47%	4%	147%	38%	-	68%	52%	27%
Summer	-	2%	-	-	-	65%	57%	-	-	-	43%	120%	90%	41%
Fall-winter	-	-	3%	-	56%	21%	41%	2%	42%	63%	5%	40%	9%	27%
Unknown	-	-	-	-	-	-	47%	-	-	51%	-	100%	-	77%
Goldeneyes	0%	9%	26%	20%	27%	5%	24%	8%	63%	20%	8%	28%	12%	13%
Spring	-	4%	11%	58%	34%	6%	22%	8%	132%	23%	-	43%	34%	8%
Summer	0%	2%	-	55%	64%	16%	52%	-	369%	73%	43%	120%	2%	19%
Fall-winter	-	91%	76%	22%	33%	17%	31%	45%	30%	25%	8%	29%	12%	19%
Unknown	-	-	-	-	-	-	40%	-	-	35%	2%	100%	2%	59%
Mergansers	2%	9%	125%	22%	40%	20%	43%	26%	1%	28%	7%	45%	12%	13%
Spring	2%	-	125%	42%	46%	25%	50%	26%	1%	29%	31%	64%	29%	17%
Summer	-	12%	-	36%	100%	111%	91%	-	-	52%	30%	-	-	42%
Fall-winter	-	3%	-	34%	43%	29%	65%	-	-	36%	6%	49%	13%	16%
Unknown	-	-	-	-	-	-	-	-	-	36%	0%	-	-	34%
Ducks (other, unidentified)	1%	2%	34%	50%	27%	10%	26%	2%	39%	32%	17%	97%	1%	9%
Spring	1%	2%	17%	58%	25%	11%	31%	3%	50%	32%	35%	-	3%	7%
Summer	2%	1%	40%	-	45%	34%	48%	-	97%	94%	35%	-	2%	33%
Fall-winter	4%	3%	53%	57%	32%	23%	38%	4%	3%	39%	10%	89%	1%	9%
Unknown	-	3%	-	-	-	-	-	-	-	51%	-	100%	-	88%
Ducks, total	2%	2%	4%	9%	8%	2%	30%	3%	27%	15%	1%	65%	8%	6%
Spring	2%	1%	3%	7%	7%	2%	33%	2%	10%	17%	1%	101%	63%	6%
Summer	4%	22%	8%	7%	17%	9%	29%	5%	-	41%	14%	-	2%	5%
Fall-winter	1%	1%	5%	9%	12%	2%	26%	4%	40%	23%	3%	57%	3%	9%

Species or categories	North Slope	North-west Arctic	Bering Strait Mainland	St. Lawrence-Diomede Is.	Y-K Delta Coast	Y-K Delta Inland	Interior Alaska East	Interior Alaska West	Upper Copper River	Bristol Bay	Aleutian-Pribilof Is.	Kodiak Archipelago	Gulf of Alaska-Cook Inlet	Rural Alaska total
Unknown	0%	1%	-	-	-	-	68%	-	-	33%	1%	-	-	32%
White-fronted Goose	3%	2%	5%	27%	10%	2%	36%	4%	2%	15%	-	-	2%	4%
Spring	3%	1%	5%	24%	9%	2%	38%	4%	-	17%	-	-	-	4%
Summer	8%	56%	18%	-	20%	14%	34%	26%	-	37%	-	-	2%	12%
Fall-winter	2%	2%	18%	67%	14%	4%	31%	3%	2%	28%	-	-	2%	8%
Unknown	0%	-	-	-	-	-	68%	-	-	38%	-	-	-	35%
Snow Goose	4%	4%	5%	9%	31%	14%	48%	2%	1%	45%	2%	-	2%	7%
Spring	4%	4%	6%	18%	40%	17%	51%	3%	1%	51%	-	-	-	8%
Summer	12%	-	26%	16%	130%	31%	42%	1%	-	-	-	-	-	13%
Fall-winter	0%	2%	14%	11%	39%	14%	47%	2%	2%	51%	2%	-	2%	13%
Unknown	-	-	-	-	-	-	-	-	-	-	-	-	-	-
Brant	6%	3%	7%	8%	11%	9%	55%	43%	58%	36%	2%	59%	2%	5%
Spring	7%	3%	8%	12%	12%	11%	54%	47%	42%	44%	1%	-	-	6%
Summer	8%	4%	25%	14%	23%	14%	-	-	-	48%	125%	-	-	9%
Fall-winter	2%	12%	16%	11%	17%	27%	76%	44%	96%	29%	1%	59%	2%	7%
Unknown	-	-	-	-	-	-	-	-	-	46%	-	-	-	46%
Canada/Cackling geese	6%	2%	5%	16%	9%	2%	23%	3%	40%	17%	2%	56%	9%	4%
Spring	7%	1%	5%	30%	8%	2%	25%	3%	19%	16%	1%	102%	100%	3%
Summer	20%	40%	13%	27%	19%	13%	26%	5%	-	41%	1%	-	2%	12%
Fall-winter	1%	2%	8%	14%	14%	3%	26%	6%	115%	27%	2%	46%	5%	7%
Unknown	0%	1%	-	-	-	-	68%	-	-	29%	-	-	-	26%
Emperor Goose	56%	2%	17%	12%	13%	14%	-	33%	-	24%	19%	92%	-	7%
Spring	56%	2%	18%	7%	17%	5%	-	-	-	27%	42%	-	-	10%
Summer	-	-	28%	9%	31%	37%	-	-	-	166%	49%	-	-	16%

Species or categories	North Slope	North-west Arctic	Bering Strait Mainland	St. Lawrence-Diomede Is.	Y-K Delta Coast	Y-K Delta Inland	Interior Alaska East	Interior Alaska West	Upper Copper River	Bristol Bay	Aleutian-Pribilof Is.	Kodiak Archipelago	Gulf of Alaska-Cook Inlet	Rural Alaska total
Fall-winter	-	-	42%	19%	21%	39%	-	34%	-	26%	23%	92%	-	12%
Unknown	-	-	-	-	-	-	-	-	-	-	52%	-	-	52%
Geese (unidentified)	2%	4%	-	-	-	4%	35%	1%	1%	31%	1%	100%	2%	6%
Spring	2%	5%	-	-	-	3%	59%	1%	-	33%	-	-	3%	7%
Summer	-	0%	-	-	-	-	54%	-	-	-	-	-	-	28%
Fall-winter	-	0%	-	-	-	11%	68%	2%	1%	28%	1%	100%	2%	7%
Unknown	-	-	-	-	-	-	-	-	-	-	-	-	-	-
Geese, total	2%	2%	4%	9%	8%	2%	30%	3%	27%	15%	1%	65%	8%	3%
Spring	2%	1%	3%	7%	7%	2%	33%	2%	10%	17%	1%	101%	63%	3%
Summer	4%	22%	8%	7%	17%	9%	29%	5%	-	41%	14%	-	2%	10%
Fall-winter	1%	1%	5%	9%	12%	2%	26%	4%	40%	23%	3%	57%	3%	6%
Unknown	0%	1%	-	-	-	-	68%	-	-	33%	1%	65%	-	30%
Swans	10%	2%	8%	13%	10%	3%	50%	11%	46%	17%	-	-	103%	4%
Spring	10%	2%	10%	14%	10%	3%	42%	11%	75%	18%	-	-	-	4%
Summer	1%	10%	29%	20%	26%	14%	-	-	-	54%	-	-	-	15%
Fall-winter	2%	3%	11%	37%	14%	5%	70%	62%	1%	34%	-	-	103%	7%
Unknown	-	2%	5%	-	44%	-	68%	-	-	37%	-	-	-	32%
Sandhill Crane	16%	3%	6%	11%	10%	3%	33%	17%	-	19%	1%	-	3%	5%
Spring	19%	3%	7%	11%	10%	4%	33%	12%	-	28%	-	-	-	5%
Summer	18%	15%	16%	10%	22%	16%	57%	73%	-	25%	1%	-	2%	10%
Fall-winter	-	3%	9%	45%	22%	8%	39%	18%	-	24%	2%	-	8%	9%
Unknown	-	-	5%	-	44%	-	68%	-	-	42%	-	-	-	37%

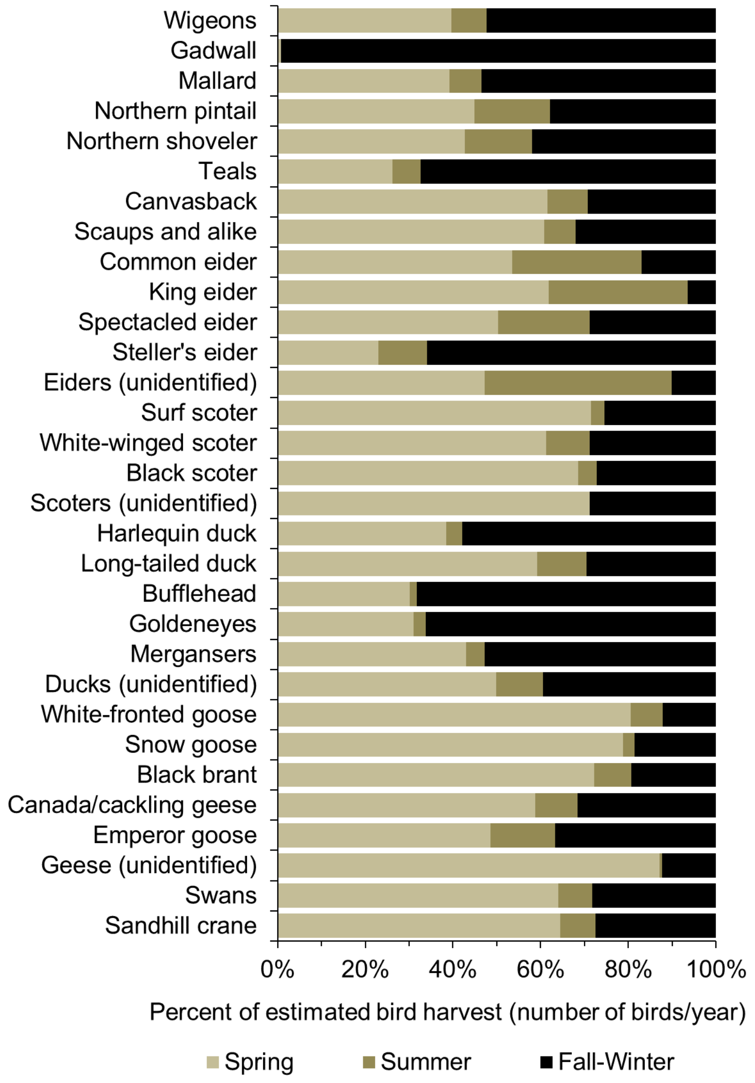

S5 Figure. Seasonality of the harvest of waterfowl and Sandhill Crane by rural hunters in Alaska, annual average 2004–2015.

S6 Table. Confidence intervals (as percentage of the estimate) for harvest estimates for eggs of waterfowl and Sandhill Crane, 2004–2015.

**Species or categories**	**North Slope**	**North-west Arctic**	**Bering Strait Mainland**	**St. Lawrence-Diomede Is.**	**Y-K Delta Coast**	**Y-K Delta Inland**	**Interior Alaska East**	**Interior Alaska West**	**Upper Copper River**	**Bristol Bay**	**Aleutian-Pribilof Is.**	**Kodiak Archipelago**	**Gulf of Alaska-Cook Inlet**	**Rural Alaska total**
Wigeons	-	3%	6%	-	32%	35%	86%	-	-	77%	-	-	-	20%
Gadwall	-	-	-	-	-	-	-	-	-	-	-	-	-	-
Mallard	79%	11%	29%	94%	23%	8%	45%	-	333%	36%	-	99%	213%	10%
Northern Pintail	2%	29%	15%	4%	19%	16%	58%	33%	211%	38%	-	-	-	10%
Northern Shoveler	-	91%	65%	-	28%	50%	54%	-	333%	-	-	-	-	22%
Teals	-	-	66%	-	27%	32%	79%	-	333%	49%	-	-	-	19%
Canvasback	-	-	42%	-	59%	58%	69%	-	-	-	-	-	-	48%
Scaups	-	2%	49%	-	66%	84%	-	-	-	82%	-	-	-	35%
Common Eider	80%	2%	10%	26%	40%	-	-	-	-	-	1%	-	-	9%
King Eider	16%	1%	15%	50%	42%	-	-	-	-	189%	47%	-	-	12%
Spectacled Eider	-	-	49%	101%	-	-	-	-	-	-	-	-	-	44%
Steller's Eider	-	-	102%	94%	131%		-	-	-	-	-	-	-	71%
Eiders (unidentified)	2%	2%	2%	-	-	-	-	-	-	-	2%	100%	-	4%
Surf Scoter	-	-	-	-	75%	-	77%	-	-	-	-	104%	-	58%
White-winged Scoter	93%	-	118%	-	144%	-	59%	-	-	-	-	104%	-	47%
Black Scoter	111%	-	73%	-	52%	47%	91%	-	-	-	-	104%	-	46%
Scoters (unidentified)	-	-	-	-	-	-	-	-	-	-	-	-	-	-
Harlequin Duck	-	-	-	-	-	-	-	-	-	-	-	-	-	-
Long-tailed Duck		-						-	-	-	-	-	-	30%
Bufflehead	-	-	131%	-	73%	-	-	122%	333%	-	-	85%	-	63%
Goldeneyes	-	-	3%	58%	65%	-	-	-	-	-	-	97%	-	70%

Species or categories	North Slope	North-west Arctic	Bering Strait Mainland	St. Lawrence-Diomede Is.	Y-K Delta Coast	Y-K Delta Inland	Interior Alaska East	Interior Alaska West	Upper Copper River	Bristol Bay	Aleutian-Pribilof Is.	Kodiak Archipelago	Gulf of Alaska-Cook Inlet	Rural Alaska total
Mergansers	62%	-	6%	4	-	143	-	-	-	81%	35%	76%	-	15%
Ducks (other, unidentified)	8%	1%	5%	-	24	2	46	-	-	21%	33%	81%	2%	10%
Ducks, total	24%	9%	8%	35%	13%	7%	44%	32%	282%	18%	21%	67%	23%	5%
White-fronted Goose	10%	38%	27%	58%	16%	15%	50%	-	-	94%	-	-	-	14%
Snow Goose	26%	-	34%	-	43%	23%	-	-	-	-	-	-	-	19%
Brant	17%	18%	20%	59%	24%	39%	-	-	-	-	-	-	-	20%
Canada/Cackling geese	21%	23%	9%	58%	15%	10%	50%	-	-	33%	-	-		11%
Emperor Goose	-	-	18%	58%	26%	33%	-	-	-	157%	-	-	-	22%
Geese (unidentified)	2%	3%	2%	-	31%	1%	67%	-	-	38%	-	100%	1%	11%
Geese, total	4%	14%	7%	40%	14%	6%	45%	-	-	27%	-	100%	36%	10%
Swans	9%	26%	12%	54%	17%	7%	72%	-	-	30%	-	-	-	9%
Sandhill Crane	1%	23%	10%	63%	19%	15%	76%	-	-	101%	-	-	-	12%

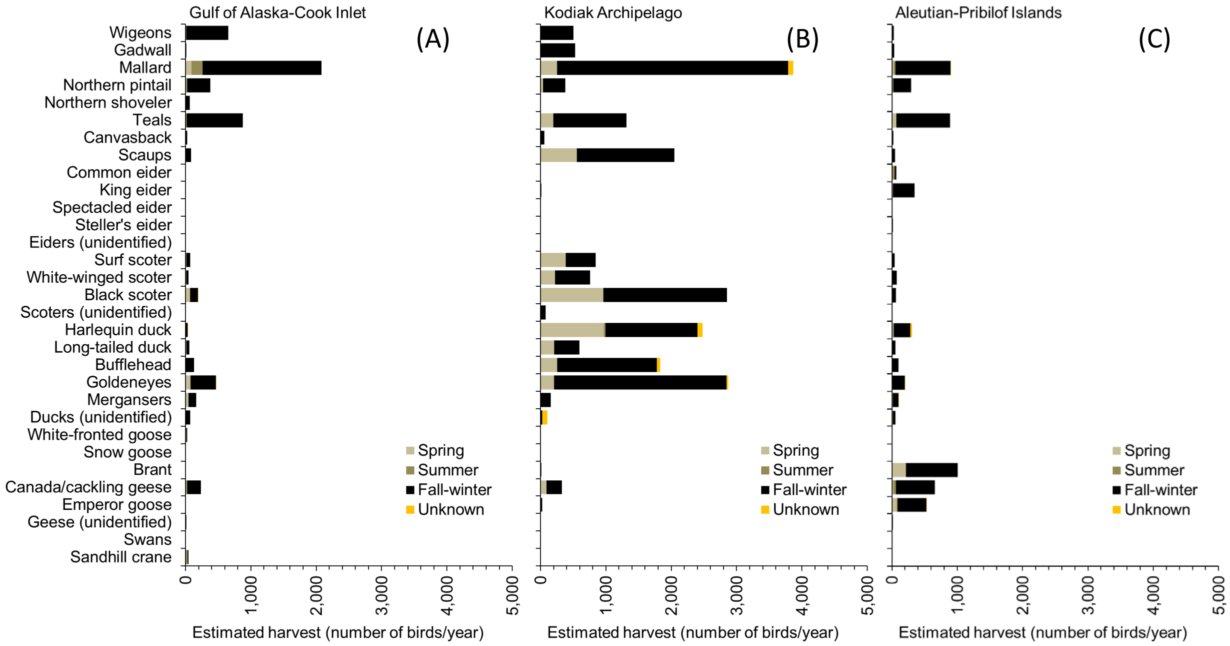

S7 Figure. Waterfowl and Sandhill Crane harvest by residents of the Pacific-Aleutian mainland and islands regions, 2004–2015 annual average.

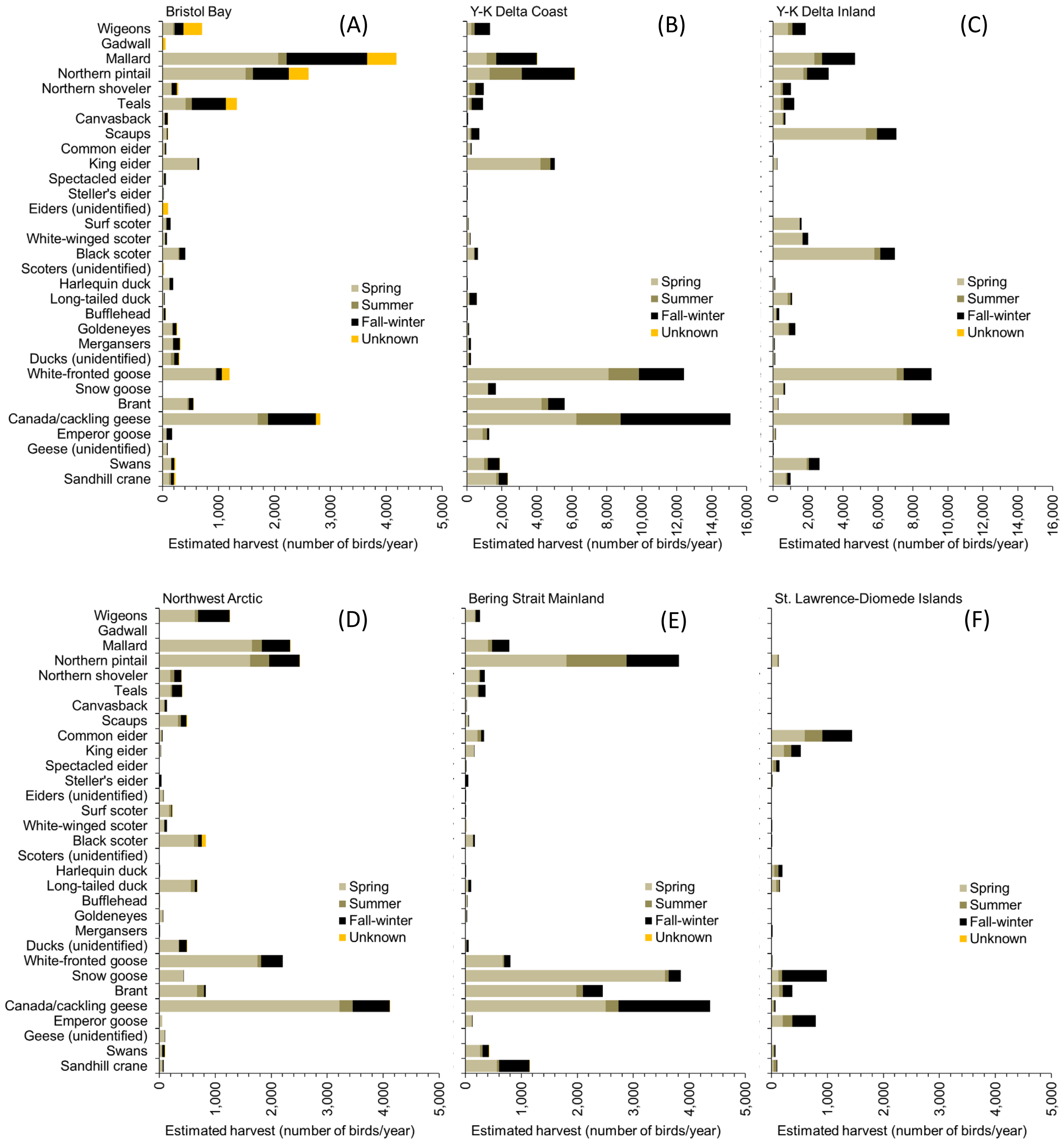

S8 Figure. Waterfowl and Sandhill Crane harvest by residents of the Bering Sea mainland and the St. Lawrence-Diomede islands regions, 2004–2015 annual average.

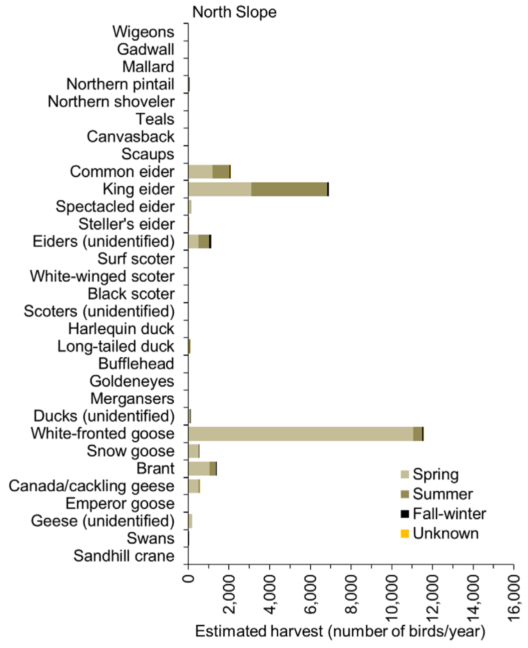

S9 Figure. Waterfowl and Sandhill Crane harvest by residents of the North Slope region, 2004–2015 annual average.

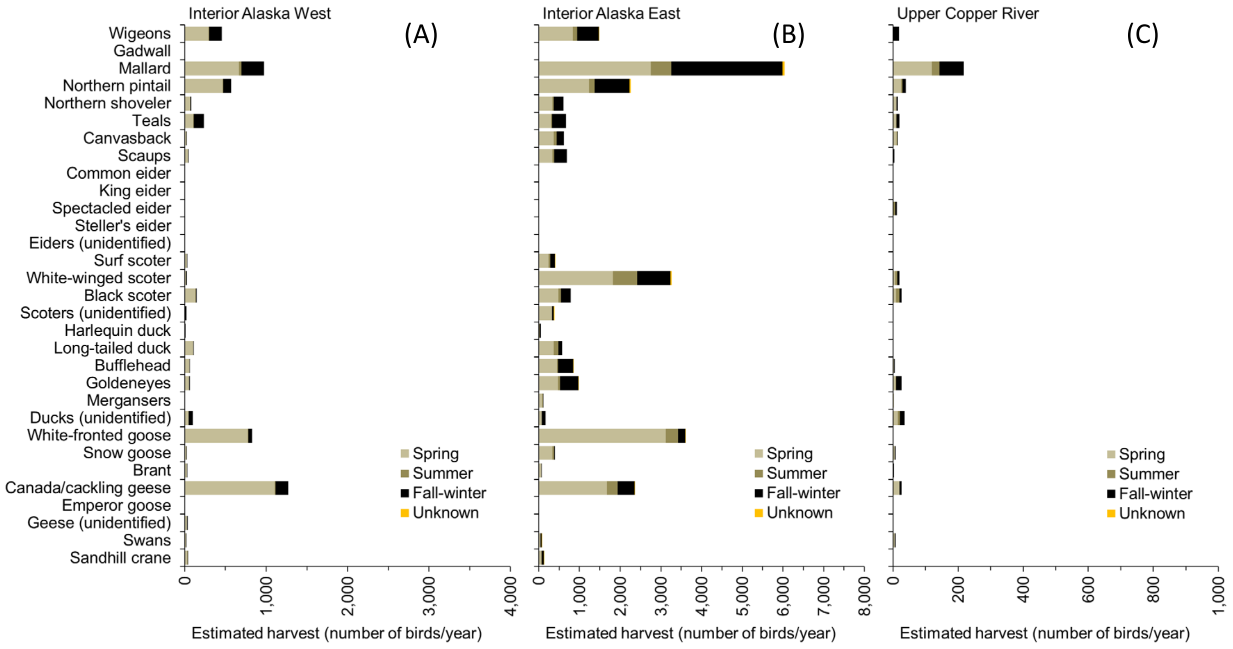

S10 Figure. Waterfowl and Sandhill Crane harvest by residents of the Interior Alaska and Upper Copper River regions, 2004–2015 annual average.

S11 Table. Alaska state duck stamps issued by region of residence, 1999–2022.

Year	Alaska rural regions (eligible for the spring-summer subsistence harvest of migratory birds)														Alaska urban regions (ineligible)	Other USA states (ineligible)	Other countries (ineligible)	Total
	North Slope	Northwest Arctic	Bering Strait Mainland	St. Lawrence-Diomede Is.	Y-K Delta Coast	Y-K Delta Inland	Interior Alaska East	Interior Alaska West	Upper Copper River	Bristol Bay	Aleutian-Pribilof Is.	Kodiak Archipelago	Gulf of AK-Cook Inlet	Sub-total				
1999	8	27	126	0	5	118	96	49	25	163	120	384	186	1,307	6,818	1,121	15	9,261
2000	8	33	127	0	7	76	88	55	19	165	122	354	223	1,277	7,380	1,064	26	9,747
2001	3	28	128	1	6	92	95	25	18	180	68	407	208	1,259	7,147	987	18	9,411
2002	2	28	83	0	9	111	91	35	22	140	89	307	205	1,122	6,967	812	32	8,933
2003	3	30	80	0	12	117	104	34	20	145	105	342	211	1,203	7,207	952	7	9,369
2004	2	19	76	0	9	116	75	16	12	136	59	301	223	1,044	6,458	834	8	8,344
2005	2	16	95	1	6	140	82	35	17	161	96	335	225	1,211	6,462	1,141	13	8,827
2006	6	52	91	0	25	192	103	49	22	165	92	354	188	1,339	6,379	1,099	25	8,842
2007	5	41	78	0	13	141	95	41	19	138	106	377	195	1,249	6,374	1,234	20	8,877
2008	11	39	96	0	16	107	82	20	17	134	77	384	188	1,171	6,502	847	18	8,538
2009	18	46	111	0	12	131	70	38	23	152	84	336	202	1,223	6,545	854	14	8,636
2010	141	124	221	1	77	247	113	90	29	278	108	418	194	2,041	6,664	959	19	9,683
2011	81	110	213	0	32	100	154	96	28	297	101	393	221	1,826	6,282	940	27	9,075
2012	114	128	238	1	28	105	143	98	21	301	100	430	229	1,936	6,591	1,026	21	9,574
2013	99	140	188	0	28	315	150	97	18	350	82	460	244	2,171	7,565	912	22	10,670
2014	73	176	220	0	14	213	147	97	30	343	81	445	248	2,087	8,061	1,051	23	11,222
2015	32	115	193	0	18	132	119	83	22	247	89	385	228	1,663	7,282	1,073	25	10,043
2016	42	199	207	0	25	157	104	85	21	334	72	422	132	1,800	7,671	1,391	24	10,886
2017	45	155	216	1	23	123	106	63	20	287	73	391	214	1,717	7,031	1,436	10	10,194
2018	24	144	203	3	18	130	101	39	27	257	68	386	212	1,612	6,893	1,458	15	9,978
2019	16	145	211	0	26	138	80	49	19	278	62	406	190	1,620	6,592	1,650	49	9,911
2020	23	87	196	0	19	110	85	43	29	249	63	430	222	1,556	7,723	1,318	4	10,601
2021	22	68	176	0	9	82	84	33	30	185	51	337	179	1,256	6,500	1,943	1	9,700
2022	10	88	164	0	11	82	86	33	28	179	59	346	180	1,266	6,750	1,913	7	9,936

Source: Alaska Department of Fish and Game-Division of Administrative Services unpublished data.

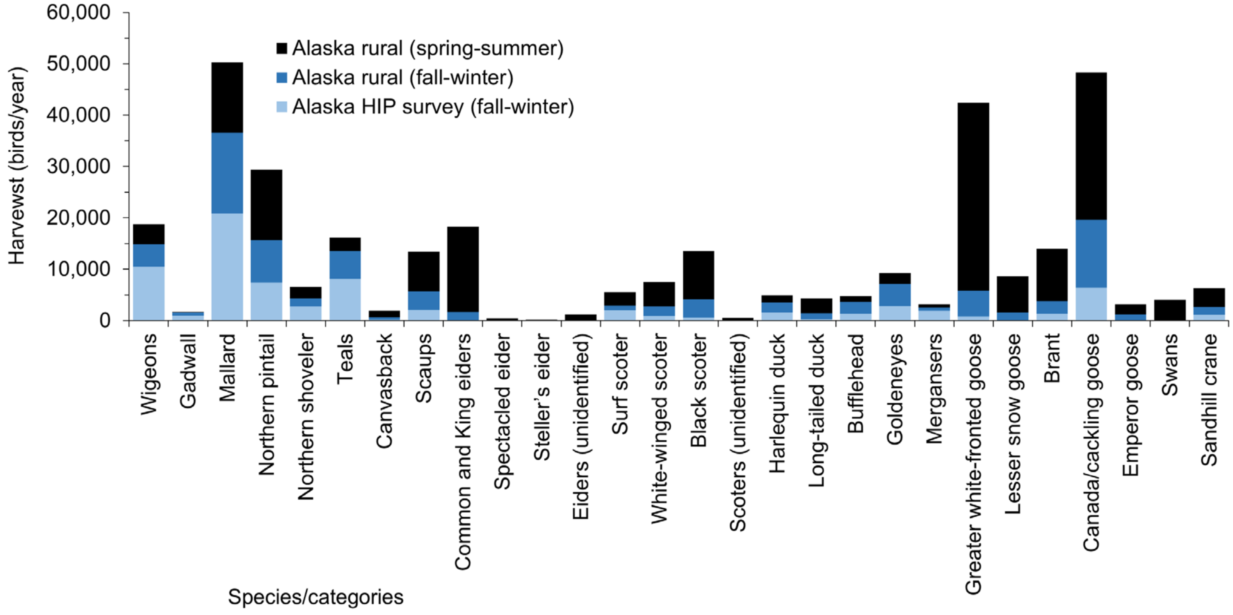

S12 Figure. Waterfowl and Sandhill Crane harvest in Alaska by rural residents (this study) and estimates from the Harvest Information Program (HIP) survey including other user groups (Olson 2022, Seamans 2022), 2004–2015 annual average.

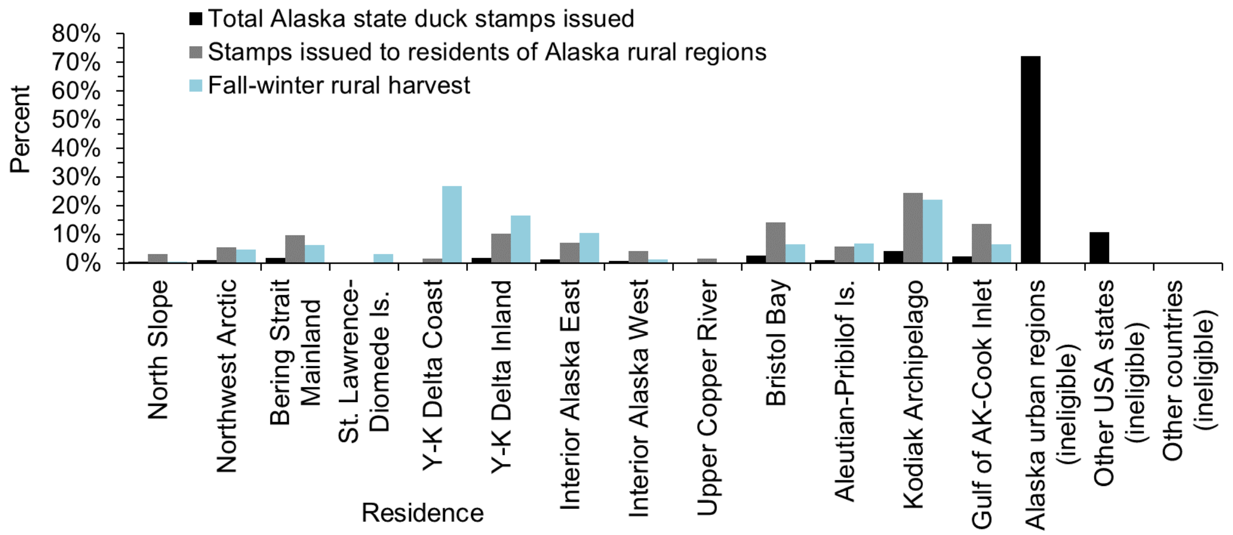

S13 Figure. Proportion of Alaska state duck stamps issued by region of residence and fall-winter rural harvest, 2005–2015 annual average.
Note: Rural regions were defined in this study as those eligible to participate in the Alaska spring-summer subsistence harvest of migratory birds.
Sources: Fall-winter harvest estimate for rural regions in Alaska (this study); Alaska state duck stamps issued (Alaska Department of Fish and Game-Division of Administrative Services unpublished data).

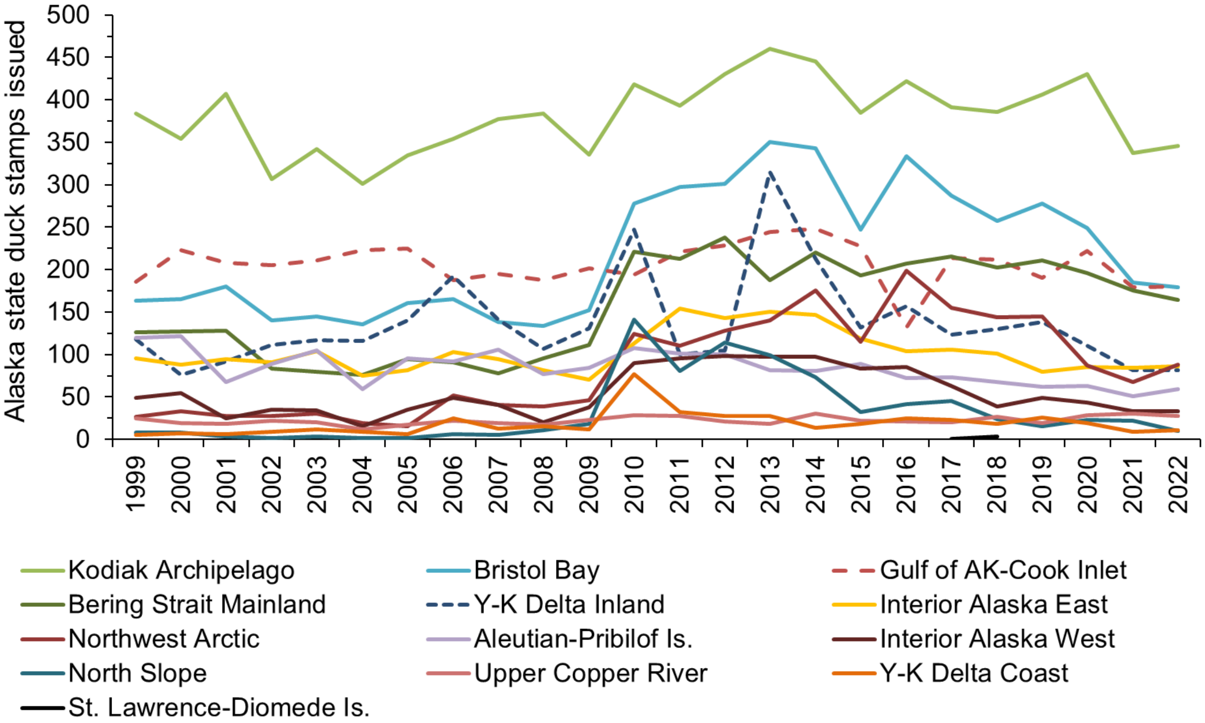

S14 Figure. Alaska state duck stamps issued to residents of rural regions, 1999–2022.
Note: Rural regions were defined in this study as those eligible to participate in the Alaska spring-summer subsistence harvest of migratory birds. Source: Alaska Department of Fish and Game-Division of Administrative Services unpublished data.

S15 Table. Emperor Goose harvest by region of residence, 1987–2016 and 2017–2020.

Regions	1987–2016§			2017–2020		
	Spring-summer	Fall-winter	Total	Spring-summer	Fall-winter	Total
North Slope	1	0	1	-	-	-
Northwest Arctic	55	0	55	-	-	-
Bering Strait-Norton Sound	492	436	928	1,074‡	650‡	1,724‡
Mainland	121	13	134	-	-	-
St. Lawrence-Diomede Is.	371	423	794	-	-	-
Yukon-Kuskokwim Delta	1,283	180	1,463	3,402‡	342‡	3,744‡
Coast	1,142	143	1,285	-	-	-
Inland	141	37	178	-	-	-
Interior Alaska	0	2	2	-	-	-
East	0	0	0	-	-	-
West	0	2	2	-	-	-
Upper Copper River	0	0	0	-	-	-
Bristol Bay	71	98	169	165‡	61‡	226‡
Aleutian-Pribilof Is.	86	450	536	16*	194*	210*
Kodiak Archipelago	0	25	25	16*	229*	245*
Gulf of Alaska-Cook Inlet	0	0	0	-	-	-
Alaska urban resident	-	-	-	-	77ǂ	77ǂ
Non-Alaska resident	-	-	-	-	25ǂ	25ǂ
Total	1,988	1,191	3,179	4,673	1,577	6,251

-: Data unavailable. Sources:
(§) This study; annual average harvest estimate for 2004–2015 reference period included 1987–2016 data.
(‡) 2017–2019 Annual average harvest estimate from survey of the Alaska Migratory Bird Co-Management Council (Naves et al. 2023).
(*) 2020 Annual harvest estimate for Aleutian-Pribilof Islands and Kodiak Archipelago (Naves and Mengak 2023).
(ǂ) Emperor Goose fall-winter permit reporting (Naves et al. 2023).

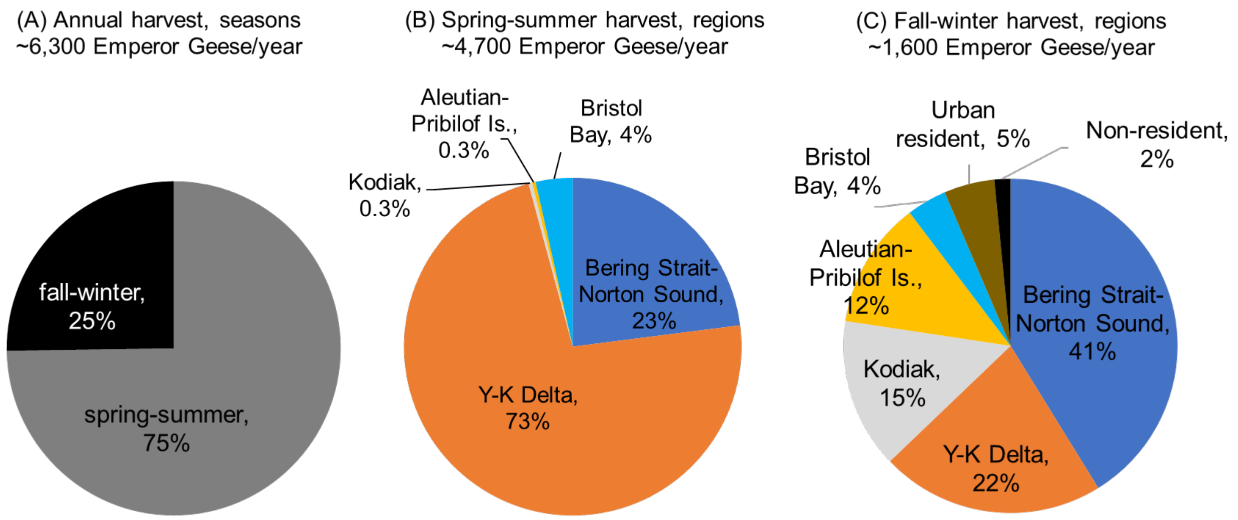

S16 Figure. Emperor Goose harvest in Alaska, 2017–2020.
Sources: 2020 Annual harvest estimate for Aleutian-Pribilof Islands and Kodiak Archipelago (Naves and Mengak 2023). 2017–2019 Annual average harvest estimate for Yukon-Kuskokwim (Y-K) Delta, Bering Strait-Norton Sound, and Bristol Bay (harvest survey of the Alaska Migratory Bird Co-Management Council); Alaska urban residents and non-residents (fall-winter Emperor Goose permit reporting) (Naves et al. 2023).

References

Cochran WG (1977) Sampling techniques. 3rd ed. New York: John Wiley & Sons.

Naves LC (2018) Geographic and seasonal patterns of seabird subsistence harvest in Alaska. Polar Biol. 41:1217–1236.

Naves LC, Keating JM, Tibbitts TL, Ruthrauff DR (2019) Shorebird subsistence harvest and indigenous knowledge in Alaska: Informing harvest management and engaging users in shorebird conservation. Condor 121:1–19.

Naves LC, Mengak LF (2023) Bird and egg harvest on the Aleutian-Pribilof Islands and Kodiak Archipelago, 2020. Anchorage (AK): Alaska Department of Fish and Game, Division of Subsistence Technical Paper No. 493.

Naves LC, Schamber JL, Mengak LF, Keating JM, Fall JA (2023) Emperor Goose fall–winter harvest monitoring and hunter's perspectives in Alaska. Conservation Science and Practice, e12928. http://doi.org/10.1111/csp2.12928.

Olson SM [compiler] (2022) Pacific flyway data book, 2022. Helena (MT): Division of Migratory Bird Management, U.S. Fish and Wildlife Service.

Seamans ME (2022) Status and harvests of Sandhill Cranes: Mid-Continent, Rocky Mountain, Lower Colorado River Valley, and Eastern Populations. Administrative report. Lakewood (CO): U.S. Fish and Wildlife Service.

U.S. Census Bureau (2021) Explore Census Data. U.S. Department of Commerce, Bureau of the Census, American FactFinder Homepage: Washington, D.C. https://data.census.gov/.
