# Supplementary material for: Harvest of waterfowl and Sandhill Crane in rural Alaska: Geographic and seasonal patterns
Source: PLoS One. 2024 Jul 25;19(7):e0307135. doi: 10.1371/journal.pone.0307135 (PMC11271962; doi:10.1371/journal.pone.0307135)
Supplement: S2 File — (PDF) [file pone.0307135.s002.pdf]

SUBSISTENCE MIGRATORY BIRD
HARVEST SURVEY REPORT

FOR THE

KODIAK ISLAND VILLAGES

MARCH 11, 1999 - MARCH 10, 2000

BY

ROBERT STOVALL

KODIAK NATIONAL WILDLIFE REFUGE
1390 BUSKIN RIVER ROAD
KODIAK, ALASKA 99615

IN COOPERATION WITH THE KODIAK ISLAND VILLAGES OF:

AKHIOK
KARLUK
LARSEN BAY
OLD HARBOR
OUZINKIE
PORT LIONS

SEPTEMBER 2000

INTRODUCTION:

In 1999, the Kodiak National Wildlife Refuge implemented a plan to complete migratory bird subsistence harvest surveys of the six Kodiak Island villages. Subsistence uses of all fish and game resources by the inhabitants of Kodiak Island Archipelago is historically well documented. Waterfowl hunting and egg gathering is part of the annual cycle for all village and road system subsistence users. Waterfowl is primarily harvested in the fall through late winter while egg gathering activities occur in the late spring and early summer.

Kodiak Island villages are comprised of mixed races, with a higher proportion of native than non-native in all villages. Kodiak City, road system, and Coast Guard base households had an extensive subsistence harvest survey completed in 1992 and was not included in this survey.(Mishler 1992)

The purpose of this survey is to determine the level at which Kodiak Island village residents participate in subsistence migratory bird harvest activities, the species composition of the harvests, and the relative importance of spring/summer harvests. Data were collected on both bird harvests and egg gathering activities.

METHODS: (Figure 1)

The Kodiak Island villages surveyed included Akhiok, Karluk, Larsen Bay, Old Harbor, Ouzinkie, and Port Lions. Each village local government (Tribal and/or City) was asked and agreed to participate in the survey. Approval from each household surveyed was received after permission was granted from their local governments. Households were defined as residences that were occupied at the time of the survey. At least 50% or more of the total households for each village was to be surveyed. In those villages which did not have 100% participation, survey results were expanded (multiplied) in order to estimate the total harvest for the entire village.

The Kodiak Refuge Subsistence Coordinator hired one individual from each village to conduct the surveys and complete migratory bird harvest survey forms. These Subsistence Migratory Bird Harvest Surveyors were in charge of the selection of households and the completion of each interview/survey form from each of the participating households. Payment was made for each household survey form completed and turned in. (Tables 1, 2)

Survey forms included the following information from each household: a listing of the migratory birds with the number of birds harvested and # of eggs gathered, the season or timing of harvesting or gathering, questions on the sharing and uses of the birds/eggs, and a map of hunting and gathering locations. Information was gathered for three time periods over the course of one year:

- Time Period 1 = Winter/Spring; March 11, 1999 - May 20, 1999
- Time Period 2 = Spring/Summer; May 21, 1999 - August 31, 1999
- Time Period 3 = Fall/Winter; September 1, 1999 - March 10, 2000

Table 1 Subsistence Migratory Bird Harvest Survey Population, Total Households, Number of Households Participating by Village, Kodiak Island, 1999 - 2000

VILLAGE	Total Population	Total Households (HH)	Total Households Participating	Percent Participating
Akhiok	78	35	22	63%
Karluk	46	13	10	77%
Larsen Bay	87	50	42	84%
Old Harbor	290	112	35	31%
Ouzinkie	259	82	55	67%
Port Lions	260	92	68	74%
TOTALS	1,020	384	232	Ave. = 66%

Table 2 Number of Households Which Caught Birds and /or Gathered Eggs, by Survey Period, Kodiak Island, 1999

VILLAGE	Tot. HH Participating	#HH Win/Spr Hunting & Gathering	%	#HH Spr/Sum Hunting & Gathering	%	#HH Fall/Win Hunting & Gathering	%
Akhiok	22	3	14	3	14	8	36
Karluk	10	0	0	0	0	7	70
Larsen Bay	42	0	0	0	0	10	24
Old Harbor	35	16	46	9	26	15	43
Ouzinkie	55	2	4	24	44	22	40
Port Lions	68	2	3	0	0	9	13
TOTALS	232	23	10	36	16	71	31

#HH = Number of Households
Win/Spr = Winter/Spring; **Spr/Sum** = Spring/Summer; **Fall/Win** = Fall/Winter

Although this study was based on a sound plan, it is important for the reader to be aware of factors that potentially affected the validity of the survey. The data is only as good as the information reported in the surveys. Any inaccuracies reported at the household level became exaggerated when the data were expanded. As a result, the information provided in this report can only be viewed as approximations of actual subsistence harvests. Confidence in the results will increase as surveyors and residents become more familiar with the survey design and as data from additional years are gathered.

RESULTS AND DISCUSSION:

Comprehensive Totals

The expanded estimated harvest of the six Kodiak Island villages from March 11, 1999 - March 10, 2000 was 6041 birds. The reported harvest from the surveys was 3893 birds. Mallards was the most frequently reported species harvested with 854 birds (22%) taken. An expanded total of 1740 eggs was estimated to be gathered during this time, and a reported harvest of 1147 eggs. Glaucous-wing gulls comprised 60% of the egg take. (Table 3,6,7)

Duck species accounted for 85.3% of migratory birds harvested followed by Canada geese with 6.5% of the total harvest, and ptarmigan with 6.1% of the total harvest. The only identified seabird species harvested was auklets (sea quail) accounting for 1.1% of total harvest.

Glaucous-wing, and mew gull eggs accounted for 92.5% of all eggs harvested with black-legged kittiwakes (4.5%), and tufted puffins (3%) the only other species whose eggs were harvested. Duck eggs was not reported taken for food in the Kodiak Island villages.

Converting estimates to useable weights, the expanded subsistence harvest of birds provided approximately 9371 pounds of food to Kodiak Island village residents. Of the total food harvested, 7431pounds (79%) was ducks, 1557 pounds (16%) geese, 321 pounds (4%) ptarmigan, and 62 pounds (1%) other seabirds. (Wentworth 1998)

Converting harvest estimates to useable weights, the expanded subsistence harvest of eggs provided approximately 174 pounds of food to the Kodiak Island village residents. Of the eggs gathered, 165 pounds (95%) was from gulls, 7 pounds (4%) kittiwakes, and a pound of tufted puffin eggs. Glaucous-winged gull eggs contributed 114 pounds (65%) of the total estimated useable weight of eggs, followed by mew gulls with 51 pounds (30%).

Seasonal Harvest Differences (Figure 2)

Fall/Winter (September 1 - March 10) was the survey period in which most Kodiak Island villages harvested birds, while the Spring/Summer (May 21 - August 31) was the primary period for gathering eggs. Approximately 89% of the 232 households surveyed reported harvesting birds during the Fall/Winter period. The Spring/Summer survey period was the primary egg

gathering period with 88% of the households participating in egg gathering activities during this time. Most migratory bird harvest occurred in the month of December, followed by March.

Table 3 Migratory Bird Reported Subsistence Bird Harvest and Egg Gathering by Survey Period, For the Six Kodiak Island Villages, 1999-2000.

SPECIES	March- May<br>**SPR. BIRDS 3/11 - 5/20/99**	**SPR. EGGS**	May - August<br>**SUM. BIRDS 5/21 - 8/31**	**SUM. EGGS**	September-March<br>**WIN. BIRDS 9/1 - 3/10/00**	**WIN. EGGS**	**TOT. BIRDS**	**TOT. EGGS**
**Canada Geese**	27	0	0	0	211	0	238	0
**Black Brant**	2	0	0	0	6	0	8	0
**TOTAL GEESE**	29	0	0	0	217	0	246	0
**Green-winged Teals**	0	0	0	0	110	0	110	0
**American Widgeons**	50	0	0	0	184	0	234	0
**Barrow's Goldeneyes**	55	0	0	0	464	0	519	0
**Black Scoters**	131	0	10	0	503	0	644	0
**Surf Scoters**	22	0	0	0	31	0	53	0
**White-winged Scoters**	28	0	0	0	103	0	131	0
**Gadwalls**	42	0	0	0	195	0	237	0
**Greater Scaup**	27	0	0	0	113	0	140	0
**Harlequin Ducks**	25	0	0	0	57	0	82	0
**King Eiders**	0	0	0	0	18	0	18	0
**Buffleheads**	36	0	0	0	126	0	162	0
**Common Mergansers**	0	0	0	0	38	0	38	0
**Mallards**	131	0	6	0	717	0	854	0
**Northern Pintails**	22	0	0	0	87	0	109	0
**Long-tailed Ducks**	0	0	0	0	41	0	41	0
**TOTAL DUCKS**	569	0	16	0	2,787	0	3,372	0
**Black-legged Kittiwake**	0	0	0	12	0	40	0	52
**Glaucous-winged Gulls**	0	246	0	195	0	252	0	693
**Mew Gulls**	0	0	0	368	0	20	0	388

**Auklets (Sea Quail)**	0	0	0	0	40	0	40	0
**Tufted Puffins**	0	0	0	14	0	0	0	14
**Unidentified Seabirds**	0	0	0	0	10	0	10	0
**TOTAL SEABIRDS**	0	246	0	589	50	312	50	1,147
**Ptarmigan**	50	0	0	0	175	0	225	0
**TOTALS**	**648**	**246**	**16**	**589**	**3,229**	**312**	**3,893**	**1,147**

A total of 31% of the households surveyed harvested migratory birds while 18 % gathered eggs. Ouzinkie (43%), Old Harbor (42%), and Akhiok (41%) had the highest percentage of hunters from households surveyed who actively hunted. Ouzinkie (49%) and Old Harbor (29%) had the highest percent of egg gatherers for those households surveyed. Larsen Bay and Karluk did not report gathering any eggs for this year. The majority of egg gathering occurred in the month of June.

The fall/winter survey period was the most important for migratory bird subsistence harvest with approximately 83% of the birds reported harvested during this time(n=3229). The spring period accounted for16% (n=648) of the birds harvested, while the summer period accounted for only 1% (n=16). The seven month fall/winter survey period is during the legal hunting season. Approximately 99% of the geese, 82% of the ducks, and 77% of the ptarmigan were harvested in fall/winter time period. Mallards and black scoters were harvested in the highest amounts for all seasons. For the fall/winter period 717 mallards, 503 black scoters, and 464 Barrow's goldeneyes were reported harvested. In terms of useable weight, the fall/winter survey period supplied the most food, accounting for a reported 4942 pounds followed by the winter/spring period with 1041 pounds, and the spring/summer with 27 pounds. Ducks contributed a total of 4768 pounds of meat followed by geese with 967 pounds, ptarmigan 225 pounds, and seabirds with 50 pounds.

The seasonal egg harvest patterns corresponded with the annual breeding cycles of gulls and kittiwakes found around Kodiak Island. In 1999, 73% (n=835 reported) of the eggs were harvested in the spring/summer survey periods with 27% (n=312) harvested during the fall/winter period. A total of 115 pounds of eggs was collected with 84 pounds gathered during the spring/summer survey period and 31 pounds gathered during the fall winter. Gull eggs accounted for 109 pounds of harvest with five pounds for kittiwakes and one pound for tufted puffins.

<u>Regional Harvest Differences</u>

The Village of Old Harbor harvested more migratory birds then all the other villages put together, with a expanded harvest of 4164 birds. Ouzinkie with 622 birds was followed closely by Port Lions with 573 birds harvested. Akhiok had 388 birds, Larsen Bay 233, and Karluk with 61 birds was harvested for the 1999/2000 survey year. Old Harbor also led all other villages with 17 species taken, followed by Port Lions with 14 species, and Ouzinkie with 9 species. (Tables 4,6, 7)

The reported duck harvest ranged from 46 birds in Karluk to 2223 birds in Old Harbor. Mallards and Barrow's goldeneyes were the only ducks taken from all six villages. Mallards were taken in

higher numbers in the villages of Akhiok, Karluk, and Ouzinkie. Barrow's goldeneyes were favored in Port Lions and Larsen Bay, while black scoters were most taken by Old Harbor followed closely by mallards. Other commonly taken species included bufflehead, greater scaup, green-winged teal, harlequin ducks, white-winged scoters, and surf scoters. King eiders were taken only in Port Lions and Ouzinkie areas, while Old Harbor was the only village to harvest substantial numbers of gadwall and American widgeon.
Old Harbor reported a harvest of 200 Canada geese, while Port Lions harvested a total of 46 geese. A total of six black brant were reported harvested in Port Lions and two harvested in Old Harbor.

Table 4 Migratory Bird Expanded Subsistence Bird Harvest by Village on Kodiak Island, 1999/2000. Annual

SPECIES	Akhiok	Karluk	Larsen Bay	Old Harbor	Ouzinkie	Port Lions	Total
**Canada Geese**	0	0	0	335	0	50	**385**
**Black Brant**	0	0	0	3	0	8	**11**
**TOTAL GEESE**	0	0	0	338	0	58	**396**
**Green-winged Teals**	0	0	0	135	23	16	**174**
**American Widgeons**	0	0	0	395	0	0	**395**
**Barrow's Goldeneyes**	19	25	130	402	110	98	**784**
**Black Scoters**	41	0	0	886	37	78	**1,042**
**Surf Scoters**	0	0	0	68	5	13	**86**
**White-winged Scoters**	0	0	0	108	15	71	**194**
**Gadwalls**	0	0	0	401	0	0	**401**
**Greater Scaup**	1	0	0	122	69	19	**211**
**Harlequin Ducks**	0	0	9	44	64	0	**117**
**King Eiders**	0	0	0	0	9	14	**23**
**Buffleheads**	23	0	2	162	48	14	**249**
**Common Mergansers**	0	0	0	44	0	15	**59**
**Mallards**	55	32	80	874	181	83	**1,305**
**Northern Pintails**	0	0	0	107	61	0	**168**
**Long-tailed Ducks**	0	0	0	10	0	44	**54**
**TOTAL DUCKS**	139	57	221	3,758	622	465	**5,262**

	Akhiok	Karluk	Larsen Bay	Old Harbor	Ouzinkie	Port Lions	
**Black-legged Kittiwake**	0	0	0	0	0	0	**0**
**Glaucous-winged Gulls**	0	0	0	0	0	0	**0**
**Mew Gulls**	0	0	0	0	0	0	**0**
**Auklets (Sea Quail)**	0	0	0	0	0	50	**50**
**Tufted Puffins**	0	0	0	0	0	0	**0**
**Unidentified Seabirds**	0	0	12	0	0	0	**12**
**TOTAL SEABIRDS**	0	0	12	0	0	50	**62**
**Ptarmigan**	249	4	0	68	0	0	**321**
**TOTALS**	**388**	**61**	**233**	**4,164**	**622**	**573**	**6,041**

Table 5 Expanded Subsistence Migratory Bird Egg Harvest for Kodiak Island Villages 1999-2000

SPECIES	Akhiok	Karluk	Larsen Bay	Old Harbor	Ouzinkie	Port Lions	Total Eggs
**Black-legged Kittiwake**	0	0	0	20	0	50	**70**
**Glaucous-winged Gulls**	53	0	0	1,021	0	63	**1,137**
**Mew Gulls**	0	0	0	0	489	25	**514**
**Auklets (Sea Quail)**	0	0	0	0	0	0	**0**
**Tufted Puffins**	19	0	0	0	0	0	**19**
**Total Eggs Harvested**	**72**	**0**	**0**	**1,041**	**489**	**138**	**1,740**

Ptarmigan was primarily harvested in Akhiok with 182 birds reported harvested, followed by Old Harbor with 40 harvested, and three in Karluk. The only other birds harvested was auklets (sea quail) from Port Lions and unidentified seabirds in Larsen Bay. (Figures 3,4) (Tables 4,6,7)

The villages of Old Harbor and Ouzinkie harvested the most eggs with 54% and 32% respectively of the total reported egg harvest. Karluk and Larsen Bay did not report any egg gathering activities. In Old Harbor, Glaucous-winged gulls eggs was the primary species gathered while mew gull eggs was the sole source of eggs gathered in Ouzinkie. Port Lions gathered the most species of eggs and including black-legged kittiwakes, Glaucous-winged gulls, and mew gulls. Akhiok harvested tufted puffin eggs as well as Glaucous-winged gull eggs. Glaucous-winged gull eggs showed up in the harvest of three of the four villages and had the highest number of eggs gathered for this survey year. (Figure 5) (Table 5)

Birds and Eggs Harvested Per Household

An average of eight birds were reportedly taken per Kodiak Island village household with an expanded average of 12.1 birds per household. The expanded harvest range was 5-6 for Karluk and Larsen Bay respectively, to 37 per household for Old Harbor. The expanded Old Harbor take for ducks and geese per household (n=34 and n=3 respectfully) was higher than all other villages combined. Akhiok takes the most ptarmigan per household (n=7), with only Old Harbor (n=0.6) and Karluk (n=0.3) also harvesting ptarmigan. Port Lions and Larsen Bay were the only Villages to report the harvest of seabirds with Port Lions harvesting one auklet per household and Larsen Bay harvesting one unidentified seabird per household. (Figure 6) (Table 6)

Old Harbor had an expanded harvest of 60 pounds of migratory bird food per household followed by 13 pounds for Akhiok, 11 pounds for Port Lions, 10 pounds for Ouzinkie, and 6 pounds each for Karluk and Larsen Bay. Old Harbor harvested 14 pounds of black scoters per household followed by 12 pounds for Canada geese, and 11 for mallards. Port Lions harvested 2 pounds of Canada geese per household followed by white-winged scoter, black scoter and Barrow's goldeneye with less than 2 pounds per household. Ouzinkie harvested 3 pounds of mallards per household. In Larsen Bay 3 pounds of Barrow's goldeneye were harvested per household, and in Karluk 3.5 pounds of mallards and 2 pounds Barrow's goldeneyes were harvested. In Akhiok 7 pounds of ptarmigan per household was harvested, along with two or more pounds from mallard and black scoters.

The average number of eggs reported harvested for all households was 3 with a range of zero eggs in Karluk and Larsen Bay to 6 eggs per household gathered for Old Harbor. (Figure 6) In Old Harbor 5 of those eggs came from glaucous-winged gulls and one from black-legged kittiwakes. Ouzinkie harvested 5 eggs per household (all mew gull eggs), with Akhiok harvesting two (both glaucous-winged gulls), and Port Lions harvesting one egg. All households reported harvest of less than one pound of eggs harvested per household.

Other Migratory Bird Harvest Survey Questions, Answered From Villages

The following questions were asked of all households surveyed, with the following results:

- "Do you feel bird numbers have changed around your area since you began hunting?"

Yes More = 3%; Yes Less = 29%; No Change = 12%; Did Not Know = 56%

Comments/Reasons included:

- Increased hunting competition from outside of the village
- Birds are being overharvested
- Effects from the oil spill
- Bird collisions with fishing boats with bright crab lights

- "Did your household harvest more, less, or about the same amount of birds or eggs as in the past?"

More = 11%; Less = 42%; The Same = 47%

Comments/Reasons included:

- Less or no eggs gathered due to late spring weather in 1999
- Age or health prevented harvest or gathering activities
- Increased competition

-The average percent of households which reported **sharing** migratory bird resources for all villages was 21%, with Old Harbor reporting 49% of their households sharing bird resources. On average a total of 29% of all households surveyed **received** migratory bird resources with households in Old Harbor and Akhiok receiving the most bird and eggs. For all Villages a total of 959 birds was reported shared along with 283 eggs.

The **total number of reported hunters was 86** and they spent 5197 hours afield for a total of 60 hours hunting per hunter. A **total of 86 people gathered eggs** for all Villages, and spent 325 hours or 4 hours of egg collection for each egg gatherer. (Table 7)

General Comments

Some general comments which came from survey respondents included:

- There are still concerns for the effects from the Exxon Valdez oil spill on waterfowl population numbers and availability for subsistence hunting. The numbers appear to be decreasing.
- Health issues have prompted elders in some communities to rely on other family members for subsistence waterfowl harvesting and or egg gathering activities.
- Work related problems included too busy to hunt or hunting more when there is less work available. -
One person commented that they do not have a skiff to take out for subsistence waterfowl hunting.
- Some people would like to see more law enforcement activities to keep sport hunters from over harvesting of waterfowl.
- One hunter commented that the present waterfowl hunting season should be extended by two weeks.
- A legal goose hunt was requested from hunters in Old Harbor.

CONCLUSIONS

Of the Kodiak Island households surveyed, as many as 32% participated in migratory bird subsistence harvest activities from 1999-2000. The bulk of migratory bird hunting activities occurred during the fall/winter seasons with lesser amounts occurring in spring. Egg gathering activities occurred mostly within the spring/summer season. An expanded total of 6041 birds was harvested and 1740 eggs gathered

Egg gathering activities during the spring of 1999 was probably severely effect by the unusually late spring weather. Kodiak's snow covered and frozen lakes, rivers, and other wetland areas experienced much later than usual nesting season for waterfowl and gulls. This late nesting season was the primary reason why no eggs were harvested in the village of Larsen Bay. This severe weather in spring of 1999 may have effected the overall waterfowl harvest in the fall and winter of 1999/2000. The weather in the spring of 2000 was much milder than 1999, and may mean a higher migratory bird harvest for the fall and winter of 2000/2001.

Old Harbor was the most active Kodiak Island village in the harvest of migratory bird resources with 63% of the birds harvested and 54% of the eggs gathered. Ouzinkie and Port Lions both harvested 12% of the birds while Ouzinkie gathered 32% of the eggs. Ducks were the primary migratory bird harvested for all villages with regional differences accounting for variation in species and numbers harvested. Mallards, black scoters, and Barrow's goldeneyes were the duck species most harvested (respectively) by all villages accounting for 60% of the duck species harvested. Akhiok with plenty of upland habitat for ptarmigan harvested this bird to a higher degree than any other village. A flock of Canada geese whose origin is not known has been arriving in the Old Harbor area during the winter and spring for the last 5-10 years. These geese have become a part of the subsistence migratory bird harvest cycle in Old Harbor, which is the only village reporting the major use of a goose resource. Port Lions and Larsen Bay were the only villages to report harvest of identified or unidentified seabirds.

Each Kodiak Island Village household was estimated to have an expanded harvest 24 pounds of food from migratory birds, with each household averaging three eggs gathered. Kodiak Island subsistence harvest of migratory bird resources continues to be a tradition based on winter to early spring harvest of birds and late spring to early summer harvest of gull eggs.

ACKNOWLEDGMENTS

A very special thank you to all the Kodiak Island Villages and households who took the time to answer questions on their subsistence migratory bird harvest activities. Without their cooperation this survey

could not be completed. A special thank you and job well done to the following Migratory Bird Harvest Surveyors who conducted the surveys: Mitch Simeonoff (Akhiok), Betty and Ronnie Lind (Karluk), Valen Norell (Larsen Bay), Jeff Peterson (Old Harbor), Ken Anderson (Ouzinkie), and Brian Kewan (Port Lions). I would like to acknowledge the help and guidance from the U.S. Fish and Wildlife Service's Migratory Bird office in financing the study and review of this report.

LITERATURE CITED

Kodiak Island Borough Population Figures for 1998, and personnel communications with each Kodiak Island City or Tribal Council Offices.

Mishler,C. 1992. 1991 Kodiak Road System Subsistence Harvest Survey. 59pp. Alaska Department of Fish and Game, Division of Subsistence. FWS Cooperative Agreement No. 14-16-007-91-7754 and ADF&G Agreement No. COOP-92-012.

Seim,S., and C. Wentworth. 1996. Subsistence Migratory Bird Harvest Survey, Bristol Bay, 1995. 57pp. U.S. Fish and Wildlife Service, Migratory Bird Management, Anchorage, Alaska.

Wentworth,C. 1998. Subsistence Waterfowl Harvest Survey, Yukon-Kuskokwim Delta, Comprehensive Report 1987-1997. 176pp. U.S. Fish and Wildlife Service, Migratory Bird Management, Anchorage, Alaska.

Figure 2: Percent of Households Harvesting Migratory Birds and/or Gathering Eggs by Village and Seasons, 1999/2000

Figures 3 and 4: Total Reported Birds Harvested = **3,893**; Total Pounds of Food = **6,007**

Figure 5:

Figure 6 Number of Reported Birds and Eggs Harvested Per Household by KodiakIslandVillages,1999/2000.

Table 7 Summary of Expanded Subsistence Bird and Egg Harvest per Household for the

Kodiak Island Villages. 1999-2000

Kodiak Is. Villages	Total Birds	Total Eggs	Total HH	Birds/ HH	Eggs/ HH	Total Pounds Bird	Pounds of Birds/ HH
Akhiok	388	72	35	11.08	2.06	443	12.66
Karluk	61	0	13	5.69	0	82	6.31
Larsen Bay	233	0	50	4.66	0	305	6.1
Old Harbor	4,164	1,041	112	37.17	9.3	6,705	59.87
Ouzinkie	622	489	82	7.58	5.96	824	10.05
Port Lions	573	138	92	6.23	1.5	1,012	11
Kodiak Is. Totals	6,041	1,740	384	15.73	4.53	9,371	24.4

HH = Number of Households

Table 8 Migratory Bird Reported Subsistence Bird Harvest by Village on Kodiak Island, 1999/2000.

SPECIES	Akhiok	Karluk	Larsen Bay	Old Harbor	Ouzinkie	Port Lions	Total
Canada Geese	0	0	0	198	0	40	238
Black Brant	0	0	0	2	0	6	8
TOTAL GEESE	0	0	0	200	0	46	246
Green-winged Teals	0	0	0	80	17	13	110
American Widgeons	0	0	0	234	0	0	234
Barrow's Goldeneyes	14	20	104	238	65	78	519
Black Scoters	30	0	0	524	28	62	644

**Surf Scoters**	0	0	0	40	3	10	**53**
**White-winged Scoters**	0	0	0	64	11	56	**131**
**Gadwalls**	0	0	0	237	0	0	**237**
**Greater Scaup**	1	0	0	72	52	15	**140**
**Harlequin Ducks**	0	0	8	26	48	0	**82**
**King Eiders**	0	0	0	0	7	11	**18**
**Buffleheads**	17	0	2	96	36	11	**162**
**Common Mergansers**	0	0	0	26	0	12	**38**
**Mallards**	40	26	69	517	136	66	**854**
**Northern Pintails**	0	0	0	63	46	0	**109**
**Long-tailed Ducks**	0	0	0	6	0	35	**41**
**TOTAL DUCKS**	102	46	183	2223	449	369	**3372**
**Black-legged Kittiwake**	0	0	0	0	0	0	**0**
**Glaucous-winged Gulls**	0	0	0	0	0	0	**0**
**Mew Gulls**	0	0	0	0	0	0	**0**
**Auklets (Sea Quail)**	0	0	0	0	0	40	**40**
**Tufted Puffins**	0	0	0	0	0	0	**0**
**Unidentified Seabirds**	0	0	10	0	0	0	**10**
**TOTAL SEABIRDS**	0	0	10	0	0	40	**50**
**Ptarmigan**	182	3	0	40	0	0	**225**
**TOTALS**	**284**	**49**	**193**	**2463**	**449**	**455**	**3893**

Species	Spr. Birds	Spr. Eggs	Expanded (13/35)	Sum. Birds	Sum. Eggs	Expanded (14/35)	Win. Birds	Win. Eggs	Expanded (12/35)	Expanded Total	Orig Report	
Canada Goose			0						0	***0***		
Black Brant			0						0	***0***		
Green-winged Teal			0						0	***0***		
American Widgeon			0						0	***0***		
Goldeneye			0				14		41	***41***	19	
Black Scoter	7		19				23		67	***86***	41	
Surf Scoter			0						0	***0***		
White-winged Scoter			0						0	***0***		
Gadwall			0						0	***0***		
Greater Scaup			0				1		3	***3***	1	
Harlequin			0						0	***0***		
King Eider			0						0	***0***		
Bufflehead			0				17		50	***50***	23	
Common Merganser			0						0	***0***		
Mallard	22		59				18		53	***112***	55	
Northern Pintail			0						0	***0***		
Long-tailed Duck												
Black-legged Kittiwake			0						0	***0***		
Glaucous-winged Gull			0		42				0	***0***		
Mew Gull			0						0	***0***		
Auklet			0						0	***0***		
Tufted puffin			0						0	***0***		
Herring Gull												
Unidentified Seabirds			0		14				0	***0***		
Ptarmigan	10		27				172		502	***529***	249	
**TOTAL BIRDS HARVESTED**	**39**		**105**	**0**		**0**	**245**		**715**	***820***	**388**	
**Total Eggs Harvested**		**0**	**0**		**56**	**77**		**0**	**0**	***133***		

Note: The number of houses surveyed was 13, 14, and 12 respectively; not 22 for each season. This greatly increases the total number of birds harvested.

Species	Spr. Birds	Spr. Eggs	Expanded (10/13)	Sum. Birds	Sum. Eggs	Expanded (10/13)	Win. Birds	Win. Eggs	Expanded (7/13)	Expanded Total	From Orig Report	
Canada Goose									**0**	***0***		
Black Brant									**0**	***0***		
Green-winged Teal									**0**	***0***		
American Widgeon									**0**	***0***		
Goldeneye							19		**35**	***35***	25	
Black Scoter									**0**	***0***		
Surf Scoter									**0**	***0***		
White-winged Scoter									**0**	***0***		
Gadwall									**0**	***0***		
Greater Scaup									**0**	***0***		
Harlequin									**0**	***0***		
King Eider									**0**	***0***		
Bufflehead									**0**	***0***		
Common Merganser									**0**	***0***		
Mallard							26		**48**	***48***	32	
Northern Pintail									**0**	***0***		
Long-tailed Duck												
Black-legged Kittiwake									**0**	***0***		
Glaucous-winged Gull									**0**	***0***		
Mew Gull									**0**	***0***		
Auklet									**0**	***0***		
Tufted puffin									**0**	***0***		
Herring Gull												
Unidentified Seabirds									**0**	***0***		
Ptarmigan							3		**6**	***6***	4	
**TOTAL BIRDS HARVESTED**	**0**		**0**	**0**		**0**	**48**		**89**	***89***	**61**	
**Total Eggs Harvested**		**0**	**0**		**0**	**0**		**0**	**0**	**0**		

Species	Spr. Birds	Spr. Eggs	Expanded (39/50)	Sum. Birds	Sum. Eggs	Expanded (39/50)	Win. Birds	Win. Eggs	Expanded (25/50)	Expanded Total	From Orig Report	
Canada Goose									**0**	***0***		
Black Brant									**0**	***0***		
Green-winged Teal									**0**	***0***		
American Widgeon									**0**	***0***		
Goldeneye							108		**216**	***216***	130	
Black Scoter									**0**	***0***		
Surf Scoter									**0**	***0***		
White-winged Scoter									**0**	***0***		
Gadwall									**0**	***0***		
Greater Scaup									**0**	***0***		
Harlequin							13		**26**	***26***	9	
King Eider									**0**	***0***		
Bufflehead							2		**4**	***4***	2	
Common Merganser									**0**	***0***		
Mallard							69		**138**	***138***	80	
Northern Pintail									**0**	***0***		
Long-tailed Duck												
Black-legged Kittiwake									**0**	***0***		
Glaucous-winged Gull									**0**	***0***		
Mew Gull									**0**	***0***		
Auklet									**0**	***0***		
Tufted puffin									**0**	***0***		
Herring Gull												
Unidentified Seabirds							10		**20**	***20***	12	
Ptarmigan									**0**	***0***		
**TOTAL BIRDS HARVESTED**	**0**		**0**	**0**		**0**	**202**		**404**	***404***	**233**	
**Total Eggs Harvested**		**0**	**0**		**0**	**0**		**0**	**0**	**0**		

Note: The number of houses surveyed was 39, 39, and 25 respectively; not 42 for each season. This greatly increases the total number of birds harvested.

Species	Spr. Birds	Spr. Eggs	Expanded (55/82)	Sum. Birds	Sum. Eggs	Expanded (55/82)	Win. Birds	Win. Eggs	Expanded (55/82)	Expanded Total	From Orig Report	
Canada Goose		0	**0**		0	**0**			**0**	***0***		
Black Brant			**0**			**0**			**0**	***0***		
Green-winged Teal			**0**			**0**	8		**12**	***12***	23	
American Widgeon			**0**			**0**			**0**	***0***		
Goldeneye	6		**9**			**0**	59		**88**	***97***	110	
Black Scoter	2		**3**			**0**	26		**39**	***42***	37	
Surf Scoter			**0**			**0**	3		**4**	***4***	5	
White-winged Scoter			**0**			**0**	11		**16**	***16***	15	
Gadwall			**0**			**0**			**0**	***0***		
Greater Scaup	1		**1**			**0**	51		**76**	***78***	69	
Harlequin	2		**3**			**0**	46		**69**	***72***	64	
King Eider			**0**			**0**	7		**10**	***10***	9	
Bufflehead	2		**3**			**0**	34		**51**	***54***	48	
Common Merganser			**0**			**0**			**0**	***0***		
Mallard	6		**9**			**0**	223		**332**	***341***	181	
Northern Pintail	2		**3**			**0**	44		**66**	***69***	61	
Long-tailed Duck			**0**			**0**			**0**			
Black-legged Kittiwake			**0**						**0**	***0***		
Glaucous-winged Gull			**0**						**0**	***0***		
Mew Gull			**0**		349	**0**			**0**	***0***		
Auklet			**0**			**0**			**0**	***0***		
Tufted puffin			**0**			**0**			**0**	***0***		
Herring Gull			**0**		27	**0**			**0**	***0***		
Unidentified Seabirds			**0**			**0**			**0**	***0***		
Ptarmigan												
**TOTAL BIRDS HARVESTED**	**21**		**31**	**0**		**0**	**512**		**763**	**795**	**622**	
**TOTAL EGGS HARVESTED**		**0**	**0**		**376**	**561**		**0**	**0**	**561**		

Species	Spr. Birds	Spr. Eggs	Expanded (68/92)	Sum. Bird	Sum. Egg	Expanded (68/92)	Win. Birds	Win. Eggs	Expanded (68/92)	Expanded Total	From Orig Report	
Canada Goose			**0**			**0**	40		**54**	***54***	50	
Black Brant			**0**			**0**	6		**8**	***8***	8	
Green-winged Teal			**0**			**0**	13		**18**	***18***	16	
American Widgeon			**0**			**0**			**0**	***0***	0	
Goldeneye			**0**			**0**	71		**96**	***96***	98	
Black Scoter			**0**			**0**	62		**84**	***84***	78	
Surf Scoter			**0**			**0**	10		**14**	***14***	13	
White-winged Scoter			**0**			**0**	46		**62**	***62***	71	
Gadwall			**0**			**0**			**0**	***0***	0	
Greater Scaup			**0**			**0**	15		**20**	***20***	19	
Harlequin			**0**			**0**			**0**	***0***	0	
King Eider			**0**			**0**	11		**15**	***15***	14	
Bufflehead			**0**			**0**	11		**15**	***15***	14	
Common Merganser			**0**			**0**	12		**16**	***16***	15	
Mallard			**0**			**0**	58		**78**	***78***	83	
Northern Pintail			**0**			**0**			**0**	***0***	0	
Long-tailed Duck			**0**			**0**	35		**47**	***47***	44	
Black-legged Kittiwake			**0**			**0**			**0**	***0***		
Glaucous-winged Gull			**0**			**0**			**0**	***0***		
Mew Gull			**0**			**0**			**0**	***0***		
Auklet			**0**			**0**	40		**54**	***54***	50	
Tufted puffin			**0**			**0**			**0**	***0***		
Herring Gull			**0**			**0**			**0**	***0***		
Unidentified Seabirds			**0**			**0**	0		**0**	***0***		
Ptarmigan			**0**			**0**				***0***		
**TOTAL BIRDS HARVESTED**	**0**		**0**	**0**		**0**	**430**		**582**	**582**	**573**	
**Total Eggs Harvested**		**0**	**0**		**0**	**0**		**0**		**0**		
