# Supplementary material for: Harvest of waterfowl and Sandhill Crane in rural Alaska: Geographic and seasonal patterns
Source: PLoS One. 2024 Jul 25;19(7):e0307135. doi: 10.1371/journal.pone.0307135 (PMC11271962; doi:10.1371/journal.pone.0307135)
Supplement: S3 File — (PDF) [file pone.0307135.s003.pdf]

Koyukuk/Nowitna National Wildlife Refuge Complex

Subsistence Waterfowl Harvest Survey
Galena, Huslia, Nulato, Koyukuk, Kaltag, Hughes, Ruby
1998 and 1999

Key Words: Waterfowl, harvest, subsistence, spring hunting, household survey, Koyukon

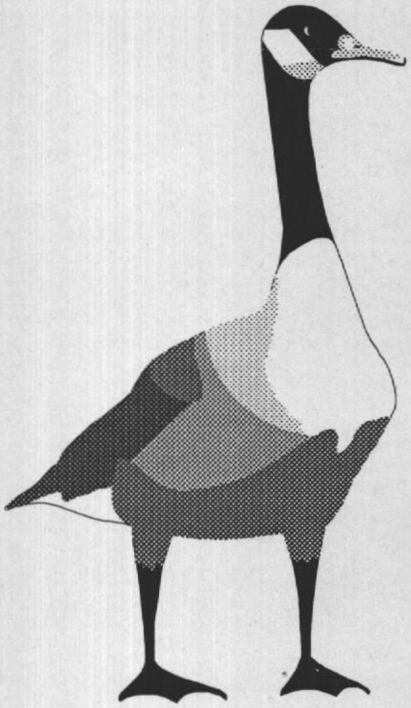

Deborah D. Webb
fsddw@uaf.edu

U.S. Fish and Wildlife Service
Koyukuk/Nowitna National Wildlife Refuge Complex
P.O. Box 287
Galena, AK 99741

Final Report FY00-01
May 23, 2000

Table of Contents

Table of Contents ii

List of Figures iii

List of Tables v

Abstract 1

Introduction 1

Methods 2

Results and Discussion 3

- *Survey participation 1998 and 1999* 3
- *Subsistence Waterfowl Harvest 1998 and 1999* 3
- *Species take in 1998 and 1999* 4
- *Harvest comparison to prior surveys in the same region* 6
- *Hunters' perceptions of waterfowl population trends in 1998* 6
- *Egg harvests 1998 and 1999* 7
- *Comparison of Harvests in 1998 and 1999 by Communities* 7
- *Comparison between Spring Harvest and Summer-Fall Harvest 1998 and 1999* 9
- *Participation in waterfowl hunting and use in 1998 and 1999* 10
- *Efforts in hunting waterfowl in 1998 and 1999* 11
- *Were 1998 and 1999 'good' or 'bad' waterfowl hunting years?* 12
- *Problems and Recommendations* 12

Conclusions 13

Acknowledgements 14

Literature cited 14

Appendix I. Table 1. Usable weight of bird species harvested in the Koyukon region 35

Appendix II. Survey materials 36

List of Figures

Fig. 1. Estimated number of waterfowl harvested by seven communities in the Koyukon region in 1998 and 1999 based on household interviews 17

Fig. 2. Estimated pounds of edible waterfowl meat harvested by seven communities in the Koyukon region in 1998 and in 1999 based on household interviews 17

Fig. 3. Estimated number of waterfowl harvested (by species) in seven communities of the Koyukon region in 1998 and in 1999, based on household interviews 18

Fig. 4. Years spent waterfowl hunting in the community in relation to perceived trend in waterfowl numbers, based on interviews in 293 households of seven communities in the Koyukon region, spring 1998 18

Fig. 5. Hunter comments on changes in population size of different types of waterfowl obtained during household interviews of seven communities in the Koyukon region, spring 1998 19

Fig. 6. Seasonality of goose and duck harvests in seven communities in the Koyukon region in 1998 and in 1999 19

Fig. 7. Waterfowl harvest in numbers of birds (top) and in pounds of edible meat (bottom) in seven communities in the Koyukon region in 1998, based on household interviews 20

Fig. 8. Waterfowl harvest in numbers of birds (top) and in pounds of edible meat (bottom) in seven communities in the Koyukon region in 1999, based on household interviews 20

Fig. 9. Household participation in waterfowl hunting based on surveys in seven villages in the Koyukon region, 1998 21

Fig. 10. Household participation in waterfowl hunting by seven villages in the Koyukon region, 1999 22

Fig. 11. Seasonality of waterfowl hunting reported by households in seven communities in the Koyukon region during 1998-1999 household surveys 23

List of Figures

Fig. 12. Size of waterfowl harvest relative to previous years as estimated by households in seven communities in the Koyukon region, 1998 24

Fig. 13. Size of waterfowl harvest relative to previous years as estimated by households in seven communities in the Koyukon region, 1999 25

5

List of Tables

Table 1. Participation in the subsistence waterfowl harvest survey in the Koyukon region in 1998 and in 1999, based on household interviews 26

Table 2. Waterfowl harvest by seven communities in the Koyukon region by species and by season, 1998, based on household interviews 27

Table 3. Waterfowl harvest by seven communities in the Koyukon region by species and by season, 1999, based on household interviews 28

Table 4. Rank of waterfowl species abundance compared to rank of species harvested in the Koyukon region 29

Table 5. Abundance of waterfowl species in the Koyukuk stratum (4,100 mi^2), including Koyukuk and Kanuti NWRs, based on annual aerial breeding population survey, USFWS, Migratory Birds, Juneau 30

Table 6. Comparison of 1998 and 1999 total waterfowl subsistence harvest with estimates from previous surveys conducted in the Koyukon region 31

Table 7. Estimated harvest of waterfowl per household and per capita by seven communities in the Koyukon region in 1998 and 1999, based on household interviews 32

Table 8. Number of households harvesting, sharing, receiving, and using (either harvesting, sharing or receiving) waterfowl in seven communities in the Koyukon region in 1998 and in 1999 33

Table 9. Distances from the community while hunting waterfowl and time spent hunting waterfowl by households of seven communities in the Koyukon region in 1998 and in 1999 34

Abstract

In 1998 and in 1999 the U.S. Fish and Wildlife Service in Galena conducted subsistence waterfowl harvest surveys in seven communities (Ruby, Galena, Koyukuk, Nulato, Kaltag, Huslia and Hughes) on and adjacent to the Koyukuk/Nowitna National Wildlife Refuge Complex. Household interviews were conducted in May and early June to estimate spring harvest, and in October to estimate summer and fall harvest. Harvest estimates varied considerably between years and seasons. In 1998, the overall annual subsistence waterfowl harvest was estimated at 2,733 birds; 64% of these birds were taken in the spring. In 1999, the overall annual subsistence waterfowl harvest was estimated at 2,064 birds; 75% of these birds were taken in the spring. In 1998, more ducks (59%) were harvested than geese (40%), while in 1999, more geese (57%) were harvested than ducks (43%). In terms of edible weight, geese dominated the subsistence harvest in both years. The reported waterfowl harvest represented an estimated total usable weight of 6,717 pounds in 1998, and 6,103 pounds in 1999. Species most commonly taken were Canada goose, white-fronted goose, mallard, American wigeon, and northern pintail. More households reported using waterfowl in the spring than in the fall. In 1998, 50% of interviewed households used waterfowl in the spring while 38% did so in the fall. In 1999, 61% of interviewed households reported spring waterfowl use while only 33% participated in the fall harvest. Total duck harvests in the study area in 1998 and 1999 were 38% and 67% lower than the estimates obtained by the same methods in 1992. Total goose harvest estimates were 45% and 39% lower than the respective 1992 estimates.

Introduction

The Koyukuk/Nowitna National Wildlife Refuge (NWR) Complex encompasses nearly 30,000 km^2 (11,500 mi^2) of western Interior Alaska. It contains three separate Refuges, the Koyukuk NWR, the Nowitna NWR and the northern unit of the Innoko NWR, locally known as Kaiyuh Flats. The Koyukuk NWR lies within the floodplain of the Koyukuk River and consists largely of wetlands combined with lowland forests. The Nowitna NWR is bisected by the Nowitna River and includes extensive lowland forests dotted with wetlands and forested hills capped by alpine tundra. The Kaiyuh Flats south of the Yukon River can be characterized as a wide lowland interlaced by sloughs, creeks, and lakes. In addition to valuable moose habitat, the wetlands within the Refuge Complex provide important staging and breeding grounds for at least 23 species of waterfowl. Annual estimates of duck production on the Koyukuk NWR and the Kaiyuh Flats ranged between 62,050 and 199,155 ducklings during 1983-1993 (Saperstein, 1997).

Comprising over 90% of the region's population, the Koyukon Athabascan people are indigenous to these refuges. They have always relied on the land's abundant fish and wildlife resources, of which waterfowl have made a significant contribution. Waterfowl hunting has traditionally been part of the annual subsistence harvest cycle and becomes especially important in the spring, when the birds' arrival provides fresh fowl and a welcome change in the diet. The importance of waterfowl and other birds in the culture of the Koyukon people is reflected by their detailed knowledge of birds, as documented by Nelson (1983).

Despite the establishment of refuges in the main breeding, migration, and wintering areas, white-fronted geese have concerned biologists because the population in Northwest and Interior Alaska has declined.

This regional decline has occurred while overall abundance of mid-continent white-fronted geese has increased (Spindler et al.1998). Moreover, demands on this resource may increase when spring hunting is legalized according to recent amendments to the 1918 Migratory Bird Treaty Act. Prior to regulating subsistence waterfowl hunting seasons in spring and summer in Alaska, the U.S. Fish and Wildlife Service (USFWS) must establish management bodies that also include Native and State representatives. The objectives of the present study were to determine which waterfowl species are harvested and to estimate the quantities utilized for subsistence in the Koyukon region. This information is needed for management bodies to recommend reasonable and sustainable seasons and bag limits for the subsistence harvest.

Methods

Systematic household interviews proved to be a viable method to assess subsistence waterfowl harvest patterns elsewhere in Alaska (ADF&G et al. 1994, Wentworth and Seim 1996, Kawerak Inc. et al. 1997, Maniilaq Assoc. et al. 1997). Therefore, the present study was modeled after these surveys. Household interviews were conducted in seven communities in and adjacent to the Koyukuk/Nowitna NWR Complex: Ruby, Galena, Koyukuk, Nulato, Kaltag, Huslia, and Hughes.

Surveys took place twice a year in 1998 and in 1999: In 1998, spring surveys were conducted in May and June to estimate harvests made between April 1 and May 20. Summer and fall harvest estimates for the period between May 20 and October 8 were based on surveys conducted in mid-to late October. In 1999, the surveyed period in the spring was extended from April 1 to June 1 because of a late breakup and a cold spring. Summer and fall surveys remained the same as in 1998.

Approval of the survey was obtained from each community's tribal council. Plans were also submitted to the city office of each community. Communities were assured that participation in the survey would be voluntary and anonymous, and would not result in any law enforcement actions. In 1998 we used village maps (sources: AVEC; Interior Telephone Co.; Gana-A' Yoo, Ltd.; K'oyitl'ots'ina, Ltd.; Ruby City Council) to identify households. Maps of each community were verified by local assistants and numbers were assigned to each house. In 1999, household lists were compiled and kept by local assistants. Houses that were unoccupied during the survey period were not included in further analyses.

In Ruby, Koyukuk, Nulato, Kaltag, Huslia, and Hughes our goal was to survey the entire community. Due to Galena's larger population we did not attempt to interview each household, but instead randomly sampled 50% of all households. This sample was used to represent total household use in Galena to allow comparison with other communities. A different random sample was obtained in each year of the survey.

Interviews were primarily conducted by a USFWS Refuge Information Technician (RIT) who is a long-time resident of the local area, and by the author, a USFWS Biological Science Technician with professional expertise in scientific sampling. A local resident from each community was contracted to assist the team. Galena was surveyed by a local resident who was contracted through Louden Tribal Council.

During the initial contact with each household, we attempted to identify the primary hunters or users of waterfowl. To help with bird identification during interviews, household members were presented with color pictures showing 34 different types of water birds (including illustrations of both sexes for ducks). The back of the identification sheet contained descriptions of the bird species along with bird names commonly used in the area, including Koyukon Athabascan names (Nelson, 1983). Some bird species that closely resemble each other were combined in groups (e.g., swan, scaup, goldeneye). Upland game birds (ptarmigan, grouse) were not included in the survey. The survey form contained a questionnaire about observed trends in waterfowl populations, hunting, and use of waterfowl (see Appendix II). The questionnaire incorporated suggestions made by the tribal councils and knowledgeable people from the area. Survey forms were slightly adjusted after the first survey. During each visit, households received a written explanation of the survey and harvest numbers reported in the previous survey.

Results and Discussion

Survey participation 1998 and 1999

The number of interviewed households ranged from 361 in the spring of 1998 to 403 in the fall of 1999. In Huslia, Nulato, Koyukuk, Kaltag, Hughes, and Ruby, where we attempted to include 100% of occupied households, we surveyed an average of 87% of households during the two years (Table 1). During every survey a small number of households, six on average, declined to participate. In some households members were out of town and could not be contacted while the surveys took place. For example, a number of people from Hughes were at a potlatch in a nearby community, causing low coverage during the spring survey in 1998. In 1999, one of the major waterfowl hunters in Koyukuk was out of town during the spring and fall surveys. However, according to local assistants in each community, we usually surveyed most of the primary subsistence hunters. In Galena, the method of randomly sampling 50% of households implied surveying a certain number of households (*see* Methods). Households that could not be contacted were therefore replaced with alternate random samples.

Subsistence Waterfowl Harvest 1998 and 1999

Reported harvest numbers of waterfowl by species and by community are shown in Table 2 (1998) and in Table 3 (1999).

In 1998, residents of seven villages on and adjacent to the Koyukuk/Nowitna NWR Complex harvested an estimated 2,733 water birds (harvest estimates of Galena were estimated from a 50% random sample, *see* Methods). Duck harvests outnumbered goose harvests; approximately 1,619 (59%) ducks were taken compared to 1,081 (40%) geese. Four swans and 24 cranes were reported, representing only a small proportion (1%) of the subsistence waterfowl take (Figs. 1, 3).

In 1999, waterfowl harvests declined by 25% relative to 1998. Nevertheless, goose harvests in 1999 outnumbered goose harvests in 1998; roughly 100 (10%) more geese were taken in 1999. The decline in harvest numbers in 1999 was entirely attributed to lower duck harvests: almost 50% fewer ducks

were taken in 1999 than in 1998. Goose harvests in 1999 outnumbered duck harvests; approximately 1,184 geese (57%) were taken compared to 877 ducks (43%). No swan harvests and only three crane harvests were reported in 1999 (Figs. 1, 3).

An analysis of the waterfowl use in terms of edible weight may be a more appropriate measure of resource importance for subsistence. Wentworth and Seim (1996) estimated that edible waterfowl weight comprises approximately 75% of the round weight. I converted numbers of harvested birds into pounds of edible meat using the values for each species reported in Wentworth and Seim (1996). For species not covered in their report, weight was calculated from round weight listed in Bellrose (1980) (*see* Apendix I).

After edible weight was analyzed, the importance ranking of ducks and geese shifted; in 1998, more than two thirds of the edible meat was provided by geese (4,265 pounds, 69%). Ducks contributed 1,859 pounds (28%) and swans, cranes and other or unidentified waterfowl provided 233 pounds (3%) (Fig. 2). In 1999, goose meat contributed more than 80% (5,041 pounds) to the harvest total, duck meat a little less than 20% (1042 pounds).

1998 harvests averaged 1.54 birds (3.8 lbs.) per resident (using population estimates from AK Dept. of Labor Research 1998). The average household harvest in 1998 was 5.3 birds (13 lbs.) per occupied household. The 1999 per capita and per household harvests were somewhat lower; per capita harvest averaged 1.14 birds (3.36 lbs.) (using population estimates from AK Dept. of Labor Research 1999), and average harvest per occupied household was 3.9 birds (11.53 lbs. edible meat).

Compared to surveys recently conducted elsewhere in Alaska, harvests in the present study were low. On the Yukon-Kuskokwim Delta household harvest was roughly seven times greater than in our surveyed region; from 1985 to 1995 the estimated annual average was 33 birds (95 lbs.) per household (Wentworth and Seim, 1996). Similarly, high household takes of birds were displayed in Shungnak in 1993/94 (Alaska Department of Fish and Game ADF&G et al. 1994) and in Buckland in 1996/97 (Maniilaq Association et al.1997). In Gambell and Savoonga on St. Lawrence Island the annual average in 1995/96 was 79 birds per household (calculated from Kawerak Inc. et al.1997). In other communities of the Bering Strait Region bird harvests (upland game birds included) averaged from 1.74 birds per person in Nome to15.62 birds per person in Diomede (Paige, et al. 1996). Wolfe et al. (1990) estimated the 1985 per capita harvest in the Upper Yukon-Koyukuk-Lower Tanana region at 8.2 birds (19.1 lbs.) per person (approx. five times greater than 1998 estimates). The region, which included 33 communities, was ranked second by number and weight of migratory birds harvested, among the 16 Alaskan regions studied. Wolfe et al. (1990) estimated statewide average of 2.8 birds (6.9 lbs.) harvested per rural resident in 1985.

Species take in 1998 and 1999

The 1998 harvest included 17 waterfowl species. The five top-ranking species were lesser Canada goose (24.9%), mallard (17.3%), American wigeon (14.4%), greater white-fronted goose (11.5%) and northern pintail (9.1%). In 1999, the harvest included 16 waterfowl species. The same five species were top-ranking in 1999 but in different order: Lesser Canada goose harvests were highest (27.8%),

followed by greater white-fronted goose (26.1%), mallard (14.1%), American wigeon (9.0%), and northern pintail (7.7%) (Fig. 3). Fewer than 10 each of swan, black brant, canvasback, goldeneye, ring-necked duck, and merganser were reported in each year (Fig. 2). Unidentified ducks averaged 13.4% and 18.8% of the total duck harvest in 1998 and 1999, respectively. No harvests were recorded of loons, grebes, shorebirds and gulls.

Wolfe et al. (1990) reported a total of 19 harvested species for the Upper Yukon-Koyukuk-Lower Tanana region in 1985. According to other investigations conducted in the 1980's, species taken most frequently in the area included Canada goose, mallard, northern pintail (Loranger 1985, Marcotte and Haynes 1985, Looman 1987, Schroeder 1987), white-fronted goose (Loranger 1985, Looman 1987, Schroeder 1987), American wigeon (Loranger 1985, Marcotte and Haynes 1985, Schroeder 1987), white-winged scoter, scaup, green-winged teal (Loranger 1985, Schroeder 1987), oldsquaw (Marcotte and Haynes 1985, Schroeder 1987) and common goldeneye (Schroeder 1987).

All species shown above were reported as harvested in this study, but the proportion of species harvested has varied over time. This may be a reflection of changes in relative abundance (Wolfe et al. 1990). The Division of Migratory Birds (USFWS) in Juneau has conducted aerial breeding pair surveys of ducks and geese in different parts of Alaska for several decades. Table 4 shows the ranking of duck species within harvests (based on number harvested) related to the abundance of the species in the Koyukuk stratum (including Koyukuk and Kanuti NWRs, *see* Table 5). American wigeon and northern pintail are the most abundant duck species in the area and were frequently taken in 1998 and 1999. In the present study, rank in harvest of waterfowl species was significantly correlated with their rank in abundance (1998 p=0.005, 1999 p=0.004, Spearman Rank corr. coeff.) (*see* Table 4). There were some exceptions, however. In both survey years the mallard, which ranked fifth among the most abundant species, was top-ranking among the species harvested. This might be due to a preference for larger species which provide more usable weight (Loranger 1985). Further, large species are probably an easier target than small ones. Scaup are one of the most common species as observed during duck production surveys conducted by the USFWS in Galena from 1983 to 1993 (Saperstein 1997), but surprisingly, they comprised only one percent of the harvest in 1998. In 1999 no scaups were harvested. This may be due to late arrival and limited accessability to areas of high scaup density, since scaup frequently nest around isolated lakes and in upland habitat. Harvests of oldsquaw and goldeneye comprised less than two percent of the annual harvest in 1998 and in 1999, although the species were frequently taken in the past (Marcotte and Haynes 1985, Schroeder 1987). Both species ranked low in the abundance chart (Table 4), and aerial breeding pair surveys conducted since 1957 indicate that both species have declined in Alaska in the past two decades (Hodges et al. 1996). Low swan and crane harvests are apparently not uncommon. None of the previously mentioned studies documented high swan or crane take in the Koyukon region. Snow goose and black brant are the only species harvested that do not breed in the Refuge area, and each were harvested in low numbers.

Harvests in 1999 were considerably lower than in 1998. The only species harvested in substantially higher numbers in 1999 was the greater white-fronted goose. The 1999 harvest of white-fronts exceeded the 1998 harvest by more than 40%. Harvest of white-fronts in 1999 was almost as high as harvest of Canada geese, whereas in 1998 the white-fronted goose harvest was about half that of Canada geese. The meat of white-fronted geese has traditionally been preferred to the meat of Canada geese by Koyukon Athabascan people in the region (Nelson 1983). Five communities (Nulato, Huslia, Koyukuk,

Kaltag, and Hughes) took more white-fronts in 1999 than in 1998. Households in Nulato accounted for the largest share of the 1999 white-front harvest among the communities (32%) and harvested more than four times as many white-fronts in 1999 than in 1998. Possible reasons include better travel conditions in the spring of 1999, compared to 1998, which allowed people from the downriver communities to cross the Yukon River and access the Kaiyuh Flats.

White-fronted geese have concerned biologists because the population in Interior-Northwest Alaska has declined, although the overall number of white-fronts in Alaska has increased (Hodges et al. 1996). Aerial surveys conducted by the USFWS in Galena in 1999 showed no change in numbers from 1998 (Spindler 1999, Spindler et al. 1999). Numbers of adult and young white-fronts counted during the 1999 float surveys (Dulbi River and Slough, Kanuti River) remained lower than in the 1980's, but increased slightly over the 1998 level. Perhaps locally higher white-front abundances combined with better access in 1999 led to the increase in harvest numbers. Recent breeding population estimates for the Koyukuk NWR were 7-10,000 birds; thus, communities in the region harvested about 4% of the local population in 1998 and about 7.5% in 1999.

Total duck harvest in 1999 was about half that in 1998. The decrease was exhibited fairly equally among all the major species, suggesting lower participation in waterfowl hunting in 1999 (*see* p. 10). However, the decline in harvest numbers may also reflect the lower abundances of waterfowl in the Koyukuk stratum in 1999 as shown in Table 5. In 1999 counts, all species but scaup were equal or lower than in 1998. Wigeons, mallards, and shovelers showed the most remarkable declines.

Harvest comparison to prior surveys in the same region

Harvest estimates in the present study are considerably lower than prior estimates in the same region based on the Household Interview Technique (Marcotte 1983, Marcotte and Haynes 1985, Looman 1987, Marcotte 1990, USFWS Galena 1992, unpubl. data) (Table 6). The 1998 and 1999 duck estimates were only 17% and 33%, respectively, of the 1974 harvest estimates. Similarly, the 1998 and 1999 goose estimates were 31% and 34%, respectively, of the 1974 harvest estimates, and 63% and 69% of the 1992 harvest estimates. There was one exception to this decline: Households in Koyukuk reported more geese harvested in 1998/99 than in 1992. Despite declines in numbers, the relative contribution by each community to the total harvest has remained somewhat stable. For example, goose harvests have always been relatively high in Huslia. Harvests in Ruby have been comparably low, particularly in 1974. Our results substantiate the conclusion that waterfowl harvest does not play a predominant role in subsistence activities for Ruby residents (*see* p. 7). This may be due to lower waterfowl abundance in the area's high proportion of upland habitat.

Hunters' perceptions of waterfowl population trends in 1998

During the spring survey in 1998 we asked current and former hunters if they had noticed changes in the size of waterfowl populations over the years they have hunted. Many people have harvested waterfowl in their community for more than 20 years and some had been hunting up to 70 years. These hunters are a highly valuable source of general information on long-term population changes. For the most part,

people thought that waterfowl populations were declining. A majority of households (172, 59%) felt that there are fewer birds since they began hunting. Sixty-five households (23%) did not know if there was any change, 34 (12%) thought that there was no change and only 21 (7%) felt that there were more waterfowl. Hughes, Koyukuk, and Huslia had the largest share of households (> 65%) that thought there were fewer waterfowl. In Ruby and Nulato approximately half of households did not know if there was a change, or thought that there was no change. Number of years of hunting in the community had an influence on waterfowl trend estimates made by hunters (Fig. 4). Most households that reported an observed decline in waterfowl had hunters that had been active in their community for 40+ years (57 households, 80%). Various reasons for the decline were proposed, including weather conditions (cold spell in 1992/93, El Nino), predation by fox and pike, the Prince William Sound oil spill, possible changes in migration routes, feeding of geese in Anchorage, and government goose banding projects. A few people emphasized the high annual variability of waterfowl abundance. Some people stressed that populations were not over-hunted by subsistence hunters in the area because "we only take what we need."

Most households thought that geese and ducks were declining in general, but only a small share of households commented on particular species or groups of birds that they felt exhibited changes in population size (Fig. 5). Answers were somewhat contradictory, possibly reflecting local differences in waterfowl populations. However, comments on swans and snow geese were remarkably similar. Sixteen households mentioned that they had seen more swans. This observation is corroborated by the slight rise in pair numbers observed during aerial swan surveys conducted by the USFWS in Galena since 1985 (Lowe and Spindler 1996). Fourteen households mentioned that there used to be far more snow geese (commonly referred to as 'white geese'). These hunter assessments of population change, however, should be considered with caution, since unusual weather conditions in spring 1998 apparently caused low waterfowl availability, which might have influenced people's judgements.

Egg harvests 1998 and 1999

No egg harvest was reported in this survey. One person from Nulato mentioned that they used to eat goose eggs in the 1950's. According to Marcotte (1990) and Wolfe et al. (1990) egg harvesting in the area was primarily incidental. We might have missed some egg harvests during the first year of survey because households were not asked about egg harvests on a regular basis.

Comparison of Harvests in 1998 and 1999 by Communities

In 1998, Galena harvested the largest proportion of birds (22.8%), followed by Huslia (19.6%) and Koyukuk (15.2%, Table 7). Kaltag and Ruby each harvested less than 10% of the total. The ranking shifted for per capita harvests: Hughes was by far top-ranking (7.3 birds/capita), followed by Koyukuk (3.2 birds/capita) and Huslia (2.2 birds/capita) (Table 6). Ruby was lowest with an average of less than one harvested bird per person. In terms of usable pounds harvested per community, Huslia was first (22.6% of the total harvest), followed by Galena (19.6%) and Koyukuk (17.5%). Ruby again harvested the smallest share (4.2%). Ranking for pounds harvested per capita was similar to ranking for birds

harvested per community: Hughes, Koyukuk and Huslia showed the largest weight harvested per person and Ruby the least.

In 1999, waterfowl harvests in Galena, Huslia, Koyukuk, Kaltag, Hughes and Ruby ranged from 48% (Hughes) to 98% (Ruby) of the harvests reported in 1998. Nulato was the only community which harvested considerably more in 1999 than in 1998. Nulato harvested 26% of the 1999 total, followed by Galena (18%) and Huslia (17%) (Table 6). Hughes and Ruby each harvested less than 10% of the total in 1999. In terms of per capita harvest Hughes was top-ranking again (3.0 birds/capita) followed by Koyukuk (2.9 birds/capita) and Nulato (1.4 birds/capita). Ruby and Galena were last with 0.7 and 0.6 birds per person, respectively. Nulato harvested the largest number of usable pounds per community (26%), followed by Huslia (20%) and Galena (15%). Ruby again harvested the smallest portion (5%). Koyukuk and Hughes harvested the most useable weight per capita in 1999.

In 1998, Kaltag harvested the highest species diversity; 14 bird species were taken. In Ruby, only nine species were harvested. These differences were primarily due to single harvests of some species in Kaltag (snow goose, bufflehead, merganser). In 1999, species diversity within communities was not as high. Kaltag and Hughes were lowest with only six species each, and Koyukuk and Nulato were highest with 11 bird species harvested. However, the composition of the major harvest species was similar among villages in both survey years. In 1998, six species were taken in every community (Canada and white-fronted goose, American wigeon, American green-winged teal, mallard, and northern pintail). Four species were taken in every community in 1999 (Canada and white-fronted goose, mallard, and northern pintail). Surprisingly, no wigeons were harvested by Kaltag residents in 1999, while this species was highest among harvested ducks in Galena (112).

The harvest profile varied by community and year, most likely due to local differences in waterfowl abundance or availability. In 1998 Koyukuk and Huslia harvested more geese than ducks, a pattern which included Nulato and Kaltag in 1999. Furthermore, Huslia was the only community that took comparably high numbers of white-fronts both in 1998 and 1999: In 1998 Huslia harvested almost as many white-fronts as Canada geese and those harvests accounted for 38% of the total white-front harvest. The high harvest of white-fronts in the Huslia area is likely related to habitat differences in the area surrounding the village. In the early 1990's, white-fronts consistently outnumbered Canada geese in the Huslia harvest estimates made by a resident contracted by USFWS (USFWS Galena, unpubl.).

In 1998, duck species most commonly taken in Nulato, Kaltag, and Ruby were mallards, while in Galena, Huslia, and Koyukuk, wigeons were harvested most frequently. In 1999, mallards were also top-ranking in Hughes and in Koyukuk, whereas households in Huslia took mostly northern pintails. In 1998, Oldsquaw were harvested primarily by the Koyukuk River communities, Huslia and Hughes. Scoters (commonly referred to as 'black ducks') were also mainly taken by households in Huslia and Hughes.

In the spring of 1998, Hughes reported a harvest of 80 buffleheads, making that species top ranking among ducks harvested there. It is possible that this was an inaccurate record. Reportedly, people from the whole community had brought their harvest to this single household to store in a freezer before a potlatch. The head of the household was not able to remember his own share. No other interviewed

households confirmed bufflehead harvest, which might imply that birds of another species were mistaken for buffleheads.

Comparison between Spring Harvest and Summer-Fall Harvest 1998 and 1999

Spring harvest in both years was considerably higher than summer and fall harvest. In 1998 and 1999, 64% and 76%, respectively, of the annual waterfowl harvest was taken in the spring. High spring waterfowl harvest was primarily due to high goose harvests in the spring; in 1998 about four times more geese were harvested in the spring than in the summer and fall, and in 1999 the differences were even more pronounced (Fig. 6, also see Table 2 and Table 3). Duck harvests also tended to be lower in the summer and fall. In 1999, duck harvests in the summer and fall were almost 50% lower than in the spring. More geese were taken than ducks in both springs: In 1998, spring goose harvest amounted to 50.3% of the total spring harvest, while in the spring of 1999 almost twice as many geese were taken as were ducks (Fig. 6). In the summer and fall, duck harvests were higher than goose harvests in 1998 (79% of the summer and fall harvest) and 1999 (61% of the summer and fall harvest).

Differences in numbers of geese harvested in spring and summer-fall were even more distinct within white-fronted geese: White-front harvests were more than 10 times higher in the spring of 1998 than in summer/fall, and eight times higher in the spring of 1999. Several people mentioned that it was too difficult to harvest geese in the fall because they are 'too wild', an observation also recorded by Marcotte and Haynes (1985). Also, a telemetry study on Koyukuk NWR (Spindler et al. 1998) suggested that once white-fronted geese regain flight after the molt, and before fall hunting season, most migrate directly from the breeding grounds to pre-migratory staging areas outside the harvest study area.

Within ducks, mallards and wigeon were most frequently harvested in all periods of the study In 1998, more mallards, green-winged teal and scoters were harvested in the summer and fall than in the spring, while fewer wigeon, pintail, shoveler, oldsquaw and scaup were harvested in summer and fall. These dynamics were similar in 1999, with one exception: mallards were harvested more frequently in the spring (71% of the annual mallard harvest).

In all communities except Nulato, the 1998 summer and fall harvest was at least 20% lower than the spring harvest (Fig. 7). The most drastic decrease was in Hughes, where only 28 birds were harvested during the summer and fall compared to 352 birds in the spring. In 1999, harvests in every community were more than 50% lower in the summer and fall than in the spring (Fig. 8). In 1998, some communities (Galena, Huslia, Nulato, Kaltag) harvested more ducks during summer and fall than in the spring. In 1999, duck harvests were lower in every community in the summer and fall compaired to spring. In terms of usable weight, most of the annual harvest was taken in the spring in 1998, a pattern even more pronounced in 1999. The reported consumption in each community was higher in the spring, and differences in seasonal harvest importance were greater when weight was considered.

Participation in waterfowl hunting and use in 1998 and 1999

On average, more than two thirds of interviewed households did not hunt waterfowl in 1998. A small number of households hunted but did not harvest anything. In 1998, about 25% of households harvested waterfowl (Fig. 9). In 1999, fewer households (19%) hunted and harvested waterfowl. Slightly more people hunted but did not harvest anything (about 6%), and an average of 76% did not hunt in 1999 (Fig. 10). In both survey years, Galena, Nulato and Huslia had the highest number of harvesting households. Percent household participation within the community, however, was consistently highest in Koyukuk, Hughes, and Huslia, and lowest in Ruby and Galena.

Spring hunts not only proved to be most important in terms of harvest numbers, but also in terms of participation in waterfowl hunting; most people hunted and harvested waterfowl in the spring. In 1998 about 36% of households participated in waterfowl hunting in the spring and about 23% did so in the summer and fall. In 1999 about 32% hunted in the spring and about 17% in the summer and fall. In 1999, participation in hunting dropped in each community in the summer and fall. In 1998, Koyukuk was the only exception, showing one more household going out in the summer and fall than in spring.

Since the fall survey in 1998 we have asked people about their most important season for waterfowl hunting. Preferences within communities varied considerably among the three included survey rounds, but there was a definite preference for spring hunting: More than 80% of households that hunt waterfowl reported that they go out in the spring; for at least 50% it was the *only* time of the year they hunted (Fig. 11). Between 36% and 49% reported going out in the fall, but only 9% to 14% reported that they *only* hunt in the fall. In the summer most people in this region do not hunt waterfowl; in 1998 only one household reported hunting in the summer, and one reported going in the summer and in the fall. Slightly more people reported summer hunting in 1999. Some people emphasized that they do not hunt while the birds are breeding and raising their young. During the fall 1999 survey we asked people to specify their preferred hunting months. May was top-ranking (187 households), followed by April (135 households) and September (80 households). August was named by 28 households, June by eight, and October by five (estimates for Galena were doubled from 50% random sample). It has been well known that spring is the most important season for waterfowl hunting in several regions of Alaska, including the Koyukon (Loranger 1985, Looman 1987, Marcotte 1990, Wolfe et al. 1990, Wentworth and Seim, 1996), and the pattern has not changed during the present study.

During each of the four survey rounds, 27-40% of households reported that they never hunt waterfowl. Those reports were relatively consistent: Twenty-seven percent of interviewed households reported not hunting in the spring of 1998, 37% in the fall of 1998 and in the spring of 1999, and 40% in the fall of 1999. Estimates from the very first survey round may be negatively biased, since some questionnaires from this survey were incomplete. Estimates of non-hunting from the last survey reflect the low harvest season and may be high relative to normal hunting participation.

The number of individuals participating in the waterfowl harvest was slightly lower in 1999 than in 1998. The spring harvest involved 206 people in 1998 and 218 in 1999. In the fall of 1998, 156 people hunted, while only 114 hunted in fall 1999. About 20% of households had more than one person who hunted, and this ranged up to six people per household.

More households reported using waterfowl than the number that harvested birds because sharing with other households or at potlatches was common (Table 8). In 1998, 22% of interviewed households shared waterfowl in the spring and 20% in the fall. In 1999, 29% of households shared waterfowl in the spring, and 14% in the fall. Of households which had harvested waterfowl in 1999, at least 60% shared. Of households which had harvested more than 20 birds in 1999, an average of 94% shared. A similarly large proportion of households received waterfowl from other households, 24% in the spring and 15% in the fall of 1998, and, despite of the lower harvest numbers, 41% in the spring and 22% in the fall of 1999. Households using waterfowl comprised between 33% (fall 1999) and 61% (spring 1999) of interviewed households. An average of 4% of interviewed households both shared and received in 1998, increasing to 10% in 1999. (The number of households both sharing and receiving waterfowl might be underestimated in 1998 because some questionnaires were not filled out completely). Extensive sharing of harvests among Koyukon Athabascan people has been widely documented (Nelson, 1983, Marcotte and Haynes 1985, Marcotte 1990).

Within communities, the portion of households that used waterfowl was largest in Huslia in 1998 (83% in the spring, and 51% in the fall) and largest in Koyukuk in 1999 (88% in the spring, and 68% in the fall). In all of 1998 and the spring of 1999, Ruby displayed the lowest proportion of households using waterfowl (ranging from 17% to 33%), and in fall 1999 Galena ranked lowest (25%).

Household participation and harvest numbers in both years were affected by the timing and occurrence of potlatches (community feasts), events of traditional and cultural importance which include sharing of food. In the spring of 1998, some primary hunters in Koyukuk and Hughes mentioned that most of their waterfowl harvest was destined for an upcoming potlatch. In the fall of 1998, two households in Ruby had solely hunted for a potlatch. In the spring of 1999, several potlatches took place in Koyukuk and Nulato. During the spring survey of 1998, at the request of one of the tribal councils, we asked people if any household members were Alaska Natives and whether they considered hunting to be a cultural activity. Eighty-eight percent of interviewed households were Alaska Native. The largest proportion of non-native households was in Galena (26%). Forty-two percent of Native households and 10 percent of non-native households harvested or attempted to harvest. Ninety percent of native households considered hunting cultural while 3% did not. Thirty-one percent of non-native households considered hunting cultural while 61% did not (missing percent are due to incomplete records, *see* above).

Efforts in hunting waterfowl in 1998 and 1999

In both survey years we asked households that had harvested or attempted to harvest waterfowl how many miles away from their community they had been hunting. Our goal was to determine the regional extent of community harvest effects on waterfowl populations. Because we did not want to ask people to reveal their favorite hunting places, we tried to get estimates of straight-line distances from the community. However, most of the information we received was in terms of river miles which made detailed comparisons difficult. The findings shown in Table 9 suggest that some hunters stayed close to their community (10 mi or less), while others traveled a much greater distance (more than 20 mi).

Waterfowl hunting often accompanied other activities such as moose hunting in the fall. The opportunistic nature of waterfowl hunting made it difficult to assess the amount of time individuals

spent hunting waterfowl, especially in the fall. Some households just took a few minutes to shoot a bird while actually doing something else. Others went out numerous times over several weeks but only for short amounts of time. Rough estimates of the number of days spent hunting waterfowl by regional households are presented in Table 9.

Were 1998 and 1999 'good' or 'bad' waterfowl hunting years?

To get an estimate of how well the reported harvests represented the average waterfowl take in the area, we asked hunters if they had taken more, less, or about the same amount as usual. In 1998, most households reported that they had harvested less than in previous years, especially in the spring (56%) (Fig. 12). Almost half of these households (35 households) attributed this to unfavorable weather conditions. Waterfowl were difficult to hunt in spring 1998, mainly because early thawing caused dangerous travel conditions and offered the birds more places to stay and rest than usual. In the summer and fall, households had harvested less primarily because they had other obligations. In 1999, a similarly large number of households reported harvesting less than in previous years (Fig. 13). Although travel conditions were better in the spring of 1999 than in 1998, people mentioned that the spring had been late and unusually cold. Reasons given for lower harvest in summer and fall 1999 were similar to those in 1998; about 20 households said that they did not go out because they were working. Twenty-eight households mentioned that ducks and geese had been sparse in the summer and fall of 1999 and were not seen flying over villages as usual. A few households in Nulato reported that they were not able to hunt in the Kaiyuh flats in the summer and fall of 1999 because low water levels prevented boat access. Although harvest numbers were substantially lower in 1998 than in 1999, slightly fewer households reported having harvested less (Fig. 13) This could be due to the fact that bird harvests per *harvesting* household did not differ much between years. Within harvesting households, an average of 12.1 birds and 10 birds per household was harvested in the spring and fall of 1998, respectively. In 1999 an average of 12.3 birds and 8.8 birds per household was harvested in the spring and fall, respectively. However, there was a high variation in average birds taken per hunting household among seasons and years within communities.

Problems and Recommendations

In 1998, people from a few households in Koyukuk and Hughes hunted together and were not able to remember their own share. In most cases observers could assign each household a portion of the total number of reported birds harvested. This was not possible in two cases because hunters of the concerned households had hunted more than once in different combinations. I believe that any resulting multiple counts would probably compensate for harvests of hunters that we may have missed. Nevertheless, any future surveys must consider that shared waterfowl harvest among households will require extra effort to document. Without careful effort it will be difficult to adequately take the Athabascans' valued practice of harvest sharing into consideration when regulations are implemented to realize the Migratory Bird Treaty Act amendments.

During the first survey in the spring of 1998 we handed out black-and-white bird identification sheets and a calendar for noting birds harvested by month. The intent was to help people to keep track of their

harvest during the relatively long summer and fall period (May 20th - October 8th). We planned to pick up the calendars during the fall survey. This method has been used successfully by ADF&G for village subsistence salmon harvest monitoring. Although a few people mentioned that they had used the bird pictures to help with the identification, we did not get any calendars back, and thus did not receive any written information from hunters during these months. Instead, we had to rely on hunters' memory of the harvests. Another harvest calendar was mailed out to box holders in early spring 1999. Because the response to this calendar was similarly poor (we received four calendars) we refrained from another distribution. This approach may take a long time to become established before it can provide useful information.

Except for Huslia and Galena, where the main surveyors were residents, interviews were conducted by a USFWS employee and a local assistant. Travel logistics limited the amount of time available for the USFWS employee to survey these communities, and households that were absent could not be contacted. In the fall survey in 1998 and in 1999 we were able to increase coverage in some communities by having local assistants interview some households at a later date. The teamwork between the contracted local resident in each village and the visiting USFWS employee worked well. It should be emphasized that the assistance of a local resident in each community is crucial to a successful survey. A resident knows, for example, which houses are currently occupied and empty, and knows where to catch people who are not home. On the other hand, using the same USFWS surveyors maintains consistency in data recording.

Conclusions

Subsistence harvest of migratory birds in the villages of Galena, Ruby, Koyukuk, Kaltag, Nulato, Huslia, and Hughes was estimated through household interviews in 1998 and 1999. Total harvest was estimated at 2,733 and 2,064 birds in 1998 and 1999, respectively. Total edible weight of this harvest was estimated at 6,717 lbs and 6,103 lbs in the same years, respectively. A majority of this harvest occurred in the spring. A majority of the harvested weight was Canada and white-fronted geese. Frequently harvested duck species included mallard, American wigeon, and northern pintail. In 1999 the harvest of white-fronted geese increased markedly. Changes in species harvest could have been caused by differing availability, hunter access, and timing in abundance of birds. Sharing of the waterfowl harvest among households within a village was found to be a frequent practice. The greatest total harvest occurred in the villages of Galena, Nulato, Huslia, and Koyukuk. Hughes ranked highest in per-capita harvest, while Ruby ranked the lowest. Most waterfowl hunters traveled 0-10 miles or >20 miles from their village to hunt (similar numbers of each), while the smallest proportion traveled 10-20 miles to hunt.

Subsistence waterfowl harvest in the Koyukon region was lower in 1998-99 compared to estimates based on similar surveys conducted by the refuge in 1992, and by other researchers in the 1980's. This decline in harvest was probably related to changing socio-economic conditions in the region. Elder interviewees said that reliance on waterfowl has declined as the availability of jobs, freezers, and moose has increased in the last 20 years. The distribution of hunting effort has probably changed as people have become more tied to their village in order to work at their jobs and have their children attend

school. Also, there are more jobs available in the region's villages, and few families live out in spring camps as compared to the previous two decades.

The rate of harvest of white-fronted geese increased from an estimated 4% of the regional population in 1998 to 7.5% in 1999. Previous studies indicated that the total subsistence harvest of this species was higher in the 1980's and early 1990's; however, the present harvest may be significant given that the population of white-fronts has declined during the same period. Because geese made up the majority of edible weight and were most sought-after, the refuge should monitor harvest of white-fronted and Canada geese annually in the three villages where most of this harvest occurs (Huslia, Hughes, Nulato). This should be done using methods similar to this study through contracts with local observers. The entire study should be repeated in about five years, or when further resource concerns arise.

Acknowledgements

I would like to thank the Louden (Galena), Huslia, Nulato, Koyukuk, Kaltag, Hughes, and Ruby Tribal Councils for excellent cooperation and support of this project. A big thank you to all the people who participated in the survey and were willing to share information on their waterfowl harvest and hunting practices. Special thanks are due to the persons who assisted with the survey: Ted Jones, Harry O. Jones (Koyukuk), George Semaken, Pat Madros, Sr., Pat Madros, Jr. (Nulato), Richard Miller, Joseph Dentler (Kaltag), Norma Williams, Carlson Koyukuk (Ruby), Wilbert Koyukuk (Hughes) and James Honea (Galena). Refuge Information Technician (RIT) Robert Farmer (Huslia) assisted in preparing and conducting the survey. Orville Huntington (USFWS, Galena) provided input on the survey form and made initial and follow up contacts with Tribal Councils. Mike Spindler (USFWS, Galena) supervised the project and edited reports. Karin Lehmkuhl edited and assisted with completion of the final report. Joanna Roberts assisted with some village surveys and reviewed the report. Cynthia Wentworth (USFWS, Anchorage) commented on the survey form and provided many helpful suggestions. Susan Georgette (ADF&G, Kotzebue and Nome) provided materials and information about previously conducted waterfowl harvest surveys. Dave Anderson (ADF&G, Fairbanks) provided a Galena household list. This study was made possible with funds provided by the USFWS Regional Office, Division of Migratory Birds, in Anchorage, and by the support of Refuge Manager Eugene Williams.

Literature cited

ADF&G Div. of Subsistence and Maniilaq (1994): Bird harvests in Shungnak, household survey, 8pp.

Bellrose, Frank C. (1980): Ducks, geese and swans of North America, Third edition, Stackpole books, Washington, 540pp.

Hodges, J. I., J. G. Kin, B. Conant, and H. A. Hanson (1996): Aerial surveys of waterbirds in Alaska 1957-94: population trends and observer variability. National Biological Service Information and Technology Report 4. 24pp.

Kawerak, Inc. and ADF&G Div. of Subsistence (1997): St. Lawrence Island's bounty of birds, household survey, 2pp.

Looman, S. (1987): Use of wild resources by Ruby residents: A report on a 1984 household survey. USFWS Galena, Alaska. 36 pp.

Loranger, A. (1985): Historical and contemporary subsistence harvest of migratory birds in Alaska. USFWS, Wildlife Assistance, Anchorage, draft 114pp.

Lowe, J.M. and M.A. Spindler. 1996. Summary of aerial swan production surveys on the Koyukuk and Nowitna National Wildlife Refuges, Alaska, 1985-95. Prog. Rep. FY96-02, USFWS Galena, Alaska. 29pp.

Maniilaq Association and ADF&G Div. of Subsistence (1997): Bird hunting in Buckland, household survey, 15pp.

Marcotte, J. R. (1983): Contemporary resource use patterns in Huslia, Alaska, 1983. ADF&G, Div. of Subsistence, Fairbanks, Technical paper No. 133, 51pp.

Marcotte, J. R. and T. L. Haynes (1985): Contemporary resource use patterns in the upper Koyukuk region, Alaska. ADF&G Div. of Subsistence, Fairbanks, Technical paper No. 93, 122pp.

Marcotte, J. R. (1990): Subsistence harvest of fish and wildlife by residents of Galena, Alaska, 1985-1986. ADF&G, Div. of Subsistence, Juneau, Technical paper No. 155, 176pp.

Nelson, R. K. (1983): Make prayers to the raven: A Koyukon view of the Northern Forest. The University of Chicago Press, Chicago. 291pp.

Paige, A.W., CH.L.Scott, D.B. Andersen, S. Georgette, and R.J. Wolfe (1996): Subsistence use of birds in the Bering Strait Region, Alaska. ADF&G, Div. of Subsistence, Juneau, Technical paper No. 239, 127pp

Saperstein, L. (1997): A summary of duck production surveys on the Koyukuk and Northern Innoko National Wildlife Refuges, Alaska, 1983-1993. Prog. Rep. FY97-02, USFWS Galena, Alaska.

Schroeder, R. F., D. B. Andersen, R. Bosworth, J. M. Morris, and J. M. Wright (1987): Subsistence in Alaska: Arctic, Interior, Southcentral, Southwest, and Western regional summaries. ADF&G Div. of Subsistence, Juneau, Technical paper No. 150, 690pp.

Spindler, M A., J. M. Lowe, J. Y. Fujikawa, and R. A Stehn (1998): Regional trends of white-fronted geese based on aerial and float surveys in western interior Alaska. Poster paper presented to 9th North American Arctic Goose Conference, January 7-12, 1998, Victoria, B.C., Canada. 9pp.

Spindler, M.A., J. M. Lowe, and J. Y. Fujikawa (1999): Trends in abundance and productivity of white-fronted geese in the taiga of northwest and interior Alaska. Final report FY99-03, USFWS, Galena, Alaska. 29pp.

Spindler, M.A. (1999): Trends in abundance and productivity of whitefronted geese in the taiga of northwest and interior Alaska - 1999 update. Unpublished report, USFWS, Galena, Alaska, 7pp.

Wentworth, C. and S.G. Seim (1996): Subsistence waterfowl harvest survey Yukon-Kuskokwim Delta. Comprehensive Report 1985-1995 and Results 1995. USWFW Migratory Bird Management, Anchorage, 236pp.

Wolfe, R. J., A. W. Paige, and C. L. Scott (1990): The subsistence harvest of migratory birds in Alaska. ADF&G Division of Subsistence, Juneau. Technical paper No. 197, 18pp.

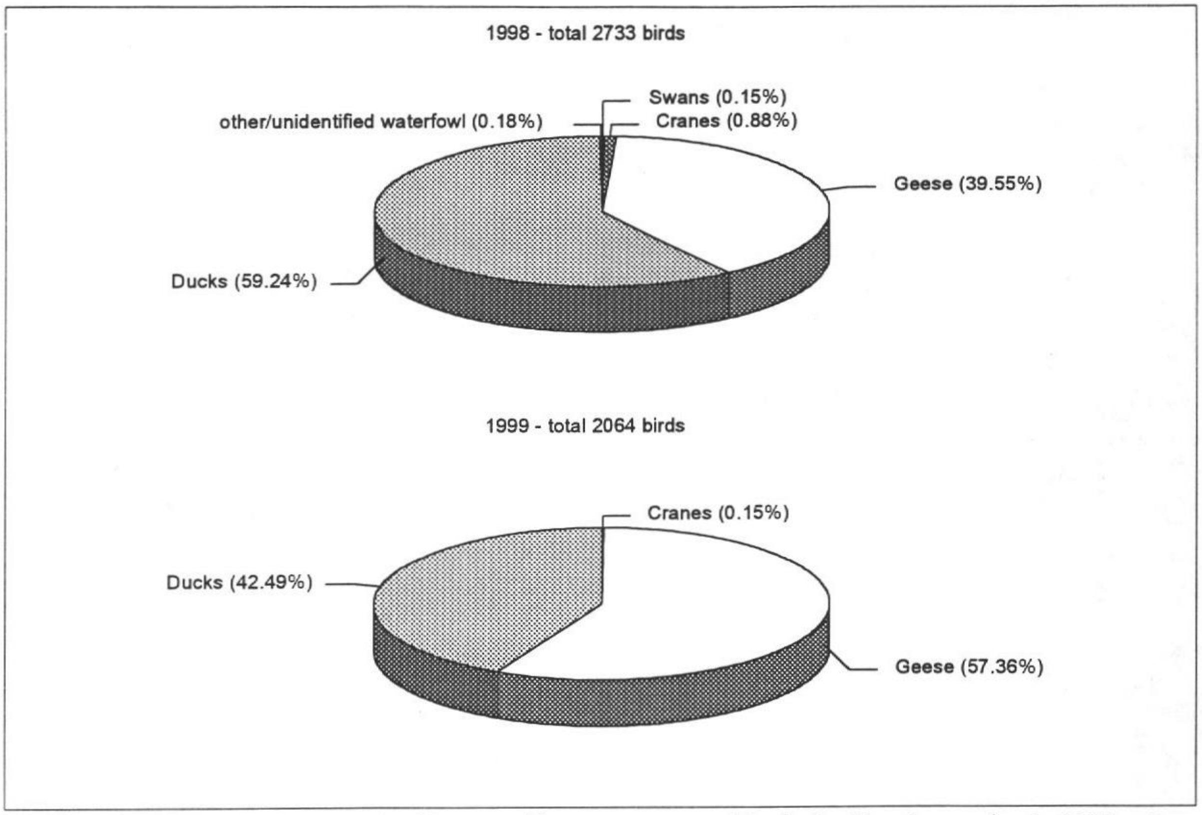

Fig. 1. Estimated number of waterfowl harvested by seven communities in the Koyukon region in 1998 and 1999 based on household interviews.

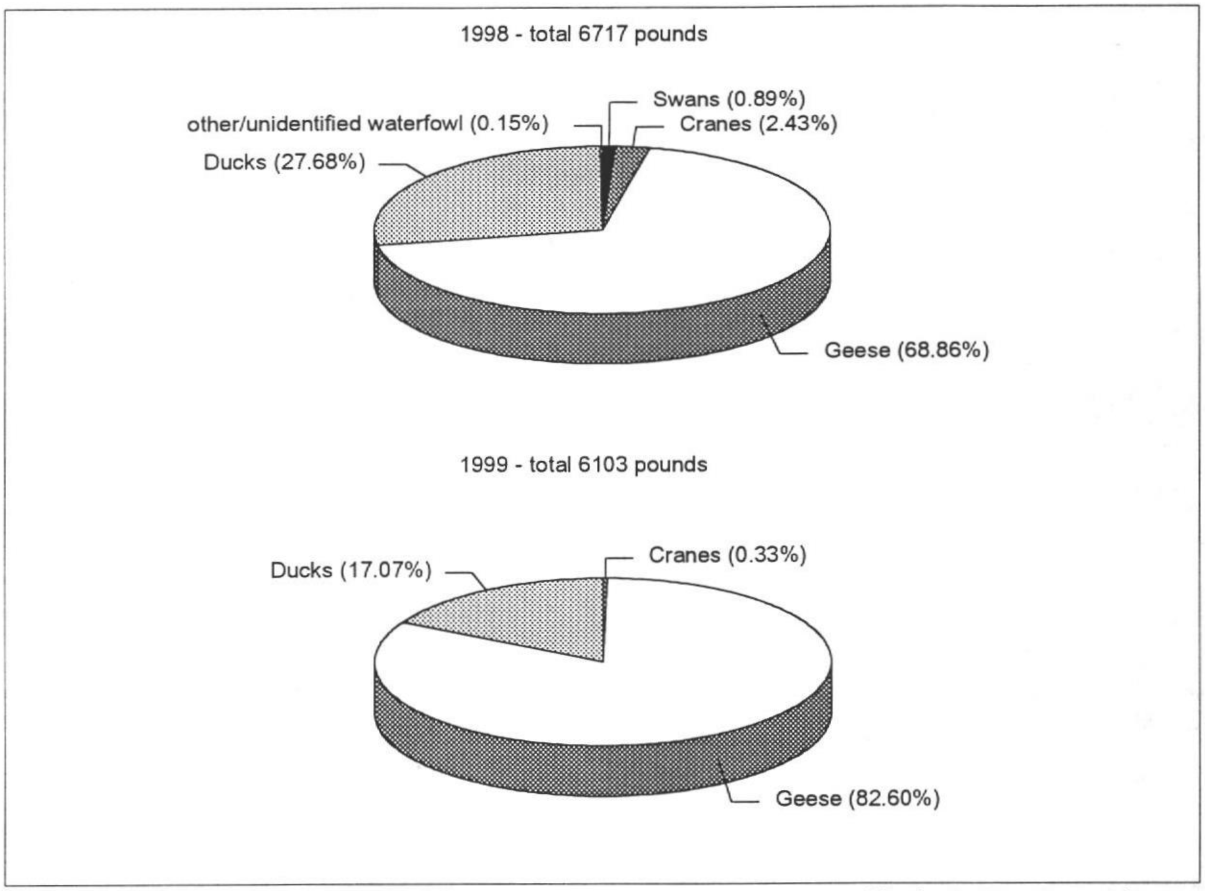

Fig. 2. Estimated pounds of edible waterfowl meat harvested by seven communities in the Koyukon region in 1998 and in 1999 based on household interviews.

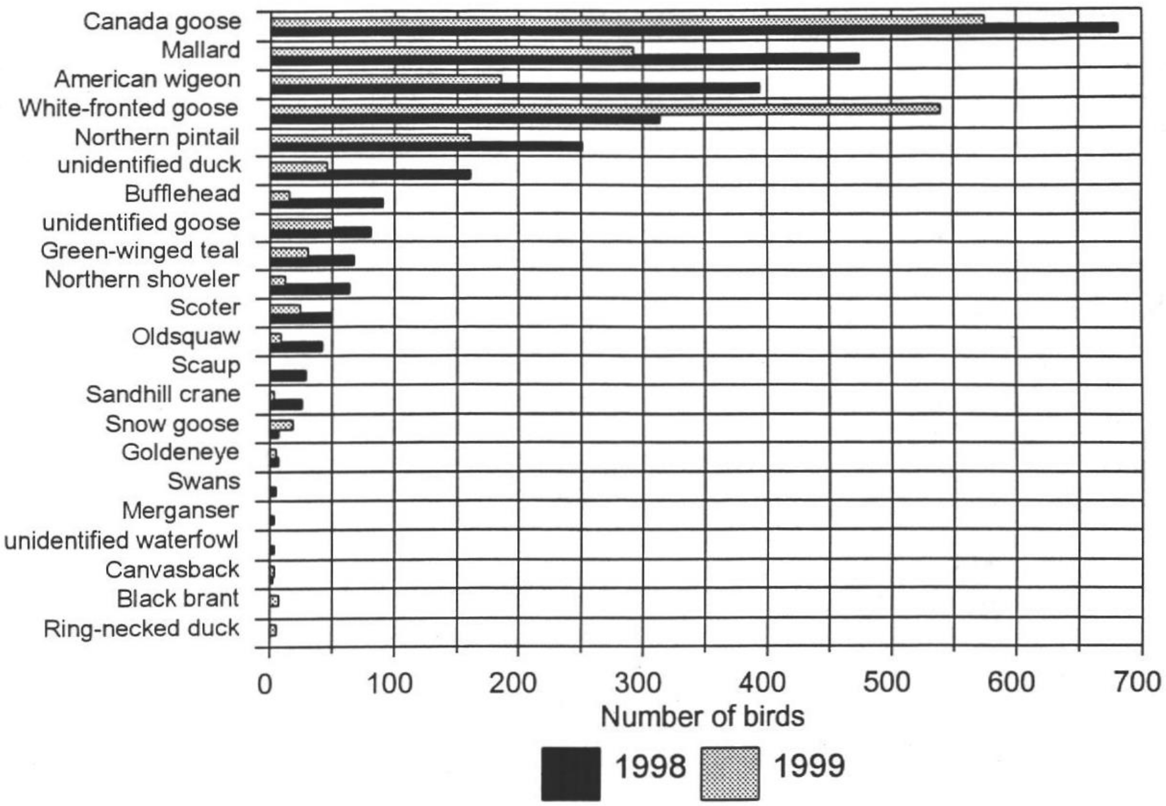

Fig. 3. Estimated number of waterfowl harvested (by species) in seven communities of the Koyukon region in 1998 and in 1999, based on household interviews.

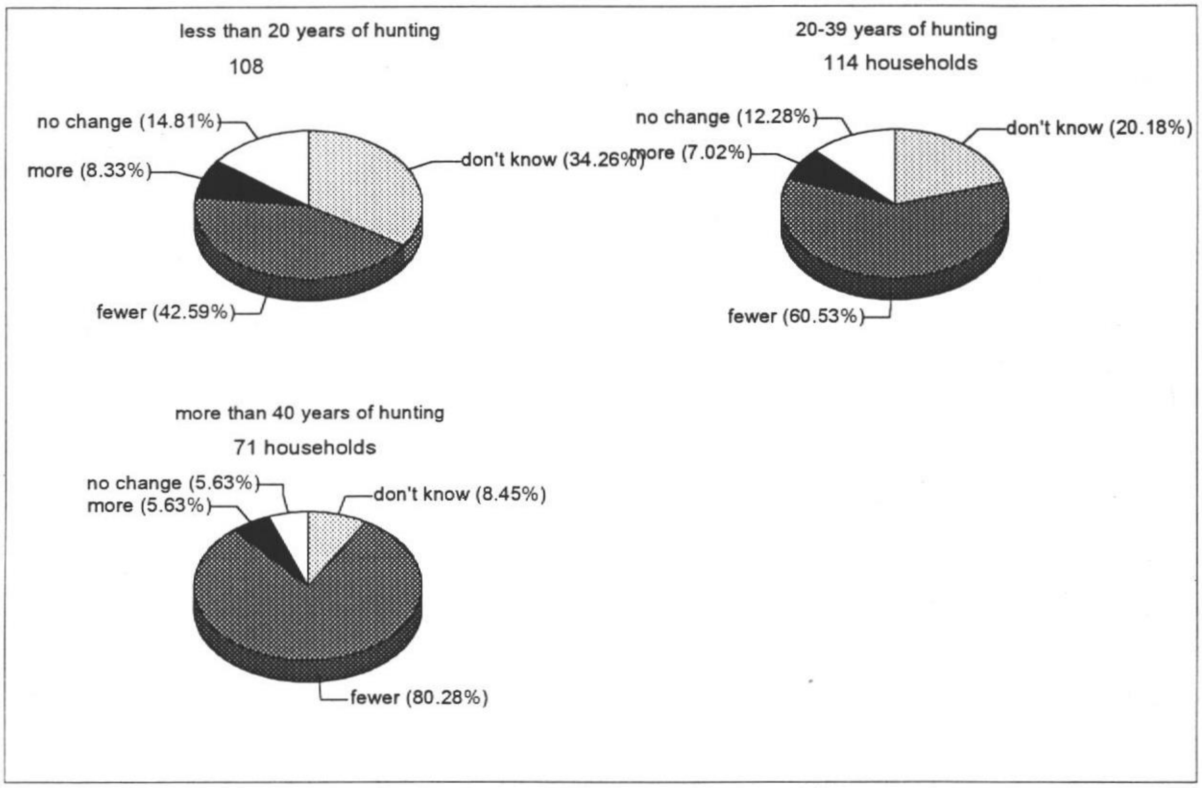

Fig. 4. Years spent waterfowl hunting in the community in relation to perceived trend in waterfowl numbers, based on interviews in 293 households of seven communities in the Koyukon region, spring 1998.

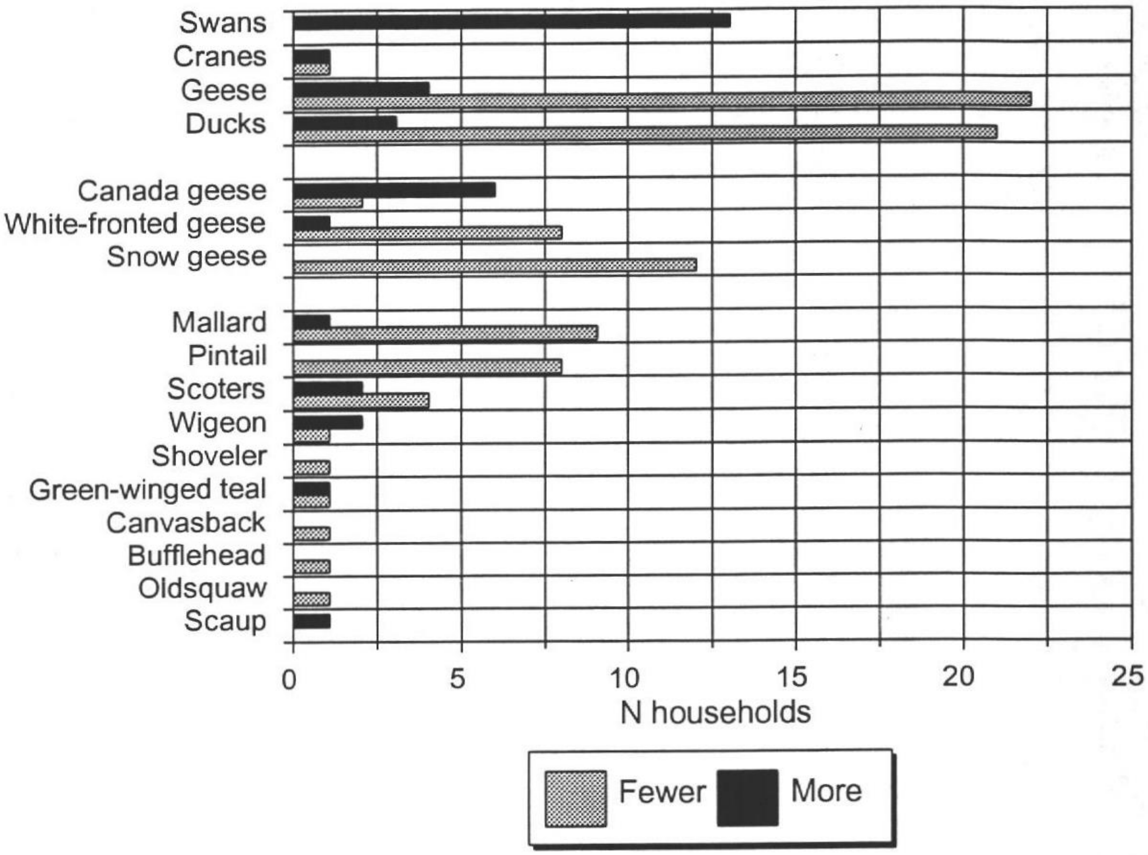

Fig. 5. Hunter comments on changes in population size of different types of waterfowl obtained during household interviews of seven communities in the Koyukon region, spring 1998.

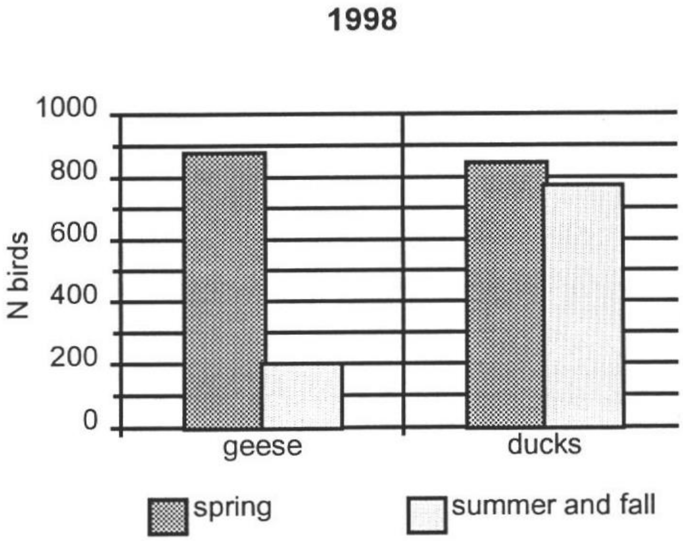

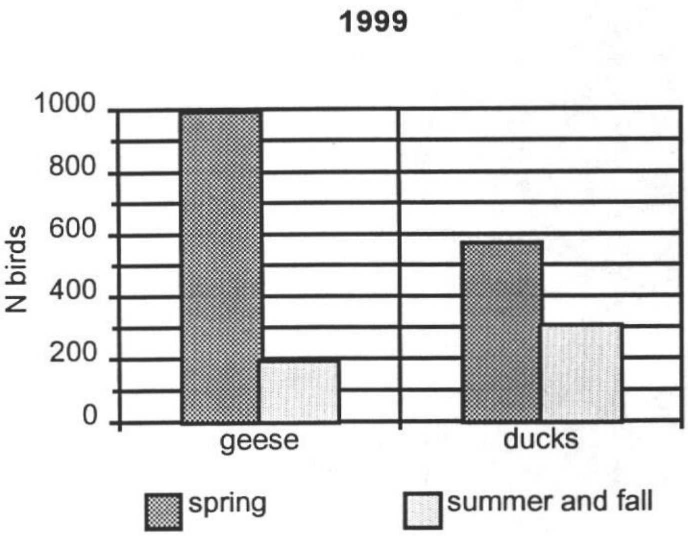

Fig. 6. Seasonality of goose and duck harvests in seven communities in the Koyukon region in 1998 and in 1999.

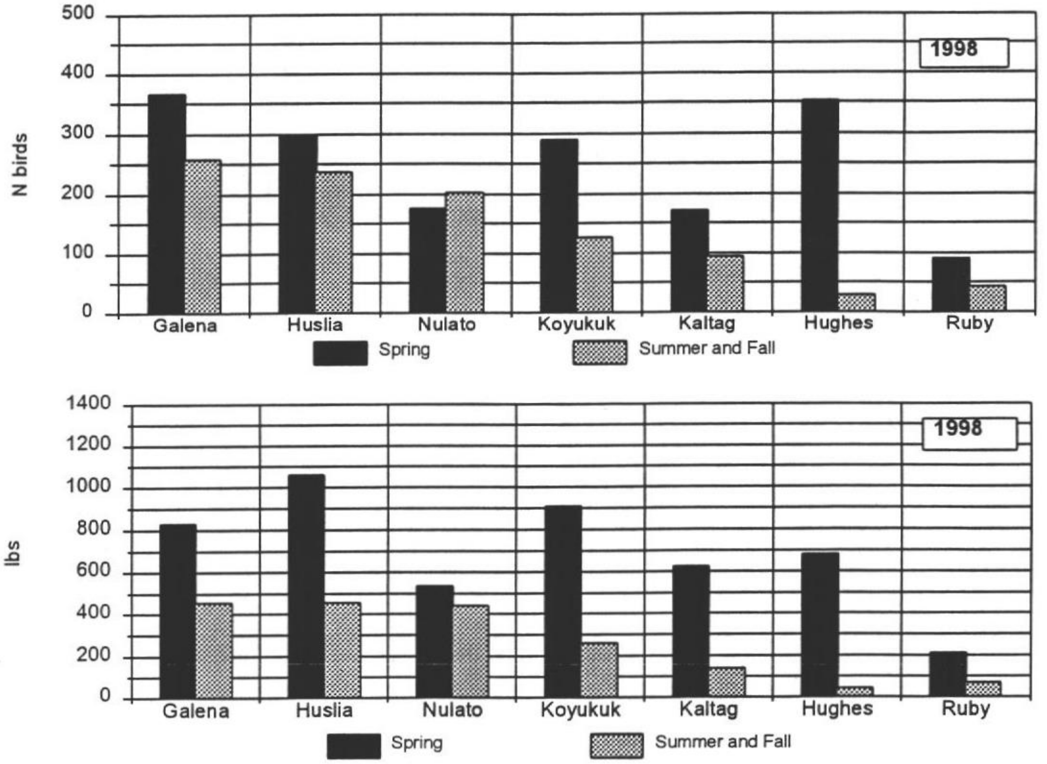

Fig. 7. Waterfowl harvest in numbers of birds (top) and in pounds of edible meat (bottom) in seven communities in the Koyukon region in 1998, based on household interviews. Estimates for Galena are expanded from a 50% random sample.

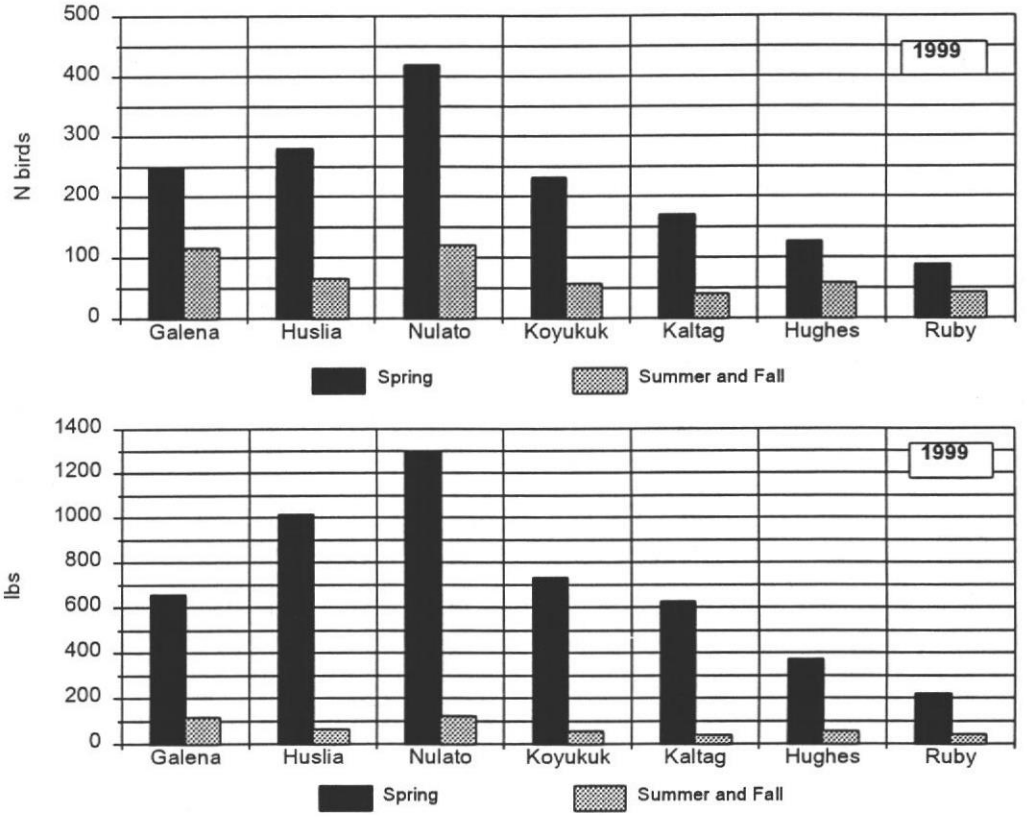

Fig. 8. Waterfowl harvest in numbers of birds (top) and in pounds of edible meat (bottom) in seven communities in the Koyukon region in 1999, based on household interviews. Estimates for Galena are expanded from a 50% random sample.

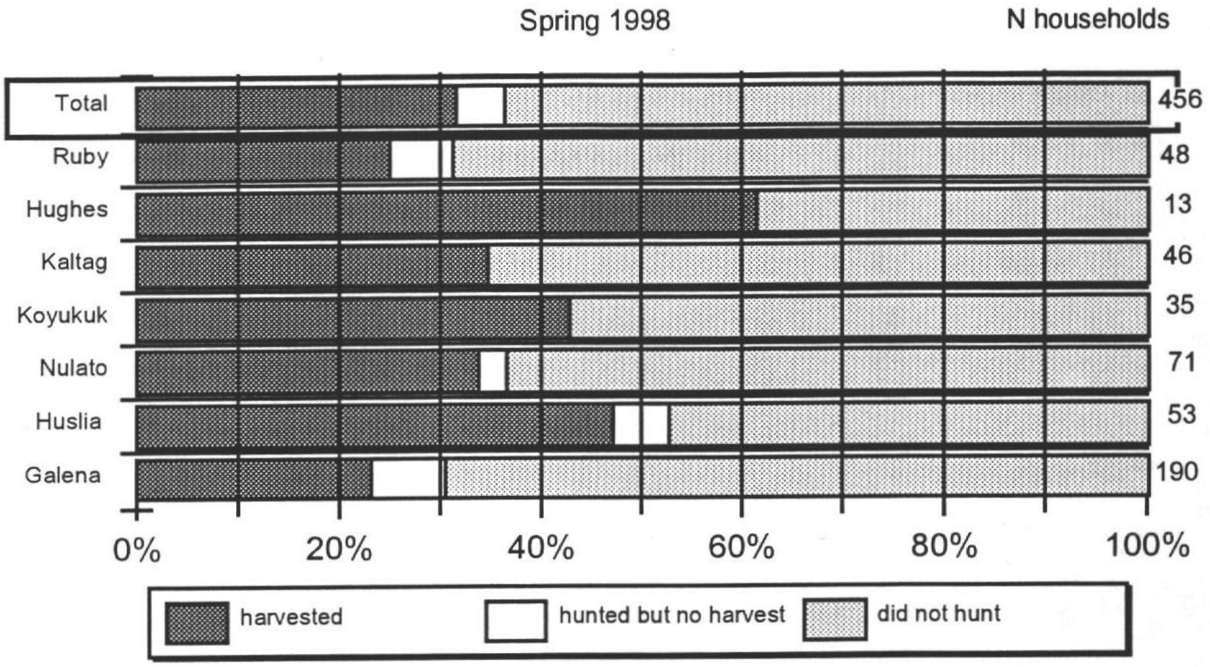

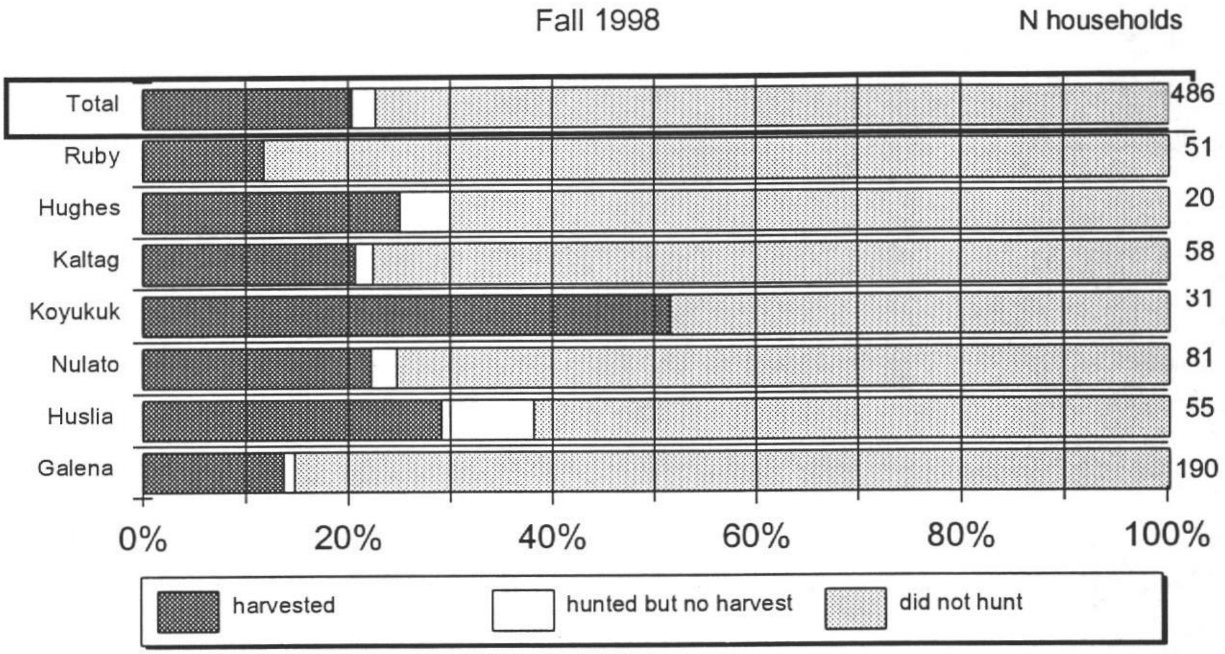

Fig. 9. Household participation in waterfowl hunting based on surveys in seven villages in the Koyukon region, 1998. Estimates for Galena were expanded from a 50% random sample.

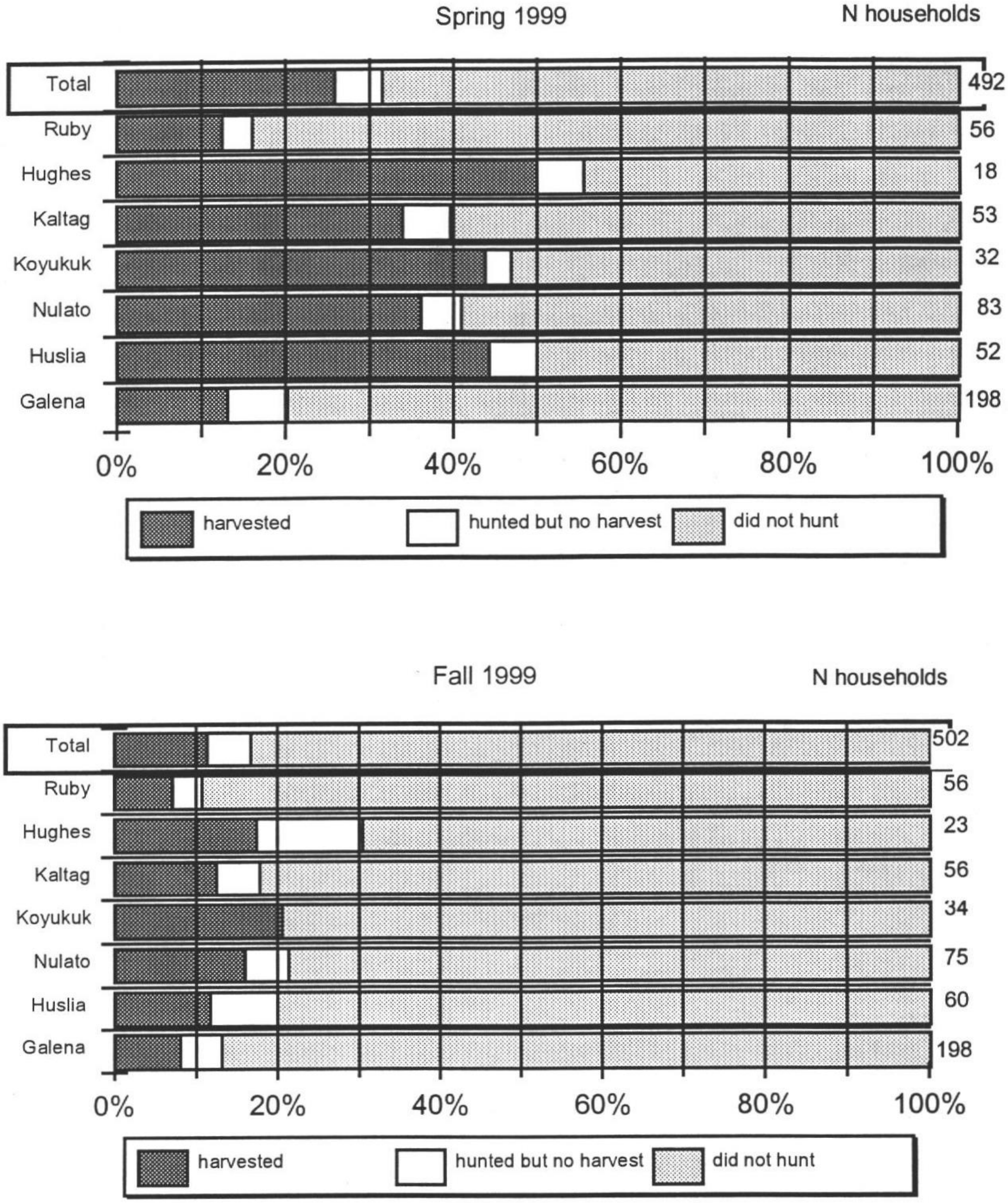

Fig. 10. Household participation in waterfowl hunting by seven villages in the Koyukon region, 1999. Estimates for Galena are expanded from a 50% random sample.

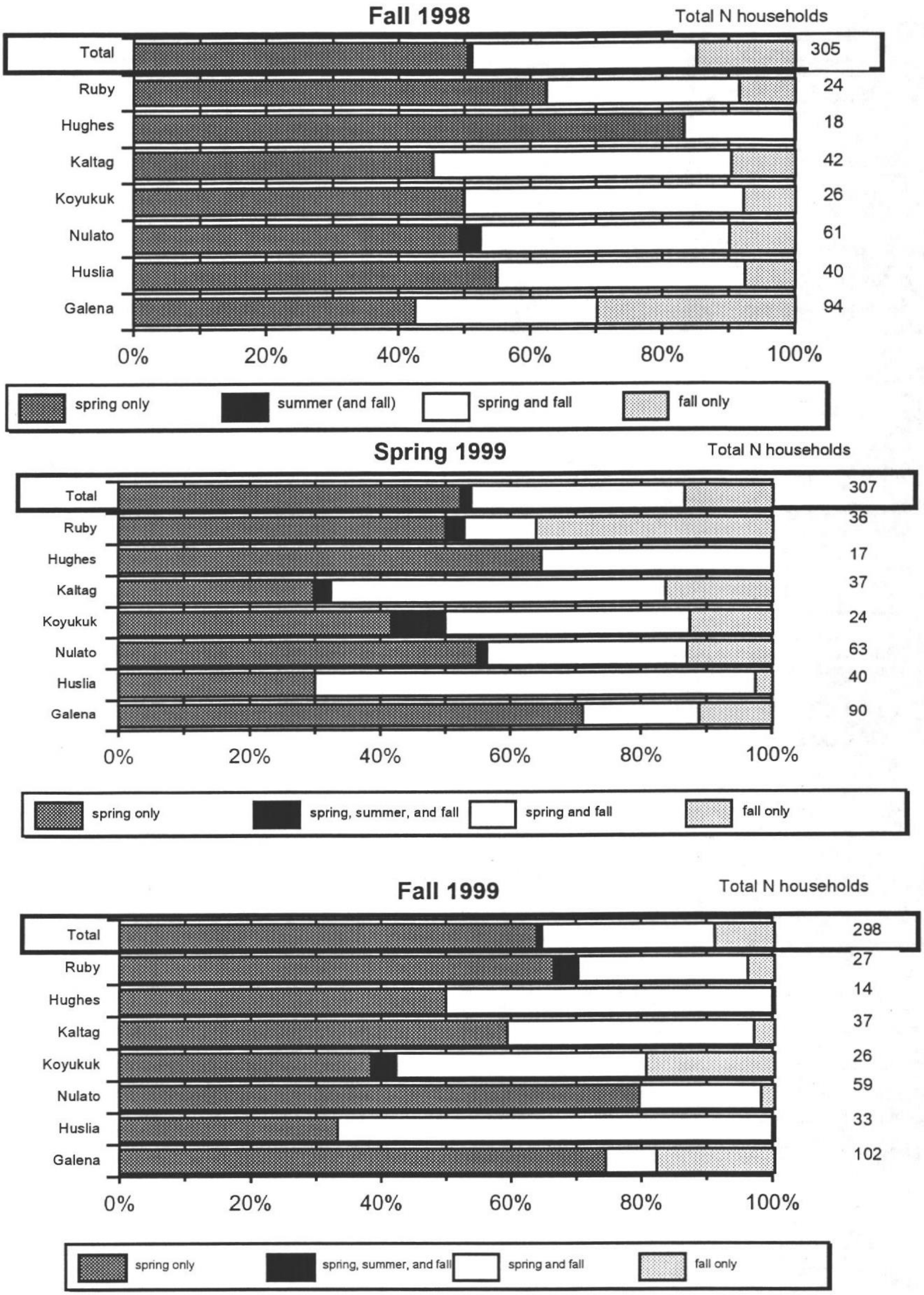

Fig. 11. Seasonality of waterfowl hunting reported by households in seven communities in the Koyukon region during 1998-1999 household surveys. Estimates for Galena are expanded from a 50% random sample.

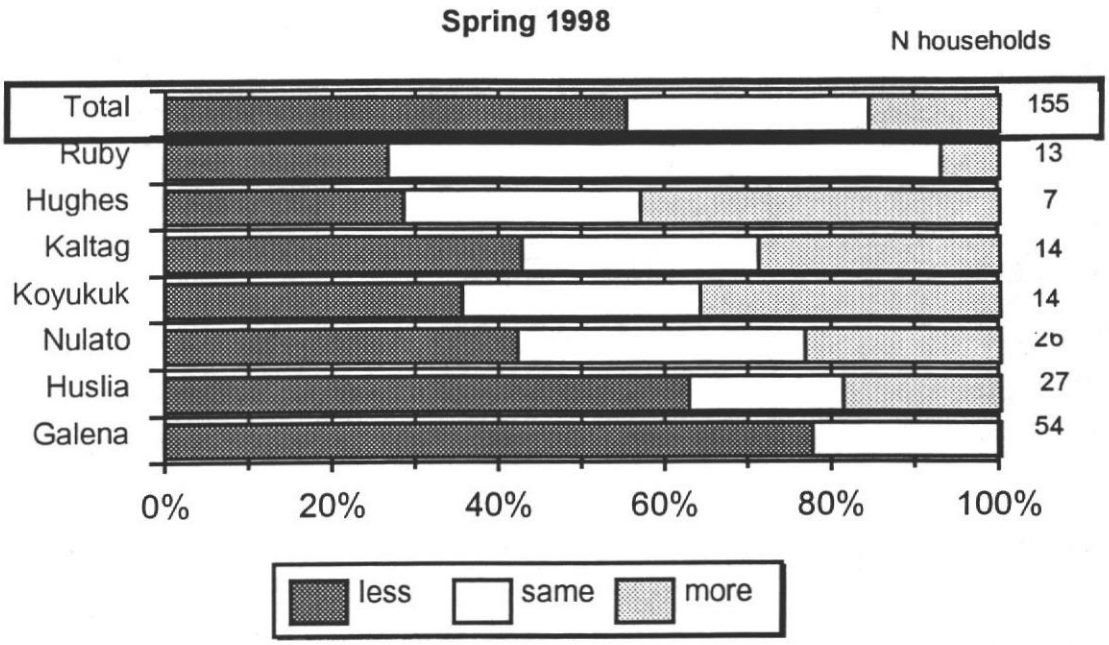

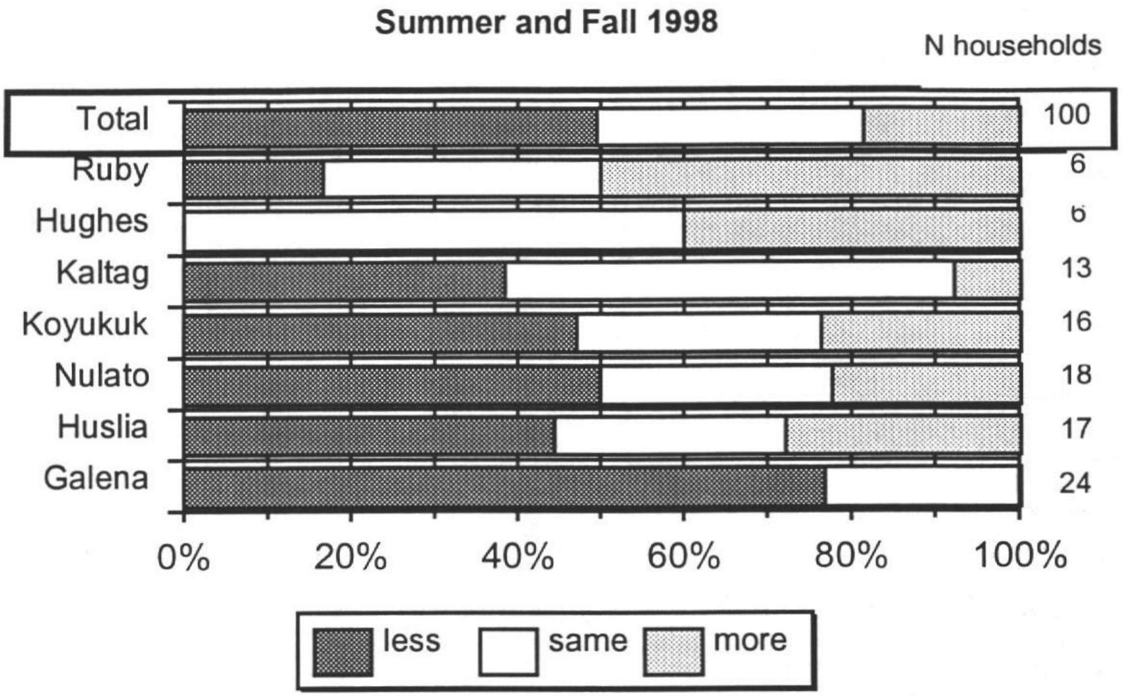

Fig. 12. Size of waterfowl harvest relative to previous years as estimated by households in seven communities in the Koyukon region, 1998. Estimates for Galena are based on a 50% random sample.

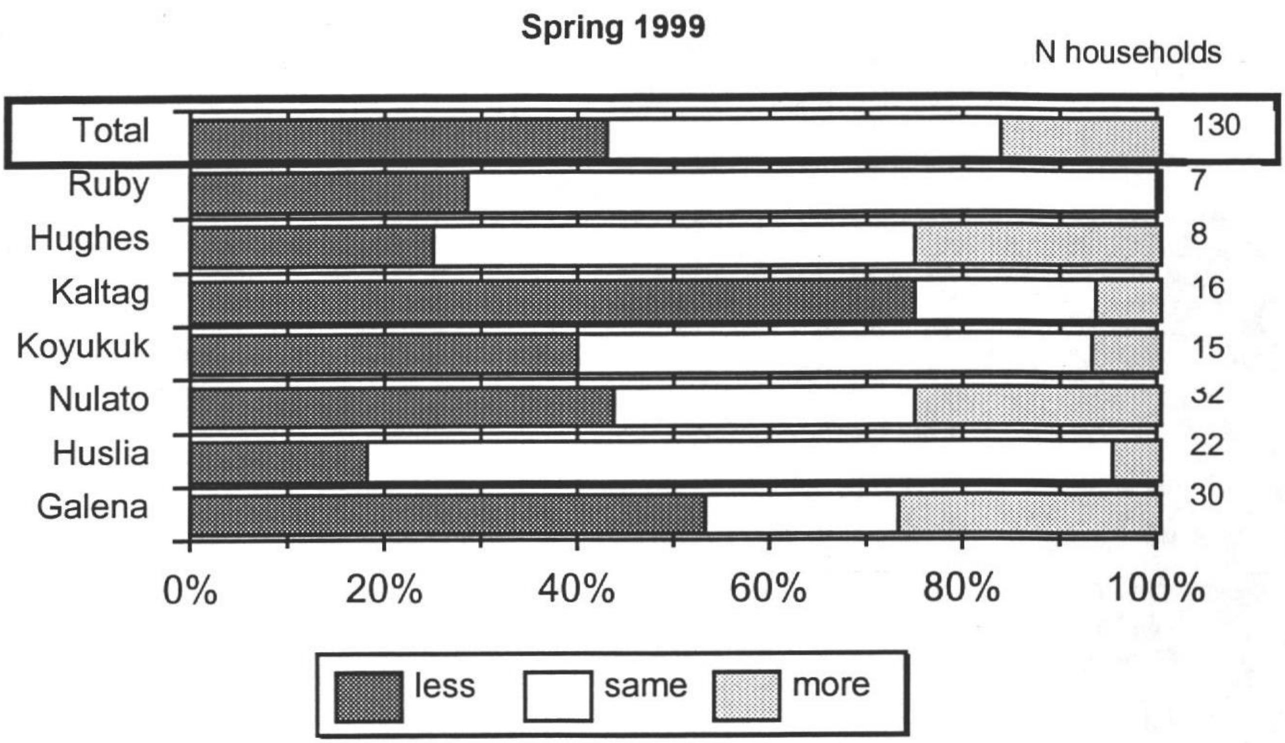

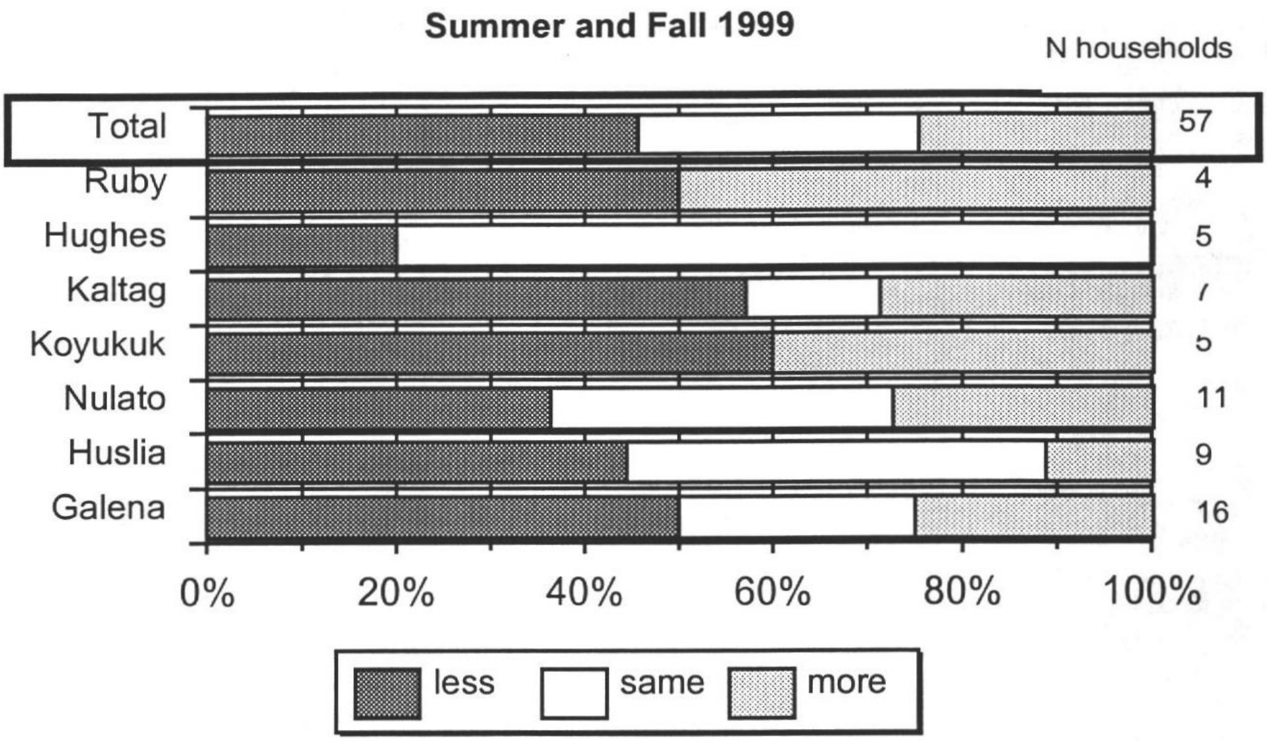

Fig. 13. Size of waterfowl harvest relative to previous years as estimated by households in seven communities in the Koyukon region, 1999. Estimates for Galena are based on a 50% random sample.

Table 1. Participation in the subsistence waterfowl harvest survey in the Koyukon region in 1998 and in 1999, based on interviews of all occupied households (HH), except Galena, which was surveyed using a 50% random sample.

**1998**		Galena	Huslia	Nulato	Koyukuk	Kaltag	Hughes
**Spring**	*Number of HH occupied*	190	62	88	40	55	27
	Number of HH interviewed	95	53	71	35	46	13
	Percent of HH interviewed	50	85	81	88	84	48
	Population covered by survey	275	162	261	90	173	46
	*Population AK Dept. of Labor**	544	248	353	130	250	52
**Fall**	*Number of HH occupied*	190	56	85	41	59	23
	Number of HH interviewed	95	55	81	31	58	20
	Percent of HH interviewed	50	98	95	76	98	87
	Population covered by survey	271	168	309	79	214	58
	*Population AK Dept. of Labor **	544	248	353	130	250	52

* Alaska population, AK Dept. of Labor, Research and Analysis Section, Demographics Unit, July 1998

**1999**		Galena	Huslia	Nulato	Koyukuk	Kaltag	Hughes
**Spring**	*Number of HH occupied*	198	63	88	40	55	22
	Number of HH interviewed	99	52	83	32	53	18
	Percent of HH interviewed	50	83	94	80	96	82
	Population covered by survey	267	157	322	81	207	69
	*Population AK Dept. of Labor***	563	272	381	101	254	61
**Fall**	*Number of HH occupied*	198	61	81	44	57	23
	Number of HH interviewed	99	60	75	34	56	23
	Percent of HH interviewed	50	98	93	77	98	100
	Population covered by survey	291	186	278	82	218	64
	*Population AK Dept. of Labor ***	563	272	381	101	254	61

** Alaska population, AK Dept. of Labor, Research and Analysis Section, Demographics Unit, July 1999

Table 2. Waterfowl harvest by seven communities in the Koyukon region by species and by season, 1998, based on household interviews. Estimates for Galena are expanded from a 50% random sample.

	Galena		Huslia		Nulato		Koyukuk		Kaltag		Hughes		Ruby		Total	
	Spring	Fall	Spring	Fall	Spring	Fall	Spring	Fall	Spring	Fall	Spring	Fall	Spring	Fall	Spring	Fall
Number of households interviewed	95	95	53	55	71	81	35	31	46	58	13	20	48	51	361	391
Number of households occupied	190	190	62	56	88	85	40	41	55	59	27	23	57	59	518	512
**Species**																
Swans	-	-	1	-	-	-	1	1	1	-	-	-	-	-	3	1
Cranes																
Sandhill Crane	4	6	1	-	-	4	3	-	6	-	-	-	-	-	14	10
Geese																
Black Brant	-	-	-	-	-	-	-	-	-	-	-	-	-	-	-	-
Canada Goose	86	34	108	29	56	26	113	24	93	7	72	2	25	6	553	128
White-fronted Goose	36	10	109	10	34	4	57	4	23	-	19	-	7	-	285	28
Snow Goose	-	2	-	4	-	-	-	-	1	-	-	-	-	-	1	6
unidentified	4	-	6	10	13	27	6	5	4	-	5	-	-	-	38	42
total	126	46	223	53	103	57	176	33	121	7	96	2	32	6	877	204
Ducks																
American Widgeon	94	84	18	67	8	9	45	26	13	7	16	-	6	-	200	193
Bufflehead	2	4	-	-	-	-	1	-	1	-	80	-	-	2	84	6
Canvasback	-	-	-	-	-	1	-	-	-	-	-	-	-	-	-	1
Goldeneye	2	-	-	-	-	2	-	-	-	2	-	-	1	-	3	4
Green-winged Teal	8	22	5	6	1	12	1	-	1	2	5	1	2	-	23	43
Mallard	56	58	11	60	37	64	20	24	11	31	40	14	35	12	210	263
Northern Pintail	22	24	19	14	17	21	37	7	4	2	54	3	11	15	164	86
Northern Shoveler	20	8	-	-	5	-	1	5	8	2	10	-	3	-	47	15
Oldsquaw	-	-	5	5	-	-	1	-	-	-	30	-	-	-	36	5
Ring-necked duck	-	-	-	-	-	-	-	-	-	-	-	-	-	-	-	-
Scaup	22	6	-	-	-	-	-	-	-	-	-	-	-	-	22	6
Scoter	-	-	11	24	1	1	-	-	-	5	6	-	-	-	18	30
unidentified	10	-	4	8	-	32	4	30	5	36	15	8	1	7	39	121
total	236	258	73	184	69	142	110	92	43	87	256	26	59	36	846	773
others																
Merganser	-	-	-	-	2	-	-	-	1	-	-	-	-	-	3	-
unidentified waterfowl	-	-	-	-	2	-	-	-	-	-	-	-	-	-	2	-
**Grand total**	366	258	298	237	176	203	290	126	172	94	352	28	91	42	1745	988

Table 3. Waterfowl harvest by seven communities in the Koyukon region by species and by season, 1999, based on household interviews. Estimates for Galena are expanded from a 50% random sample.

	Galena		Huslia		Nulato		Koyukuk		Kaltag		Hughes		Ruby		Total	
	Spring	Fall	Spring	Fall	Spring	Fall	Spring	Fall	Spring	Fall	Spring	Fall	Spring	Fall	Spring	Fall
Number of households interviewed	*99*	*99*	*52*	*60*	*83*	*75*	*32*	*34*	*53*	*56*	*18*	*23*	*56*	*56*	*393*	*403*
Number of households occupied	*198*	*198*	*63*	*61*	*88*	*81*	*40*	*44*	*55*	*57*	*22*	*23*	*65*	*64*	*531*	*528*
**Species**																
Swans	-	-	-	-	-	-	-	-	-	-	-	-	-	-	-	-
Cranes																
Sandhill Crane	2	-	-	-	-	-	-	-	-	-	-	-	1	-	*3*	-
Geese																
Black Brant	-	-	6	-	-	-	-	-	-	-	-	-	-	-	*6*	-
Canada Goose	78	36	87	22	111	17	61	13	69	16	20	10	31	2	*457*	*116*
White-fronted Goose	34	-	123	14	147	26	88	11	56	-	29	8	1	1	*478*	*60*
Snow Goose	2	-	-	-	-	-	1	-	12	-	-	-	2	-	*17*	-
unidentified	-	-	10	-	-	9	-	-	-	8	23	-	-	-	*33*	*17*
total	114	36	226	36	258	52	150	24	137	24	72	18	34	3	*991*	*193*
Ducks																
American Widgeon	66	46	14	-	16	12	2	5	-	-	17	-	7	-	*122*	*63*
Bufflehead	-	8	-	-	-	-	5	-	-	-	-	-	1	-	*6*	*8*
Canvasback	-	-	-	-	2	-	-	-	-	-	-	-	-	-	*2*	-
Goldeneye	-	-	-	-	2	-	-	-	-	-	-	-	3	-	*5*	-
Green-winged Teal	2	8	3	-	5	7	3	1	-	-	-	-	-	-	*13*	*16*
Mallard	58	6	13	12	43	12	18	16	25	6	24	18	25	16	*206*	*86*
Northern Pintail	4	12	18	14	35	3	15	-	6	-	11	12	18	11	*107*	*52*
Northern Shoveler	2	-	-	-	5	-	3	-	2	-	-	-	-	-	*12*	-
Oldsquaw	-	-	2	4	-	-	2	-	-	-	-	-	-	-	*4*	*4*
Ring-necked duck	-	-	-	-	4	-	-	-	-	-	-	-	-	-	*4*	-
Scaup	-	-	-	-	-	-	-	-	-	-	-	-	-	-	-	-
Scoter	-	-	3	-	3	-	1	3	-	-	2	10	-	-	*9*	*13*
unidentified	-	-	1	-	47	34	33	8	-	10	-	-	-	12	*81*	*64*
															-	-
total	132	80	54	30	162	68	82	33	33	16	54	40	54	39	*571*	*306*
others																
Merganser	-	-	-	-	-	-	-	-	-	-	-	-	-	-	-	-
unidentified waterfowl	-	-	-	-	-	-	-	-	-	-	-	-	-	-	-	-
**Grand total**	248	116	280	66	420	120	232	57	170	40	126	58	89	42	*1565*	*499*

Table 4. Rank of waterfowl species abundance compared to rank of species harvested. Mean abundance (in thousands) calculated from annual duck breeding population estimates (1984-1999, Table 5) for the Koyukuk stratum (4,100 mi^2), including Koyukuk and Kanuti NWRs, based on aerial breeding pair survey, USFWS, Migratory Birds, Juneau, AK. Harvest numbers based on household interviews in the Koyukon region.

Species	*Mean Abundance*	*Ranking*		
		Abundance	*Harvest 1998*	*Harvest 1999*
American wigeon	*43.3*	1	2	2
Bufflehead	*4.0*	8	4	6
Canvasback	*1.4*	12	12	11
Goldeneye	*3.6*	9	10	9
Green-winged teal	*23.6*	4	5	4
Mallard	*19.5*	5	1	1
Northern pintail	*38.3*	2	3	3
Northern shoveler	*17.9*	6	6	7
Oldsquaw	*1.8*	11	8	8
Ring-necked duck	*2.6*	10	-	10
Scaup	*31.2*	3	9	-
Scoters	*6.0*	7	7	5
Merganser	*0.2*	13	11	-

Table 5. Abundance of waterfowl species (in thousands, mean and coefficient of variation) in the Koyukuk stratum (4,100 mi ^{2}), including Koyukuk and Kanuti NWRs, based on annual aerial breeding population survey, USFWS, Migratory Birds, Juneau. Means are calculated from annual estimates in 1984-1999.

Species	***Mean***	***CV***	***1998***	***1999***
American wigeon	*43.3*	*0.31*	63.6	36.1
Bufflehead	*4.0*	*0.24*	3	2.9
Canvasback	*1.4*	*0.88*	0.2	0
Goldeneye	*3.6*	*0.55*	3	3
Green-winged teal	*23.6*	*0.38*	30	24.6
Mallard	*19.5*	*0.40*	41.2	19
Northern pintail	*38.3*	*0.47*	25.7	25.5
Northern shoveler	*17.9*	*0.37*	32.6	19.2
Oldsquaw	*1.8*	*0.75*	0.6	0.6
Ring-necked duck	*2.6*	*0.73*	4.7	1.2
Scaup	*31.2*	*0.30*	20.1	25.2
Scoters	*6.0*	*0.39*	4.5	3.4
Merganser	*0.2*	*1.08*	0.3	0

Table 6. Comparison of 1998 and 1999 total waterfowl subsistence harvest with estimates from previous surveys conducted in the Koyukon region (hyphen indicates no information available).

Ducks	Source	Galena	Huslia	Nulato	Koyukuk	Kaltag	Hughes	Ruby	Total *Gal-Kal***	Total *All villages*
1974	Loranger 1985	1200	800	900	1000	600	360	250	4500	5110
1982	Marcotte and Haynes, 1985	-	-	-	-	-	505	-	-	-
1983	Marcotte 1983 (Huslia), Looman 1987 (Ruby)	-	1032	-	-	-	-	391	-	-
1985	Wolfe et al. 1990 (Estimates by expanding data)	1886	1488	2184	835	1623	537	1407	8016	9960
1986	Marcotte 1990	1374	-	-	-	-	-	-	-	-
1992	Survey USFWS Galena, unpubl. data	845	234*	242	419	343	-	-	2083	-
1998	Survey USFWS Galena	494	257	211	202	130	282	95	1294	1671
1999	Survey USFWS Galena	212	84	230	115	49	94	93	690	877

Geese	Source	Galena	Huslia	Nulato	Koyukuk	Kaltag	Hughes	Ruby	Total *Gal-Kal***	Total *All villages*
1974	Loranger 1985	1000	600	200	1000	400	200	100	3200	3500
1982	Marcotte and Haynes, 1985	-	-	-	-	-	228	-	-	-
1983	Marcotte 1983 (Huslia), Looman 1987 (Ruby)	-	555	-	-	-	-	137	-	-
1985	Wolfe et al. 1990 (Estimates by expanding data)	537	800	936	364	707	258	613	3344	4215
1986	Marcotte 1990	391	-	-	-	-	-	-	-	-
1991	Estimate made for USFWS Galena, unpubl. data	-	405*	-	-	-	-	-	-	-
1992	Survey USFWS Galena, unpubl. data	295	596*	450	170	216	-	-	1727	1131
1995	Estimate made for USFWS Galena, unpubl. data	-	124*	-	-	-	-	-	-	-
1996	Estimate made for USFWS Galena, unpubl. data	-	360*	-	-	-	-	-	-	-
1997	Estimate made for USFWS Galena, unpubl. data	-	228*	-	-	-	-	-	-	-
1998	Survey USFWS Galena	172	276	160	209	128	98	38	945	1081
1999	Survey USFWS Galena	150	262	310	174	161	90	37	1057	1184

* only spring harvest estimated

** Total includes Galena, Huslia, Nulato, Koyukuk and Kaltag

Table 7. Estimated harvest of waterfowl by seven communities in the Koyukon region in 1998 and 1999, based on household interviews. Average harvest per household (hh) was calculated from the total of households recorded in the spring and fall surveys. Alaska Dept. of Labor human population data from 1998 and 1999 were used to estimate per capita harvests. Harvest estimates for Galena are expanded from a 50% random sample. Harvest was converted to edible weight (lbs) using values provided in Wentworth and Seim (1996) and Bellrose (1980).

1998

Community	N birds total	lbs birds total	N birds/capita	lbs birds/capita	N birds/hh	lbs birds/hh
Galena	624	1287	1.2	2.4	3.3	6.8
Huslia	535	1518	2.2	6.1	9.1	25.7
Nulato	379	977	1.1	2.8	4.4	11.2
Koyukuk	416	1172	3.2	9	10.2	28.6
Kaltag	266	761	1.1	3	4.7	13.4
Hughes	380	722	7.3	13.9	15.2	28.9
Ruby	133	280	0.7	1.4	2.3	4.8

1999

Community	N birds total	lbs birds total	N birds/capita	lbs birds/capita	N birds/hh	lbs birds/hh
Galena	364	887	0.6	1.6	1.8	4.5
Huslia	346	1207	1.3	4.4	5.6	19.5
Nulato	540	1693	1.4	4.4	6.4	19.9
Koyukuk	289	880	2.9	8.7	6.9	21.0
Kaltag	210	749	0.8	2.9	3.8	13.4
Hughes	184	507	3.0	8.3	8.0	22.0
Ruby	131	282	0.7	1.5	2.0	4.3

Table 8. Number of households (HH) harvesting, sharing, receiving, and using (either harvesting, sharing or receiving) waterfowl in seven communities in the Koyukon region in 1998 and in 1999. Estimates for Galena are shown as recorded and expanded from the 50% random sample.

1998

Spring	Galena	Huslia	Nulato	Koyukuk	Kaltag	Hughes	Ruby	***Total***
Number of								
HH interviewed	95 / 190	53	71	35	46	13	48	*361 / 456*
HH harvested	22 / 44	25	24	15	16	8	12	*122 / 144*
HH shared	15 / 30	22	13	14	8	5	6	*83 / 98*
HH received	15 / 30	22	18	14	15	4	5	*93 / 108*
HH used	37 / 74	44	39	25	27	10	16	*198 / 235*

Summer and Fall	Galena	Huslia	Nulato	Koyukuk	Kaltag	Hughes	Ruby	***Total***
Number of								
HH interviewed	95 / 190	55	81	31	58	20	51	*391 / 486*
HH harvested	12 / 24	16	18	16	13	5	6	*86 / 98*
HH shared	22 / 44	8	16	11	12	1	6	*76 / 98*
HH received	8 / 16	15	22	8	10	-	3	*66 / 74*
HH used	31 / 62	28	37	21	24	5	9	*155 / 186*

1999

Spring	Galena	Huslia	Nulato	Koyukuk	Kaltag	Hughes	Ruby	***Total***
Number of								
HH interviewed	99 / 198	52	83	32	53	18	56	*393 / 492*
HH harvested	13 / 26	23	30	14	18	9	7	*114 / 127*
HH shared	27 / 54	13	28	17	13	7	8	*113 / 140*
HH received	44 / 88	22	39	18	16	8	11	*158 / 202*
HH used	54 / 108	40	60	28	31	15	18	*246 / 300*

Summer and Fall	Galena	Huslia	Nulato	Koyukuk	Kaltag	Hughes	Ruby	***Total***
Number of								
HH interviewed	99 / 198	60	75	34	56	23	56	*403 / 502*
HH harvested	8 / 16	7	12	7	7	4	4	*49 / 57*
HH shared	10 / 20	6	17	8	8	3	9	*61 / 71*
HH received	18 / 36	6	24	17	10	6	9	*90 / 108*
HH used	25 / 50	13	34	23	18	8	17	*138 / 163*

Table 9. Distances from the community while hunting waterfowl and time spent hunting waterfowl by households of seven communities in the Koyukon region in 1998 and in 1999. (Figures in parentheses are percent.) Estimates for Galena are expanded from a 50% random sample.

	miles			days		
	10 and less	11-20	more than 20	1-3	4-6	more than 7
1998						
Spring	72	37	35	81	31	31
Summer and fall	33	12	56	51	17	22
1999						
Spring	57	27	55	75	37	26
Summer and fall	25	20	24	29	19	12

Appendix I

Table 1. Usable weight of waterfowl species harvested in the Koyukon region in 1998 and in 1999. Values are reported in Wentworth and Seim (1996) or were calculated from round weight listed in Bellrose (1980).

Species	*lbs usable weight*
Swans	15.08
Sandhill Crane	6.75
Black Brant	2.28
Canada Goose	4.31
White-fronted Goose	4.24
Snow Goose	3.99
unidentified	4.18
American Widgeon	0.96
Bufflehead	0.70
Canvasback	1.99
Goldeneye	1.29
Green-winged Teal	0.52
Mallard	1.43
Northern Pintail	1.13
Northern Shoveler	0.79
Oldsquaw	1.34
Ring-necked duck	1.56
Scaup	1.48
Scoter	1.88
unidentified	1.15
Merganser	1.76
unidentified waterfowl	2.66

Appendix II

Survey form spring 1998

Survey form fall 1998

Survey form spring 1999

Survey form fall 1999

Summary sheet

Hand out spring 1998

Hand out fall 1998

Hand out spring 1999

Hand out fall 1999

598

Subsistence Waterfowl Harvest Survey

Koyukuk, Nulato, Kaltag, Huslia, Hughes, Ruby, Galena

Spring to early Summer (April 15 - June 15) 1998

Community ______________

Household-Number __________

Interview date ____________

Interviewer ______________

Final Status

- ☐ harvested wf
- ☐ hunted wf but no harvest
- ☐ did not hunt wf
- ☐ could not be contacted
- ☐ did not want to be interwieved

Attempts of contact

	Date	Time	Comments
1.			
2.			
3.			

List number of birds killed and/or eggs taken by hunter.
If none, cross out chart

	# birds	# eggs		# birds	# eggs
1 Swan			13 Scaup		
2 Sandhill Crane			14 Goldeneye		
3 Lesser Snow Goose			15 White-winged Scoter		
4 White-fronted Goose			16 Black Scoter		
5 Lesser Canada Goose			17 Surf Scoter		
6 Pintail			18 *Unidentified duck*		
7 Oldsquaw			19 *Unidentified goose*		
8 Mallard			20 *Unidentified other waterfowl*		
9 Widgeon			21 *Identified other waterfowl Species:*		
10 Bufflehead					
11 Green-winged Teal			Total		
12 Shoveler					

Cross out the number of every question asked

Q1* How many people are in your household?

_________people

Q2 How many household members hunted waterfowl or gathered eggs this spring?

_________members

Q3* Did you share any harvested waterfowl with people of other households or at memorial potlaches this spring?

☐ yes ☐ no

Q4* Did you receive any waterfowl from people of other households this spring?

☐ yes ☐ no

Q5* Are any members of your household Alaskan Native?

☐ yes ☐ no

Q6* Do you consider your hunting cultural?

☐ yes ☐ no

Q7 About how much time did your household spend out hunting waterfowl?

________days

Q8 About how far away from the village were you while you were hunting waterfowl?

________miles (maximal range)

Q9* How long have you hunted waterfowl in this village?

________years

Q10* Do you feel waterfowl numbers have changed around this area since you began hunting waterfowl?

☐ yes, there are more ☐ yes, there are fewer ☐ no ☐ don't know

Comments (extent, concerned species)____

____

____

Q11* This spring, did your household harvest less, more or about the same amount of waterfowl as in the past? (*If no harvest:* Have you ever harvested waterfowl? *If yes, cross 'less'*)

☐ never harvest ☐ less ☐ same ☐ more

If less or more, why?____

____

____

Comments* (Conditions of animals taken, hunter concerns, observations on new species and migration routes, weather conditions, memorial potlaches, on the survey)

____

____

____

____

____

____

Reviewed by________________

US Fish and Wildlife Service, PO Box 287, Galena AK 99741, (907) 656-1231

+98

Subsistence Waterfowl Harvest Survey

Koyukuk, Nulato, Kaltag, Huslia, Hughes, Ruby, Galena

Summer and Fall Harvest (May 20th to September 25th)

Community ________________

Household-Number ____________

Interview date ______________

Interviewer ________________

Final Status

☐ harvested wf ☐ could not be contacted

☐ hunted wf but no harvest ☐ did not want to be interviewed

☐ did not hunt wf

Attempts of contact

	Date	Time	Comments
1.			
2.			
3.			

List number of birds killed and/or eggs taken by all hunters of household from May 20th to September 25th
('from when school was out until the close of moose hunting season')
If none, cross out chart
Harvest calendar filled out? Yes__________ No__________

	# birds	# eggs
1 Swan		
2 Sandhill Crane		
3 Lesser Snow Goose		
4 White-fronted Goose		
5 Lesser Canada Goose		
6 Pintail		
7 Oldsquaw		
8 Mallard		
9 Widgeon		
10 Bufflehead		
11 Green-winged Teal		
12 Shoveler		

	# birds	# eggs
13 Scaup		
14 Goldeneye		
15 White-winged Scoter		
16 Black Scoter		
17 Surf Scoter		
18 *Unidentified duck*		
19 *Unidentified goose*		
20 *Unidentified other waterfowl*		
21 *Identified other waterfowl Species:*		
**Total**		

Cross out the number of every question asked
If a household harvested waterfowl ask all the questions, if a household did not harvest waterfowl only ask the questions with a star.

Q1* **How many people are in your household?**

_________ people

Q2 **How many household members hunted ducks and geese this summer and fall?**

_________ members

Q3* **Did you share any harvested ducks and geese with people of other households or at memorial potlaches this summer and fall?**

☐ yes ☐ no

Q4* **Did you receive any ducks and geese from people of other households this summer and fall?**

☐ yes ☐ no

Q5 **About how much time did your household spend out hunting ducks and geese?**

________days

Q6 **About how far away from the village were you while you were hunting ducks and geese?**
Try to get a straight-line distance guess and note the maximal range if the person was out hunting several times

________miles

Q7 **How many ducks and geese did your household harvest this summer or fall compared to previous years?**

☐ about the same amount

☐ more than in previous years

☐ fewer than in previous years

If fewer or more, why?____

____

Q8* **When does your household generally harvest ducks and geese?**
More than one answer possible

☐ spring

☐ summer

☐ fall

☐ our household does not harvest ducks and geese
(e.g., has never harvested, does not harvest anymore)

Comments* (Conditions of animals taken, hunter concerns, observations on ducks and geese, weather conditions, memorial potlaches, on the survey)

____

____

____

____

____

Reviewed by________________

199

Subsistence Waterfowl Harvest Survey
Koyukuk, Nulato, Kaltag, Huslia, Hughes, Ruby, Galena
Spring Harvest 1999 (April 1st to May 20th)

Community ____________

Household-Number ____________

Interview date ____________

Interviewer ____________

Final Status

- ☐ **harvested wf**
- ☐ **could not be contacted**
- ☐ **hunted wf but no harvest**
- ☐ **did not want to be interviewed**
- ☐ **did not hunt wf**

Attempts of contact

	Date	Time	Comments
1.			
2.			
3.			

List number of birds killed and/or eggs taken by all hunters of household from April 1st to May 20th ('from the birds' arrival until the school was out')
If none, cross out chart
Harvest calendar filled out? Yes__________ No__________

	# birds	# eggs		# birds	# eggs
1 Swan			13 Scaup		
2 Sandhill Crane			14 Goldeneye		
3 Lesser Snow Goose			15 White-winged Scoter		
4 White-fronted Goose			16 Black Scoter		
5 Lesser Canada Goose			17 Surf Scoter		
6 Pintail			18 *Unidentified duck*		
7 Oldsquaw			19 *Unidentified goose*		
8 Mallard			20 *Unidentified other waterfowl*		
9 Widgeon			21 *Identified other waterfowl Species:*		
10 Bufflehead					
11 Green-winged Teal			Total		
12 Shoveler					

Cross out the number of every question asked
If a household harvested waterfowl ask all the questions, if a household did not harvest waterfowl only ask the questions with a star.

Q1* How many people are in your household?

________people

Q2 How many household members hunted ducks and geese this spring?

________members

Q3* Did you share any harvested ducks and geese with people of other households or at memorial potlaches this spring?

☐ yes ☐ no

Q4* **Did you receive any ducks and geese from people of other households this spring?**

☐ yes ☐ no

Q5 **About how much time did your household spend out hunting ducks and geese?**

________days

Q6 **About how far away from the village were you while you were hunting ducks and geese?**
Try to get a straight-line distance guess and note the maximal range if the person was out hunting several times

________miles

Q7 **How many ducks and geese did your household harvest this spring compared to previous years?**

☐ about the same amount

☐ more than in previous years

☐ fewer than in previous years

If fewer or more, why?____

____

Q8* **When does your household generally harvest ducks and geese?**
More than one answer possible

☐ spring

☐ summer

☐ fall

☐ our household does not harvest ducks and geese
(e.g., has never harvested, does not harvest anymore)

Comments* (Conditions of animals taken, hunter concerns, observations on ducks and geese, weather conditions, memorial potlaches, on the survey)

____

____

____

____

____

Reviewed by ________________

+99

Subsistence Waterfowl Harvest Survey

Koyukuk, Nulato, Kaltag, Huslia, Hughes, Ruby, Galena

Summer and Fall Harvest 1999 (June 1st to October 4th)

Community ________________

Household-Number ____________

Interview date ______________

Interviewer________________

Final Status

- ☐ harvested wf
- ☐ hunted wf but no harvest
- ☐ did not hunt wf
- ☐ could not be contacted
- ☐ did not want to be interviewed

Attempts of contact

	Date	Time	Comments
1.			
2.			
3.			

List number of birds killed and/or eggs taken by all hunters of household from June 1st to October 4th ('one week after school was out until one week after close of moose hunting season')
If none, cross out chart

	# birds	# eggs
1 Swan		
2 Sandhill Crane		
3 Lesser Snow Goose		
4 White-fronted Goose		
5 Lesser Canada Goose		
6 Pintail		
7 Oldsquaw		
8 Mallard		
9 Widgeon		
10 Bufflehead		
11 Green-winged Teal		
12 Shoveler		

	# birds	# eggs
13 Scaup		
14 Goldeneye		
15 White-winged Scoter		
16 Black Scoter		
17 Surf Scoter		
18 *Unidentified duck*		
19 *Unidentified goose*		
20 *Unidentified other waterfowl*		
21 *Identified other waterfowl Species:*		
Total		

Cross out the number of every question asked
If a household harvested waterfowl ask all the questions, if a household did not harvest waterfowl only ask the questions with a star.

Q1* **How many people are in your household?**

_________people

Q2 **How many household members hunted ducks and geese this summer and fall?**

_________members

Q3* **Did you share any harvested ducks and geese with people of other households or at memorial potlaches this summer and fall?**

☐ yes ☐ no

Q4* **Did you receive any ducks and geese from people of other households or at potlatches this summer and fall?**

☐ yes ☐ no

Q5 **About how much time did your household spend out hunting ducks and geese?**

_________days

Q6 **About how far away from the village were you while you were hunting ducks and geese?**
Try to get a straight-line distance guess and note the maximal range if the person was out hunting several times

_________miles

Q7 **How many ducks and geese did your household harvest this summer and fall compared to previous years?**

☐ about the same amount

☐ more than in previous years

☐ fewer than in previous years

If fewer or more, why? ____

____

Q8* **When does your household generally harvest ducks and geese?**
More than one answer possible,

☐ spring

☐ summer

☐ fall

☐ our household does not harvest ducks and geese
(e.g., has never harvested, does not harvest anymore)

Months____

Comments* (Conditions of animals taken, hunter concerns, observations on ducks and geese, weather conditions, memorial potlaches, on the survey)

____

____

____

____

____

Reviewed by________________

Subsistence Waterfowl Harvest Survey - Summary Sheet

Survey Period:____________________ Community:____________________

Surveyor(s):____________________ Total number of households: ________________

Record contact dates for each household and write final status down

001 ________	026 ________	051 ________	076 ________
002 ________	027 ________	052 ________	077 ________
003 ________	028 ________	053 ________	078 ________
004 ________	029 ________	054 ________	079 ________
005 ________	030 ________	055 ________	080 ________
006 ________	031 ________	056 ________	081 ________
007 ________	032 ________	057 ________	082 ________
008 ________	033 ________	058 ________	083 ________
009 ________	034 ________	059 ________	084 ________
010 ________	035 ________	060 ________	085 ________
011 ________	036 ________	061 ________	086 ________
012 ________	037 ________	062 ________	087 ________
013 ________	038 ________	063 ________	088 ________
014 ________	039 ________	064 ________	089 ________
015 ________	040 ________	065 ________	090 ________
016 ________	041 ________	066 ________	091 ________
017 ________	042 ________	067 ________	092 ________
018 ________	043 ________	068 ________	093 ________
019 ________	044 ________	069 ________	094 ________
020 ________	045 ________	070 ________	095 ________
021 ________	046 ________	071 ________	096 ________
022 ________	047 ________	072 ________	097 ________
023 ________	048 ________	073 ________	098 ________
024 ________	049 ________	074 ________	099 ________
025 ________	050 ________	075 ________	100 ________

Explain households that did not want to be interviewed/could not be contacted (why, were they around during this spring/summer and fall, were they likely to be waterfowl hunters):

____

____

____

____

____

Additional comments about this survey period (weather, memorial potlaches and funerals, etc.):

____

____

Subsistence Waterfowl Harvest Survey
Koyukuk, Nulato, Kaltag, Huslia, Hughes, Ruby, Galena
Spring and Fall 1998

•In May and June, and again in late September 1998, the U.S. Fish and Wildlife Service will conduct household interviews to gather information on subsistence hunting and use of waterfowl. This survey was approved by the local tribal councils and will be done in Galena, Ruby, Koyukuk, Nulato, Kaltag, Huslia and Hughes. Similar surveys have been done on the Yukon Flats, the Yukon-Kuskokwim Delta and in the Bering Strait region.

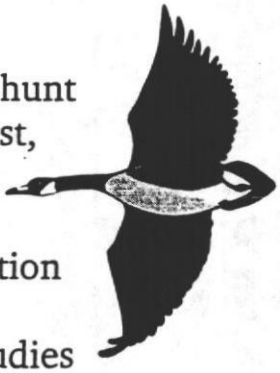

•The harvest survey will show us which waterfowl species people hunt and depend on. This information will be used to assess local harvest, and develop spring hunting according to the Migratory Bird Treaty Act amendments. Float and aerial surveys and elder interviews have showed that the Interior-Northwest Alaska population of white-fronted geese (speckled bellies, orange-footed geese) has declined, and information about local harvest will contribute to studies to find out why these geese are declining.

•Each interview will take about ten minutes. We will ask about numbers and species of the waterfowl taken by you or your household in spring and again in fall. We will also ask questions about traditional hunting and use of waterfowl, and any changes noted of waterfowl populations. The surveyor will be a local-hire employee from the region, who may be with another U.S. Fish and Wildlife Service employee.

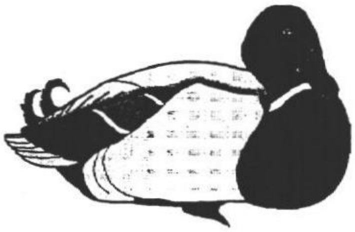

•The U.S Fish and Wildlife Service will not record the names of people who participate. The information will be collected anonymously and will not be used for any enforcement action.

•Participation in the survey is voluntary. Households may decline to answer any questions they wish.

•Information collected from this survey and from our float and aerial surveys will be provided to local tribal and city councils for community review.

If you have any questions about this project, you can call:
Mike Spindler or Deborah Webb at 1-800-656-1231, or mail:

U.S. Fish and Wildlife Service
PO Box 287
Galena, AK 99741

Waterfowl Harvest Calendar

Household-Number ____________

Please write down how many birds and/or eggs of each species people of your household harvested

Species	May (from 20th)	June	July	August	September (until 20th)	*egg count*
Swan						
Sandhill Crane						
Lesser Snow Goose						
White-fronted Goose						
Lesser Canada Goose						
Pintail						
Oldsquaw						
Mallard						
Widgeon						
Bufflehead						
Green-winged Teal						
Shoveler						
Scaup						
Goldeneye						
White-winged Scoter						
Black Scoter						
Surf Scoter						
Common Loon						
Arctic Loon						
Red-throated Loon						
Red-necked Grebe						
Black Brant						
Large Shorebird						
Small Shorebird						
Herring Gull						
Mew Gull						
Arctic Tern						
Common Merganser						
Red-breasted Merganser						
Redhead						
Canvasback						
Steller's Eider						
Harlequin Duck						
Ring-necked Duck						

Subsistence Waterfowl Harvest Survey
Koyukuk, Nulato, Kaltag, Huslia, Hughes, Ruby, Galena

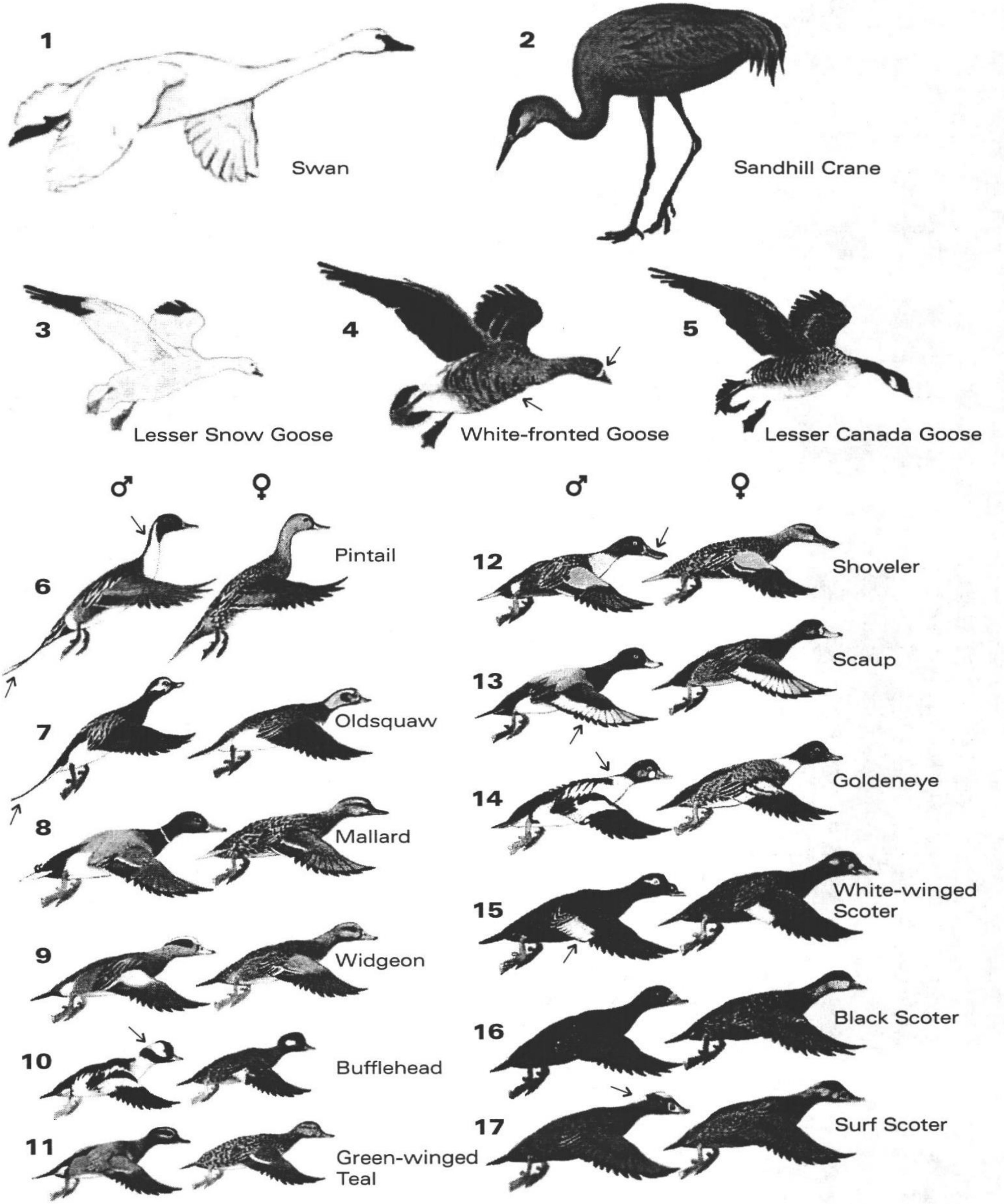

US Fish and Wildlife Service, PO Box 287, Galena AK 99741, (907) 656-1231

April 1998

Subsistence Waterfowl Harvest Survey
Koyukuk, Nulato, Kaltag, Huslia, Hughes, Ruby, Galena

Supplementary Bird Identification Sheet

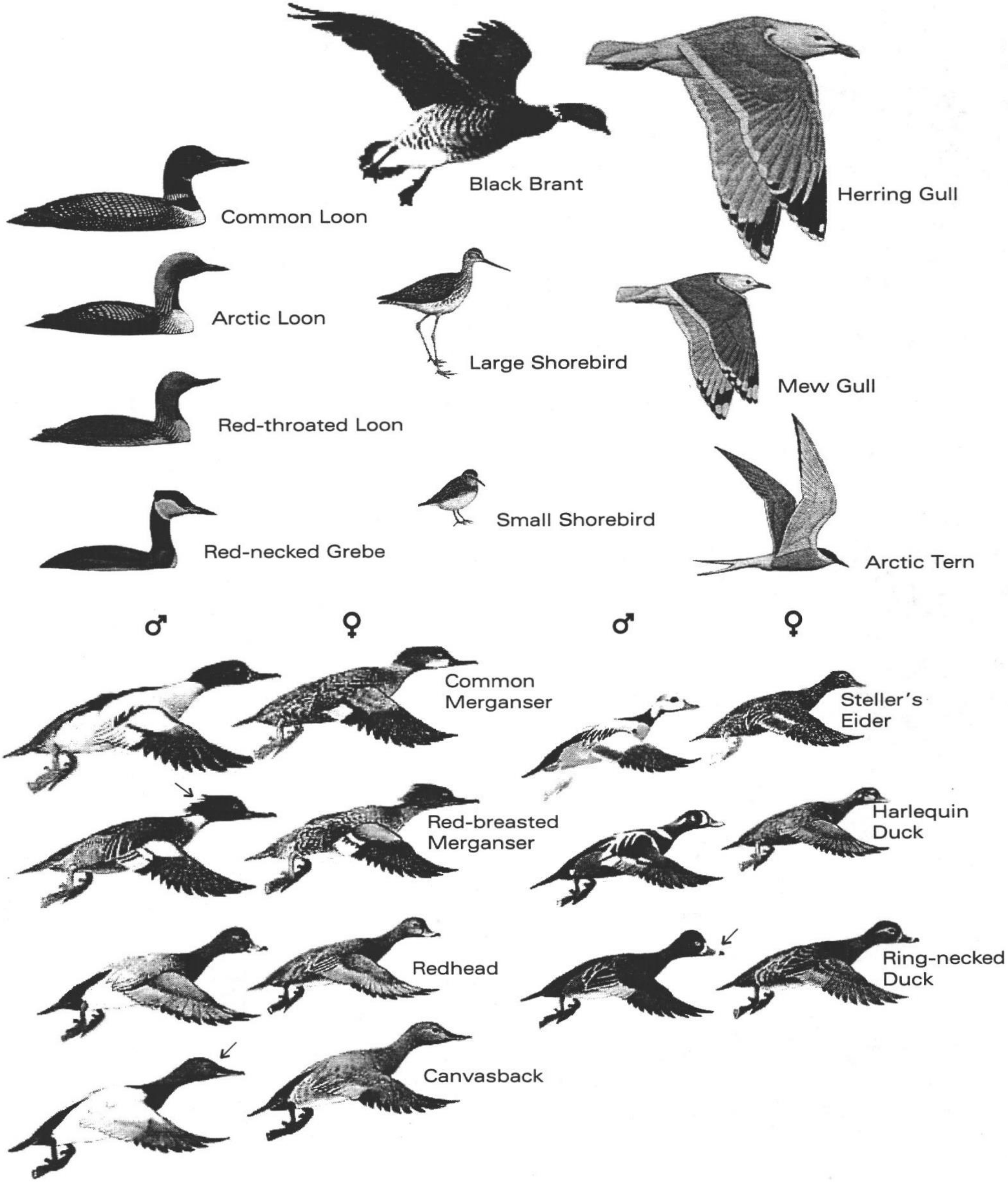

Subsistence Waterfowl Harvest Survey
Koyukuk, Nulato, Kaltag, Huslia, Hughes, Ruby, Galena
Spring and Fall 1998

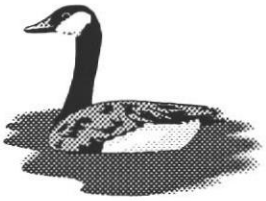

Last May and June we interviewed many people in your community about their household's waterfowl harvest. In Koyukuk, Nulato, Kaltag, Huslia, Hughes, and Ruby, we tried to talk to somebody from every household; in Galena we randomly selected half of the households and doubled the records to get an estimate for the whole community. A big thank you to everybody who participated! On the back of this page there is a chart with the numbers of ducks and geese harvested in each of the seven communities.

This survey was done by the U.S. Fish and Wildlife Service in Galena with the approval of the local tribal councils. Similar surveys have been done on the Yukon Flats, the Yukon-Kuskokwim Delta and in the Bering Strait region. We repeat the survey now to determine summer and fall harvest. We plan on doing the surveys twice a year for two more years.

We do these surveys for two main reasons:

1. To learn which kinds of ducks and geese people hunt and depend on, and how many are harvested. The recent Migratory Bird Treaty Act Amendments will allow hunting of waterfowl in the spring, and information about local subsistence harvest will help us develop regulations.

2. To contribute to studies about the white-fronted geese (speckle bellies, orange-footed geese). Elder interviews and float and aerial surveys have shown that these geese have been declining in Interior Alaska since 1983. In 1990 we counted 1,722 adult white-fronted geese along Dulbi Slough, and in 1998 only 112.

Each interview takes about ten minutes. We ask about the kinds and numbers of ducks and geese taken. We also ask some questions about your hunting and use of ducks and geese. The surveyor is a local-hire employee from the region or another U.S. Fish and Wildlife Service employee together with a local resident from your community.

We do not record the names of people who participate. The information is collected anonymously and will not be used for any enforcement action.

Participation in the survey is voluntary. Households may decline any answer they wish.

Information collected from the harvest survey and from our float and aerial surveys is continuously provided to local tribal and city councils for community review.

Information from the spring subsistence waterfowl harvest survey

Numbers of waterfowl harvested from April 1st to May 20th 1998

	Galena*	Huslia	Nulato	Koyukuk	Kaltag	Hughes	Ruby	Total
Number of households interviewed	*95*	*53*	*71*	*35*	*46*	*13*	*48*	*361*
Total number of households	*189*	*62*	*88*	*40*	*55*	*27*	*57*	*518*
**Swans**	-	1	-	1	1	-	-	3
**Cranes**	4	1	-	3	6	-	-	14
**Geese**	126	223	103	176	121	96	32	877
**Ducks**	236	73	69	110	43	256	59	846
other/unidentified waterfowl	-	-	4	-	1	-	-	5
**Grand total**	366	298	176	290	172	352	91	1745

*Harvest numbers for Galena are doubled from harvest records from half of all households

---

Did the bird you harvested wear a band or a collar?

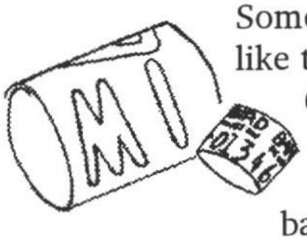

Some people reported that they had harvested banded or collared birds. We would like to encourage everybody to turn in bands or collars or to give us a call (1-800-656-1231) and tell us the number on the band or collar and when and where the bird was harvested. Our ducks and geese spend the winter season in the Lower 48 or in Mexico and South America. Recapture of banded birds at banding stations, observations of collared birds, and band or collar recoveries give us valuable information on the flight paths our birds take on their way down south.

Thank you for your help!

If you have any questions about the waterfowl harvest survey or bird bands and collars, please call:
Deborah Webb or Mike Spindler at 1-800-656-1231, or mail:

U.S. Fish and Wildlife Service
PO Box 287
Galena, AK 99741

Subsistence Waterfowl Harvest Survey
Koyukuk, Nulato, Kaltag, Huslia, Hughes, Ruby, Galena
1998/1999

Last year in the spring and again in the fall we interviewed many people in your community about their household's waterfowl harvest. In Koyukuk, Nulato, Kaltag, Huslia, Hughes, and Ruby, we tried to talk to somebody from every household; in Galena we randomly selected half of the households and doubled the records to get an estimate for the whole community. A big thank you to everybody who participated! On the back of this page there is a chart with the numbers of ducks and geese harvested in each of the seven communities.

This survey was done by the U.S. Fish and Wildlife Service in Galena with the approval of the local tribal councils. Similar surveys have been done on the Yukon Flats, the Yukon-Kuskokwim Delta and in the Bering Strait region. We plan on doing the surveys twice a year for two more years.

We do these surveys for two main reasons:
1. To learn which kinds of ducks and geese people hunt and depend on, and how many are harvested. The recent Migratory Bird Treaty Act Amendments will allow hunting of waterfowl in the spring, and information about local subsistence harvest will help us develop regulations.
2. To contribute to studies about the white-fronted geese (speckle bellies, orange-footed geese). Elder interviews and float and aerial surveys have shown that these geese have been declining in Interior Alaska since 1983. In 1990 we counted 1,722 adult white-fronted geese along Dulbi Slough, and in 1998 only 112.

Each interview takes about ten minutes. We ask about the kinds and numbers of ducks and geese taken. We also ask some questions about your hunting and use of ducks and geese. The surveyor is a local-hire employee from the region or another U.S. Fish and Wildlife Service employee together with a local resident from your community.

We do not record the names of people who participate. The information is collected anonymously and will not be used for any enforcement action.

Participation in the survey is voluntary. Households may decline any answer they wish.

Information collected from the harvest survey and from our float and aerial surveys is continuously provided to local tribal and city councils for community review.

Information from the 1998 subsistence waterfowl harvest survey

	Galena		Huslia		Nulato		Koyukuk		Kaltag		Hughes		Ruby		Total	
	Spring	*Fall*	*Spring*	*Fall*	*Spring*	*Fall*	*Spring*	*Fall*	*Spring*	*Fall*	*Spring*	*Fall*	*Spring*	*Fall*	*Spring*	*Fall*
Number of households intervi		95	53	55	71	81	35	31	46	58	13	20	48	51	361	391
Number of households occup	190	190	62	56	88	85	40	41	55	59	27	23	57	59	518	512
**Species and numbers take**																
Swans	-	-	1	-	-	-	1	1	1	-	-	-	-	-	3	1
Cranes																
Sandhill Crane	4	6	1	-	-	4	3	-	6	-	-	-	-	-	14	10
Geese																
Canada Goose	86	34	108	29	56	26	113	24	93	7	72	2	25	6	553	128
White-fronted Goose	36	10	109	10	34	4	57	4	23	-	19	-	7	-	285	28
Snow Goose	-	2	-	4	-	-	-	-	1	-	-	-	-	-	1	6
unidentified	4	-	6	10	13	27	6	5	4	-	5	-	-	-	38	42
total	126	46	223	53	103	57	176	33	121	7	96	2	32	6	877	204
Ducks																
American Widgeon	94	84	18	67	8	9	45	26	13	7	16	-	6	-	200	193
Bufflehead	2	4	-	-	-	-	1	-	1	-	80	-	-	2	84	6
Canvasback	-	-	-	-	-	1	-	-	-	-	-	-	-	-	-	1
Goldeneye	2	-	-	-	-	2	-	-	-	2	-	-	1	-	3	4
Green-winged Teal	8	22	5	6	1	12	1	-	1	2	5	1	2	-	23	43
Mallard	56	58	11	60	37	64	20	24	11	31	40	14	35	12	210	263
Northern Pintail	22	24	19	14	17	21	37	7	4	2	54	3	11	15	164	86
Northern Shoveler	20	8	-	-	5	-	1	5	8	2	10	-	3	-	47	15
Oldsquaw	-	-	5	5	-	-	1	-	-	-	30	-	-	-	36	5
Scaup	22	6	-	-	-	-	-	-	-	-	-	-	-	-	22	6
Scoter	-	-	11	24	1	1	-	-	-	5	6	-	-	-	18	30
unidentified	10	-	4	8	-	32	4	30	5	36	15	8	1	7	39	121
total	236	258	73	184	69	142	110	92	43	87	256	26	59	36	846	773
others																
Merganser	-	-	-	-	2	-	-	-	1	-	-	-	-	-	3	-
unidentified waterfowl	-	-	-	-	2	-	-	-	-	-	-	-	-	-	2	-
**Grand total**	366	258	298	237	176	203	290	126	172	94	352	28	91	42	1745	988

Harvest numbers for Galena are doubled from harvest records from half of all households

Did the bird you harvested wear a band or a collar?

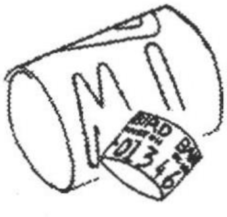

Some people reported that they had harvested banded or collared birds. We would like to encourage everybody to turn in bands or collars or to give us a call (1-800-656-1231) and tell us the number on the band or collar and when and where the bird was harvested. Our ducks and geese spend the winter season in the Lower 48 or in Mexico and South America. Recapture of banded birds at banding stations, observations of collared birds, and band or collar recoveries give us valuable information on the flight paths our birds take on their way down south.

Thank you for your help!

If you have any questions about the waterfowl harvest survey or bird bands and collars, please call:
Deborah Webb or Mike Spindler at 1-800-656-1231, or mail:
U.S. Fish and Wildlife Service, PO Box 287, Galena, AK 99741

Subsistence Waterfowl Harvest Survey
Koyukuk, Nulato, Kaltag, Huslia, Hughes, Ruby, Galena
Spring and Fall 1999

This spring again we interviewed many people in your community about their household's waterfowl harvest. In Koyukuk, Nulato, Kaltag, Huslia, Hughes, and Ruby, we tried to talk to somebody from every household; in Galena we randomly selected half of the households and doubled the records to get an estimate for the whole community. A big thank you to everybody who participated! On the back of this page there is a chart with the numbers of ducks and geese harvested this spring and for comparison also the numbers harvested in the spring of 1998 in each of the seven communities.

This survey was done by the U.S. Fish and Wildlife Service in Galena with the approval of the local tribal councils. Similar surveys have been done on the Yukon Flats, the Yukon-Kuskokwim Delta and in the Bering Strait region. We repeat the survey now to determine another year's summer and fall harvest.

We do these surveys for two main reasons:

1. To learn which kinds of ducks and geese people hunt and depend on, and how many are harvested. The recent Migratory Bird Treaty Act Amendments will allow hunting of waterfowl in the spring, and information about local subsistence will help us develop regulations.
2. To contribute to studies about the white-fronted geese (speckle bellies, orange-footed geese). Elder interviews and float and aerial surveys have shown that these geese have been declining in Interior Alaska.

Each interview takes about ten minutes. We ask about the kinds and numbers of ducks and geese taken. We also ask some questions about your hunting and use of ducks and geese. The surveyor is a local-hire employee from the region or another U.S. Fish and Wildlife Service employee together with a local resident from your community.

We do not record the names of people who participate. The information is collected anonymously and will not be used for any enforcement action.

Participation in the survey is voluntary. Households may decline any answer they wish.

Information collected from the harvest survey and from our float and aerial surveys is continuously provided to local tribal and city councils for community review.

Information from the spring 1998 and spring 1999 subsistence waterfowl harvest survey

	Galena		*Huslia*		*Nulato*		*Koyukuk*		*Kaltag*		*Hughes*		*Ruby*		*Total*	
	S 1998	*S 1999*	*S 1998*	*S 1999*	*S 1998*	*S 1999*	*S 1998*	*S 1999*	*S 1998*	*S 1999*	*S 1998*	*S 1999*	*S 1998*	*S 1999*	*S 1998*	*S 1999*
N of households interv	*95*	*99*	*53*	*52*	*71*	*83*	*35*	*32*	*46*	*53*	*13*	*18*	*48*	*56*	*361*	*393*
N. of households occu	*190*	*198*	*62*	*63*	*88*	*88*	*40*	*40*	*55*	*55*	*27*	*22*	*57*	*65*	*518*	*531*
**Species**																
**Swans**	-	-	1	-	-	-	1	-	1	-	-	-	-	-	*3*	-
Cranes																
Sandhill Crane	4	2	1		-		3		6		-	-	-	1	*14*	*3*
Geese																
Black Brant	-	-	-	6	-	-	-	-	-	-	-	-	-	-	-	*6*
Canada Goose	86	78	108	87	56	111	113	61	93	69	72	16	25	31	*553*	*453*
White-fronted Goose	36	34	109	123	34	147	57	88	23	56	19	24	7	1	*285*	*473*
Snow Goose	-	2	-	-	-	-	-	1	1	12	-	-	-	2	*1*	*17*
unidentified	4	-	6	10	13	-	6	-	4	-	5	23	-	-	*38*	*33*
total	126	114	223	226	103	258	176	150	121	137	96	63	32	34	*877*	*982*
Ducks																
American Wigeon	94	66	18	14	8	16	45	2	13	-	16	13	6	7	*200*	*118*
Bufflehead	2	-	-	-	-	-	1	5	1	-	80	-	-	1	*84*	*6*
Canvasback	-	-	-	-	-	2	-	-	-	-	-	-	-	-	-	*2*
Goldeneye	2	-	-	-	-	2	-	-	-	-	-	-	1	3	*3*	*5*
Green-winged Teal	8	2	5	3	1	5	1	3	1	-	5	-	2	-	*23*	*13*
Mallard	56	58	11	13	37	43	20	18	11	25	40	14	35	25	*210*	*196*
Northern Pintail	22	4	19	18	17	35	37	15	4	6	54	9	11	18	*164*	*105*
Northern Shoveler	20	2	-	-	5	5	1	3	8	2	10	-	3	-	*47*	*12*
Oldsquaw	-	-	5	2	-	-	1	2	-	-	30	-	-	-	*36*	*4*
Ring-necked duck	-	-	-	-	-	4	-	-	-	-	-	-	-	-	-	*4*
Scaup	22	-	-	-	-	-	-	-	-	-	-	-	-	-	*22*	-
Scoter	-	-	11	3	1	3	-	1	-	-	6	2	-	-	*18*	*9*
unidentified	10	-	4	1	-	47	4	33	5	-	15	-	1	-	*39*	*81*
total	236	132	73	54	69	162	110	82	43	33	256	38	59	54	*846*	*555*
others																
Merganser	-	-	-	-	2	-	-	-	1	-	-	-	-	-	*3*	-
unidentified waterfo	-	-	-	-	2	-	-	-	-	-	-	-	-	-	*2*	-
Grand total	366	248	298	280	176	420	290	232	172	170	352	101	91	89	*1745*	*1540*

Harvest numbers for Galena are doubled from harvest records from half of all households

Did the bird you harvested wear a band or a collar?

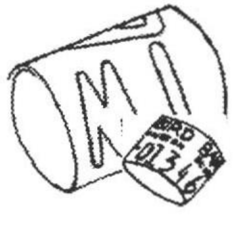

Some people reported that they had harvested banded or collared birds. We would like to encourage everybody to turn in bands or collars or to give us a call (1-800-656-1231) and tell us the number on the band or collar and when and where the bird was harvested. Our ducks and geese spend the winter season in the Lower 48 or in Mexico and South America. Recapture of banded birds at banding stations, observations of collared birds, and band or collar recoveries give us valuable information on the flight paths our birds take on their way down south.

Thank you for your help!

If you have any questions about the waterfowl harvest survey or bird bands and collars, please call:
Deborah Webb or Mike Spindler at 1-800-656-1231, or mail:
U.S. Fish and Wildlife Service, PO Box 287, Galena, AK 99741
